# Plasticity-induced actin polymerization in the dendritic shaft regulates intracellular AMPA receptor trafficking

**DOI:** 10.7554/eLife.80622

**Published:** 2024-08-15

**Authors:** Victor C Wong, Patrick R Houlihan, Hui Liu, Deepika Walpita, Michael C DeSantis, Zhe Liu, Erin K O'Shea

**Affiliations:** 1 Janelia Research Campus, Howard Hughes Medical Institute Ashburn United States; Max Planck Florida Institute for Neuroscience United States; Stanford University United States

**Keywords:** AMPA receptor, vesicle trafficking, synaptic plasticity, actin, CRISPR/Cas9, single-particle tracking, Rat

## Abstract

AMPA-type receptors (AMPARs) are rapidly inserted into synapses undergoing plasticity to increase synaptic transmission, but it is not fully understood if and how AMPAR-containing vesicles are selectively trafficked to these synapses. Here, we developed a strategy to label AMPAR GluA1 subunits expressed from their endogenous loci in cultured rat hippocampal neurons and characterized the motion of GluA1-containing vesicles using single-particle tracking and mathematical modeling. We find that GluA1-containing vesicles are confined and concentrated near sites of stimulation-induced structural plasticity. We show that confinement is mediated by actin polymerization, which hinders the active transport of GluA1-containing vesicles along the length of the dendritic shaft by modulating the rheological properties of the cytoplasm. Actin polymerization also facilitates myosin-mediated transport of GluA1-containing vesicles to exocytic sites. We conclude that neurons utilize F-actin to increase vesicular GluA1 reservoirs and promote exocytosis proximal to the sites of synaptic activity.

## Introduction

Synaptic plasticity – the modulation of synaptic connections in response to changes in neuronal activity – is regarded as the cellular basis for learning and memory (Abbott and Nelson, 2000; Citri and Malenka, 2008; Magee and Grienberger, 2020). Changes in postsynaptic spine morphology (i.e. structural plasticity) and protein composition are observed during synaptic plasticity and are thought to contribute to changes in synaptic transmission (i.e. functional plasticity; Kasai et al., 2010; Nakahata and Yasuda, 2018; Kasai, 2023). Persistent pre-synaptic neurotransmitter release stimulates postsynaptic spine enlargement and insertion of proteins into the synapse (Matsuzaki et al., 2004; Okamoto et al., 2004; Hayashi and Majewska, 2005; Patterson and Yasuda, 2011), including α-amino-3-hydroxy-5-methyl-4-isoxazolepropionic acid receptors (AMPARs; Song and Huganir, 2002) – ionotropic glutamate receptors that permit the influx of sodium, potassium, and in certain cases calcium ions (Chater and Goda, 2014). This increase in AMPAR abundance, as well as changes to AMPAR conductance, results in a greater inward flow of ions in response to stimulation (Anggono and Huganir, 2012; Diering and Huganir, 2018).

Given that the number of AMPARs at synapses is highly regulated during synaptic plasticity, AMPAR trafficking has been studied extensively (Malinow and Malenka, 2002; Henley et al., 2011; Park, 2018; Diering and Huganir, 2018; Groc and Choquet, 2020; Díaz-Alonso and Nicoll, 2021). The majority of AMPARs are synthesized in the cell body and then enter the secretory pathway, where they are trafficked in vesicles and inserted into the neuronal membrane via exocytosis (Shepherd and Huganir, 2007). AMPARs on the membrane can diffuse into synapses (Choquet and Opazo, 2022) and can also be endocytosed and delivered to lysosomes for degradation or recycled through the endocytic pathway (Ehlers, 2000; Carroll et al., 2001; Goo et al., 2017; Hanley, 2018). An important feature of synaptic plasticity is input specificity – plasticity stimulated at one synapse does not spread to inactive synapses (Kandel et al., 2013) – suggesting that AMPARs are inserted selectively into stimulated synapses. The mechanisms that regulate how AMPARs are delivered to synapses undergoing plasticity are not fully understood, but evidence points to two models: (1) AMPARs in vesicles are trafficked and exocytosed directly into or adjacent to stimulated synapses (Gerges et al., 2006; Kennedy et al., 2010; Cho et al., 2015; Park, 2018); and (2) AMPARs diffusing in the neuronal membrane are selectively trapped at stimulated synapses (Borgdorff and Choquet, 2002; Tardin et al., 2003; Petrini et al., 2009; Ehlers et al., 2007; Opazo et al., 2010; Opazo and Choquet, 2011; Penn et al., 2017; Choquet and Opazo, 2022).

AMPARs in vesicles are trafficked through dendrites by both microtubule- and actin-based motors, and disrupting active transport of AMPARs interferes with long-term potentiation (LTP), a commonly studied form of plasticity (Setou et al., 2002; Hoogenraad et al., 2005; Correia et al., 2008; Wang et al., 2008; Hoerndli et al., 2013; Hoerndli et al., 2015; Wagner et al., 2019; Gutiérrez et al., 2021). Nevertheless, it is unclear whether AMPAR-containing vesicles (hereafter referred to as AMPAR vesicles) are delivered directly to synapses undergoing plasticity, due in large part to limitations in imaging AMPARs in vesicles as opposed to on cell membranes (Groc and Choquet, 2020). One approach to studying AMPAR vesicles utilizes chemically inducible dimerization to control the release of exogenous AMPARs (i.e. AMPARs expressed from plasmid DNA) from the endoplasmic reticulum into the secretory pathway, followed by tracking AMPAR vesicles as they traverse photobleached sections of dendrite (Hangen et al., 2018; Bonnet et al., 2023). Using this technique, Hangen et al., 2018 found AMPAR vesicles slow down and pause in response to elevated intracellular calcium levels during neuronal activity, and consequently hypothesized that a calcium-mediated mechanism primes AMPAR vesicles for exocytosis. However, it is unclear if pausing is directly linked to AMPAR exocytosis at synapses undergoing plasticity. Additional studies have demonstrated that endosomes containing exogenous AMPARs can enter dendritic spines (EstevesdaSilva et al., 2015; Bowen et al., 2017), raising the possibility of direct AMPAR exocytosis into synapses. However, scanning electron micrographs of immunogold-labeled AMPARs fail to reveal a substantial fraction of AMPAR vesicles in spines (Tao-Cheng et al., 2011). Moreover, imaging exogenous AMPARs tagged with super ecliptic pHluorin shows that exocytosis occurs largely at extrasynaptic sites (Lin et al., 2009; Makino and Malinow, 2009; Patterson et al., 2010), favoring a model in which receptors diffuse into synapses after exocytosis.

Much research has been focused on understanding how synapses capture AMPARs as they diffuse through the neuronal membrane (Opazo and Choquet, 2011; Groc and Choquet, 2020; Choquet and Opazo, 2022). After exocytosis, AMPARs diffuse freely in random directions, but diffusion decreases precipitously at synapses (Tardin et al., 2003) because AMPARs are anchored there by postsynaptic density (PSD) proteins, such as PSD-95 (El-Husseini et al., 2000; Bats et al., 2007; Opazo et al., 2010; Opazo et al., 2012; Chen et al., 2015). Synaptic activity changes both the composition of proteins in the synapse and posttranslational modifications on AMPARs, further enhancing receptor anchoring (Opazo et al., 2010; Diering and Huganir, 2018; Lu and Roche, 2012; Opazo et al., 2012). These observations support a model where AMPARs may not be trafficked to specific loci, but rather diffuse in random directions, only to be concentrated in active synapses as a consequence of their increased residence time. Importantly, crosslinking AMPARs on the neuronal membrane to prevent their diffusion impairs synaptic potentiation in vivo (Penn et al., 2017; Getz et al., 2022). Nevertheless, the net distance a receptor can travel via diffusion is limited (Groc and Choquet, 2020). Consequently, this model depends on the presence of nearby extrasynaptic reservoirs from which synapses can draw AMPARs during plasticity (Choquet and Opazo, 2022). How reservoirs are established and maintained is not fully understood, but given that synapses can be located hundreds of microns from the cell body, it is probable that receptors are actively transported near sites undergoing synaptic plasticity.

To address whether and how neurons specify the location to which AMPAR vesicles are delivered, we developed a method to identify vesicles containing AMPAR GluA1 subunits expressed at native levels from endogenous loci and characterize the motion of these vesicles in cultured rat hippocampal neurons. Using this technique, we identify previously undescribed motion behaviors for GluA1-containing vesicles (hereafter referred to as GluA1 vesicles). We show that stimulating synaptic activity with glycine-induced chemical LTP (cLTP) or structural plasticity with glutamate uncaging-evoked structural LTP (sLTP) results in the local confinement of GluA1 vesicles in the dendritic shaft. We find that confinement concentrates GluA1 vesicles near sites of stimulation, thereby increasing the size of GluA1 reservoirs near these sites. GluA1 vesicle confinement is the result of stimulation-induced actin polymerization in the dendritic shaft, which changes the rheological properties of the dendritic cytoplasm in a manner that inhibits transport along the length of the dendrite and inhibits diffusion of GluA1 vesicles. Finally, we show that actin polymerization in the dendritic shaft near the sites of stimulation facilitates myosin-mediated transport of GluA1 vesicles from intracellular reservoirs to sites of exocytosis. In sum, our results suggest that neurons enhance the delivery AMPARs to active synapses by restricting the motion of AMPAR vesicles away from these synapses while simultaneously promoting AMPAR exocytosis near these synapses.

## Results

### GluA1-HaloTag is trafficked to postsynaptic densities and responds to stimulation

**Figure 1.**
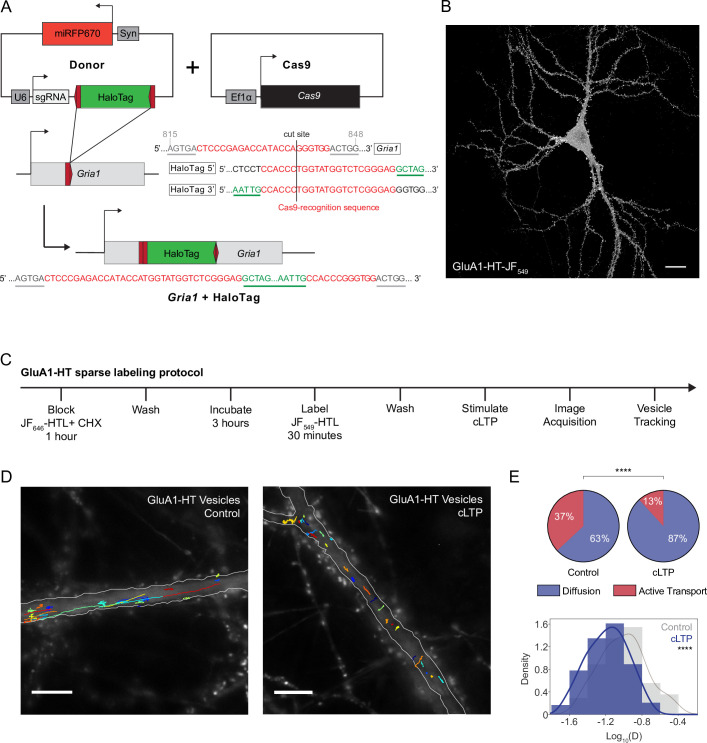
Chemical LTP induction reduces active transport and diffusion of GluA1-HT vesicles in the dendritic shaft. (**A**) Schematic of *Gria1* gene targeting with HaloTag (HT) by homology-independent targeted integration (HITI). A donor construct for HaloTag integration (Donor) is co-transfected into rat hippocampal neurons with a construct expressing Cas9 (Cas9). The donor contains HaloTag flanked on each side by one copy of the *Gria1* sequence to be targeted by Cas9, a single guide RNA (sgRNA), and a neuronal transfection marker (e.g. miRFP670). In neurons transfected with both the Cas9 and Donor constructs, Cas9 creates a double strand break in the genomic copy of *Gria1* and also excises the HaloTag sequence from the donor construct. The freed HaloTag sequence can be inserted into the genomic cut site when the double strand break is repaired by non-homologous end joining (NHEJ). (**B**) Representative confocal image of a cultured rat hippocampal neuron expressing endogenous GluA1 tagged with HaloTag and labeled with JF_549_-HaloTag ligand (GluA1-HT-JF_549_). Scale bar, 20 μm. (**C**) Experimental workflow to achieve sparse GluA1-HT labeling for GluA1-HT vesicle identification and single-particle tracking (SPT) analysis. (**D**) GluA1-HT vesicle trajectories in a dendritic shaft with no treatment (Control) and during cLTP induction (cLTP). Trajectories are overlaid on epifluorescence images of GFP-Homer1c, which was used to identify and segment the dendritic shaft. Scale bar, 10 μm. For videos, see Figure 1—video 5 and Figure 1—video 6. (**E**) Pie charts: fractions of vesicles exhibiting diffusion or active transport in dendritic shafts with no treatment (Control) and during cLTP induction (cLTP). ****p<0.0001 by Mann-Whitney test. n=12–14 timelapse imaging sequences (each timelapse captures the motion of GluA1-HT vesicles in one region of dendrite in one neuronal culture) for each condition. Histogram: distributions of diffusion coefficients for GluA1-HT vesicles without treatment (Control; gray) and during cLTP induction (cLTP; blue). Line represents the probability density function of each histogram estimated by kernel density estimation (KDE). ****p<0.0001 by Kolmogorov-Smirnov test. n=227–360 GluA1-HT vesicle trajectories pooled from 12 to 14 timelapses for each condition. Figure 1—source data 1.Related to Figure 1.

To study the intracellular transport of AMPARs during neuronal activity, we developed a method to label endogenous AMPAR GluA1 subunits (encoded by *Gria1*), expressed at native levels, using homology-independent targeted integration (HITI; Suzuki et al., 2016) in cultured rat hippocampal neurons. HaloTag (HT; Los et al., 2008) enables labeling of GluA1 with bright and photostable Janelia Fluor (JF) dyes (Grimm et al., 2015), which can be conjugated to the HaloTag ligand (HTL). We used HITI to insert HaloTag into the extracellular amino-terminal domain (NTD) of GluA1 at R280 (Figure 1A and Figure 1—figure supplement 1A), resulting in a high knock-in efficiency (Figure 1—figure supplement 1B–C). GluA1 edited at this position with HaloTag (GluA1-HT) and labeled with JF_549_-HaloTag ligand (JF_549_-HTL) is concentrated in dendritic spines, similar to endogenous GluA1 (Figure 1B; Craig et al., 1993). We find that the majority of edited sequences contain HaloTag in the correct orientation and without indels (Figure 1—figure supplement 1D), and that HaloTag insertion does not alter the expression of *Gria1* (Figure 1—figure supplement 2A–B). We observe correlated localization of HaloTag and *Gria1* mRNA in the same neuron (Figure 1—figure supplement 2C), indicating that *Gria1*-HaloTag is produced as a single transcript. Using stimulation emission depletion (STED) microscopy to examine GluA1 and HaloTag labeling at subdiffraction-limited length scales, we find that HaloTag signal has a strong overlap with native GluA1 signal (Figure 1—figure supplement 3A), indicating that GluA1-HT is translated as a single peptide sequence. Using immunofluorescence labeling and STED, we demonstrate that HaloTag insertion at R280 does not disrupt GluA1 trafficking to postsynaptic densities (Figure 1—figure supplement 3B–D). Finally, using electrophysiological methods, we demonstrate that insertion of HaloTag at R280 in the NTD of GluA1 does not significantly alter its channel function (Figure 1—figure supplement 4).

**Figure 1—figure supplement 1.**
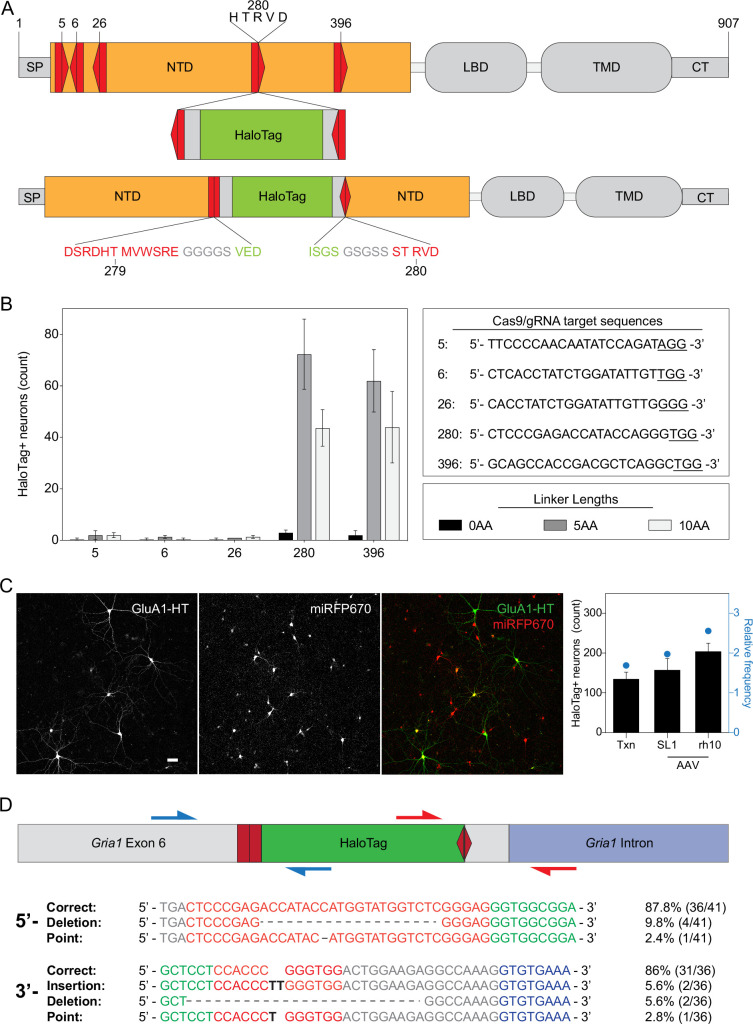
Successful insertion of HaloTag into *Gria1* depends on target site and linker length. (**A**) Schematic of GluA1 protein domains after HaloTag insertion via homology-independent targeted integration (HITI). SP, signal peptide; NTD, amino terminal domain; LBD, ligand binding domain; TMD, transmembrane domain; CT, C-terminus. The NTD and LBD are extracellular. Red pentagons represent the relative locations of attempted Cas9/guide RNA (gRNA) target sequences. Direction indicates whether the upper or lower DNA strand of *Gria1* was targeted (pointing right or left, respectively). Number indicates amino acid position. Copies of Cas9/gRNA target sequence flanking HaloTag sequence face the opposite direction as the genomic *Gria1* Cas9/gRNA target sequence. When HaloTag is inserted in the correct orientation, the Cas9/gRNA target sequence will not be restored, preventing Cas9 from cutting the same sites after repair. Gray rectangles flanking HaloTag represent peptide linkers. (**B**) Bar graph: mean number of cells with HaloTag knock-ins for different insertion sites and with different peptide linker lengths. n=3 independent transfections for each Cas9/gRNA target sequence and linker length. Error bars represent standard deviation. Top right panel: Cas9/gRNA target sequences with the protospacer adjacent motif (PAM) sequence underlined. Lower right panel: different linker lengths tested. Insertion at GluA1 280 R with a linker length of 5 amino acids was used for all GluA1 tagging experiments because it has the highest knock-in efficiency. (**C**) Images: representative widefield images of electroporated neurons expressing GluA1-HaloTag and labeled with JF_549_-HTL (GluA1-HT) or an miRFP670 neuronal transfection marker (miRFP670). Scale bar, 100 μm. Bar graph: efficiencies for HaloTag insertion for electroporation (txn) vs AAV-infection with SL1 or rh10 pseudotyped vectors. Bars represent the mean count and standard deviation for HaloTag positive neurons per dish. Blue dots represent relative efficiencies (HaloTag positive cells divided by cells transfected with both Donor and Cas9). n=6 independent transfections or infections for each condition. (**D**) Indel frequencies for *Gria1* with HaloTag insertions. Diagram: schematic of *Gria1* Exon 6 with HaloTag inserted by HITI. Genomic DNA was extracted from cultured neurons co-transfected with HaloTag donor and Cas9 expression constructs. *Gria1* sequences containing HaloTag knock-ins were amplified with primers flanking the 5’ insertion site (blue arrows) and the 3’ insertion site (red arrows). Amplicons were inserted into pUC expression vectors and then transformed into DH5α *E. coli*. DNA was then extracted from single colonies and sequenced to check for errors. To test the orientation of HaloTag knock-ins, we reversed the primers that target HaloTag, but did not amplify genomic DNA, indicating that the majority of HaloTag insertions are in the correct orientation. Sequences: 5’ and 3’ sequences of HaloTag correctly inserted into *Gria1* as well as examples of insertions, deletions, and point mutations, and the frequency of each type of error. Figure 1—figure supplement 1—source data 1.Related to Figure 1—figure supplement 1.

**Figure 1—figure supplement 2.**
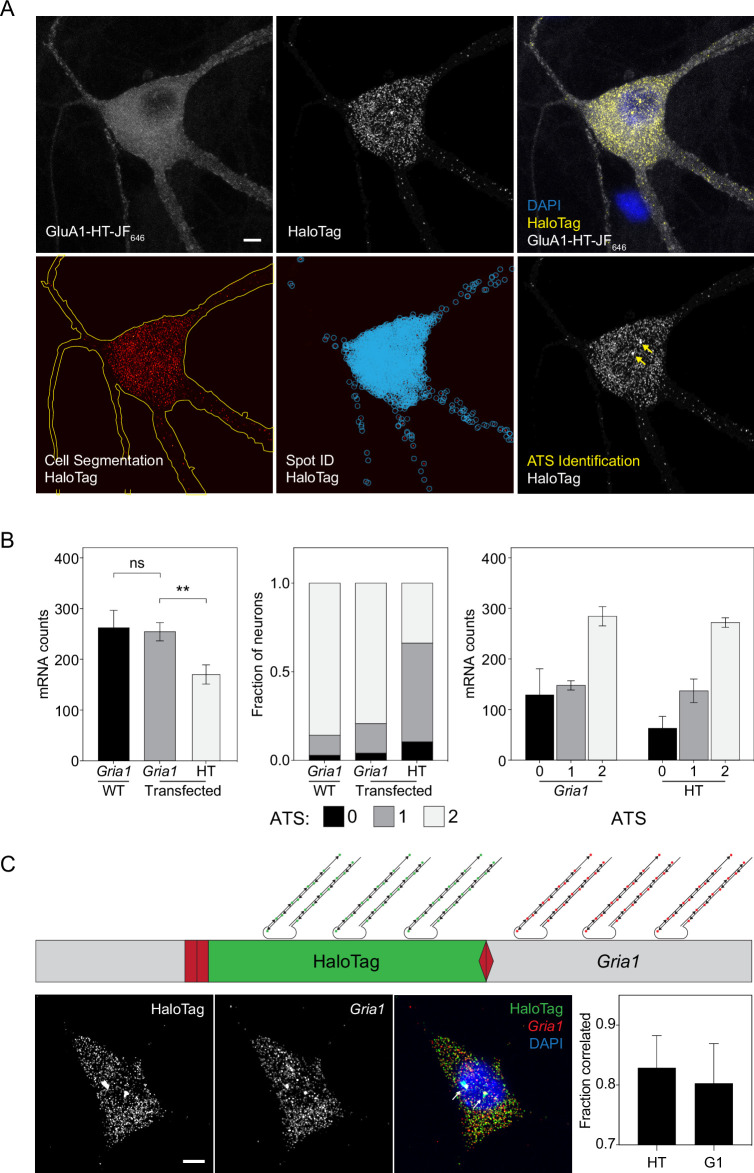
Identification and quantification of *Gria1-*HaloTag mRNA transcripts using HCR RNA-FISH. (**A**) Labeling HaloTag mRNA using hybridization chain reaction RNA fluorescence in situ hybridization (HCR RNA-FISH; Choi et al., 2018) followed by quantification using FISH-Quant (Mueller et al., 2013). Top row: representative confocal images of a cell expressing GluA1-HT labeled with JF_646_-HTL and also labeled with HCR RNA-FISH probes targeting HaloTag mRNA. Scale bar, 5 μm. Bottom row: neurons were segmented based on GluA1-HT-JF_646_ labeling, then mRNA spots and active transcription sites (ATS) were identified using FISH-Quant. (**B**) Left: mean number of *Gria1* mRNA per cell in untransfected neurons (WT) and *Gria1* and HaloTag mRNA per cell in transfected neurons. **p=0.0044 by t-test with Welch’s correction. n=3 independent HCR RNA-FISH labeling experiments for each condition. Center: fraction of cells that contain no ATS, one ATS, or two ATS when labeled with *Gria1* or HaloTag probes. Right: average number of mRNA spots per cell when cells are separated based on the number of ATS. There is no significant difference in the average number of mRNA spots per cell. Significance was determined by t-test with Welch’s correction. The initial quantification of *Gria1* and HaloTag mRNA revealed a significantly higher number of *Gria1* transcripts per cell when compared to HaloTag mRNA (left bar graph). This observation led us to hypothesize that for neurons expressing *Gria1*-HaloTag mRNA, many contain only one edited *Gria1* allele (i.e. HaloTag was only being expressed by one allele). Consequently, we counted the number of ATS per cell as a proxy for whether one or two alleles are edited (center bar graph). We then binned transcript counts based on the number of ATS a cell contains and found no significant difference. Moreover, we compared *Gria1* expression in untransfected neurons versus transfected neurons to ensure that HITI did not disrupt *Gria1* expression. We find a similar number of *Gria1* transcripts and ATS in transfected versus untransfected neurons. (**C**) Schematic: two-color HCR RNA-FISH labeling targeting *Gria1* and HaloTag mRNA to validate HaloTag knock-in. Probes conjugated to Alexa Fluor 488 (green) were used to label HaloTag mRNA while probes conjugated to Alexa Fluor 546 (red) were used to label *Gria1* mRNA. Images: representative confocal images of a two-color HCR RNA-FISH labeling experiment. Arrows indicate ATS. Scale bar, 5 μm. Bar graph: fraction of HaloTag mRNA spots located within 0.5 μm of *Gria1* mRNA spots, and fraction of *Gria1* mRNA spots located within 0.5 μm of HaloTag mRNA spots. n=7 labeled neurons. *Gria1* signals do not colocalize perfectly with HaloTag signals because probes targeting *Gria1* mRNA can be separated by a distance of up to 2940 basepairs from probes targeting HaloTag mRNA. A distance cutoff of 0.5 μm corresponds to a physical distance of 1470 linearized basepairs (half the maximum distance in which probes from the two channels can be separated if they are both on the same mRNA – that is if *Gria1*-HaloTag is synthesized as a single mRNA). Signals that fall within this distance cutoff have correlated localization and thus come from a single transcript (Materials and methods). Figure 1—figure supplement 2—source data 1.Related to Figure 1—figure supplement 2.

**Figure 1—figure supplement 3.**
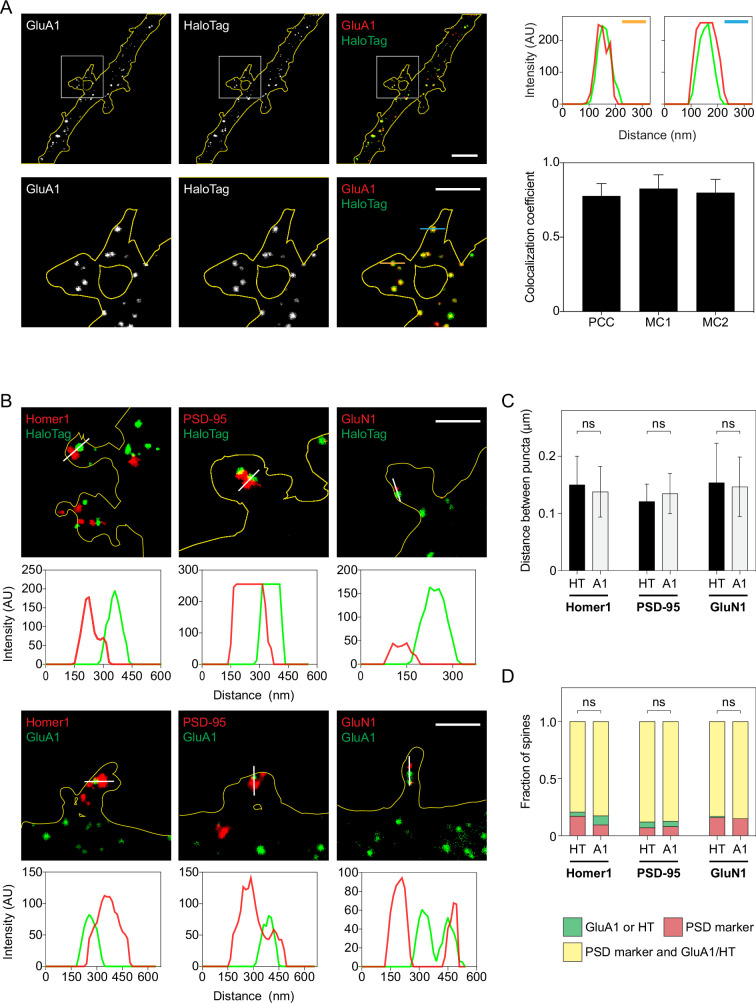
HaloTag is correctly inserted into the N-terminal domain of GluA1 and GluA1-HT is trafficked to postsynaptic densities. (**A**) Images: representative stimulation emission depletion (STED) images of a dendrite expressing endogenous GluA1 tagged with HaloTag and labeled with anti-GluA1 antibody (GluA1) and anti-HaloTag antibody (HaloTag). Bottom row: magnified images of spines. Scale bars, 2 μm. Line profiles: fluorescence intensity profiles of GluA1 and HaloTag signals denoted by orange and blue lines. Bar graph: Pearson correlation coefficients (PCC) and Manders’ overlap coefficients (MC1 and MC2) between HaloTag and GluA1 labeling. Bars represent mean and standard deviation. n=9 dendrite images (each image is of a region of dendrite from one neuron labeled with anti-GluA1 and anti-HaloTag antibodies). (**B**) Representative STED images of dendritic spines labeled with anti-HaloTag or anti-GluA1 antibodies and anti-Homer1, anti-PSD-95, or anti-GluN1 antibodies. Scale bars, 2 μm. Fluorescence intensity profiles (below images) denoted by white lines. (**C**) Mean distances between the centers of HaloTag (HT) or GluA1 (A1) fluorescence puncta and Homer1, PSD-95, or GluN1 fluorescence puncta. Error bars represent standard deviation. n=55–166 unique pairs of puncta from 9 to 12 dendrite images for each labeling combination (e.g. HaloTag and Homer1). Significance was determined by Mann-Whitney test. (**D**) Fractions of spines with only GluA1 or HaloTag; only a postsynaptic density (PSD) marker; or both GluA1 or HaloTag and a PSD marker. n=65–122 spines from 9 to 12 dendrite images for each labeling combination. Significance was determined by Mann-Whitney test. Using STED, we could distinguish structures at a resolution of 75 nm. At these length scales, GluA1/HaloTag does not perfectly colocalize with PSD markers. Nevertheless, we find that the average distance between HaloTag and PSD markers is similar to the average distance between GluA1 and PSD markers (**C**). Furthermore, HaloTag co-occurs in spines with PSD markers at the same rate as GluA1 (**D**). Combined, these observations demonstrate that GluA1-HT is trafficked to spines in a similar manner as GluA1. Figure 1—figure supplement 3—source data 1.Related to Figure 1—figure supplement 3.

**Figure 1—figure supplement 4.**
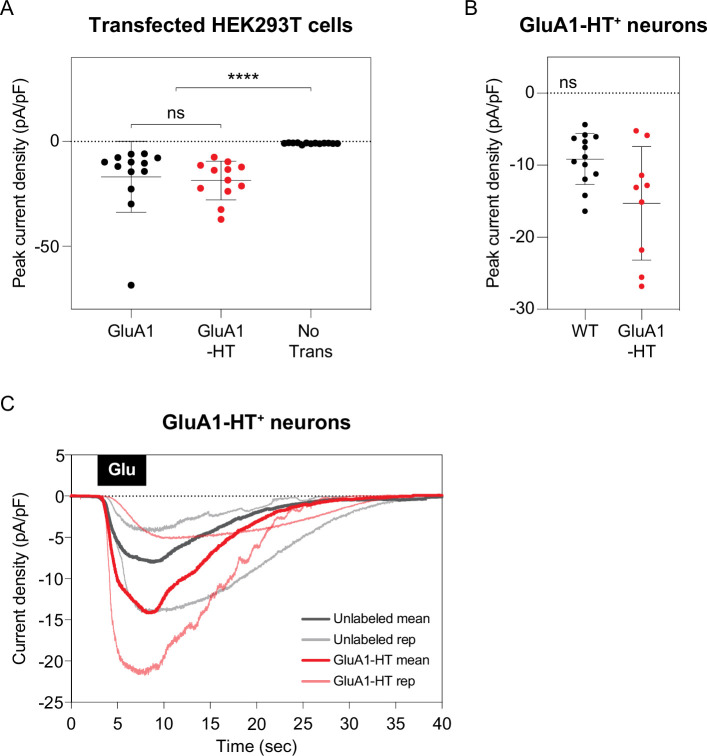
Currents elicited by GluA1-HT are similar to currents elicited by untagged GluA1. (**A**) Currents elicited by human embryonic kidney (HEK) 293T cells expressing GluA1 or GluA1-HT in response to locally perfused glutamate. Crosshairs represent mean and standard deviation of peak current densities (pA/pF) for each condition. Each dot represents a single recording. ****p<0.0001 by Mann-Whitney test. GluA1, n=13; GluA1-HT, n=12; No Transfection, n=13. Current densities for HEK293T cells were recorded following local application of 2 mM glutamate with 50 µM cyclothiazide for 10 s. (**B**) Currents elicited by GluA1-HT and unlabeled neurons in response to locally perfused glutamate. GluA1-HT, n=9; unlabeled, n=13. Significance was determined by Mann-Whitney test. Current densities for GluA1-HT neurons and unlabeled neurons were recorded following a 5 s 100 µM glutamate stimulation at a –70 mV holding potential. (**C**) Current density traces for GluA1-HT and unlabeled neurons in response to glutamate. Thin lines represent currents from two individual cells under each condition while thick lines represent mean currents. The small but notable reduction in mean trace peak amplitudes is a consequence of data decimation necessary for figure generation. Figure 1—figure supplement 4—source data 1.Related to Figure 1—figure supplement 4.

**Figure 1—figure supplement 5.**
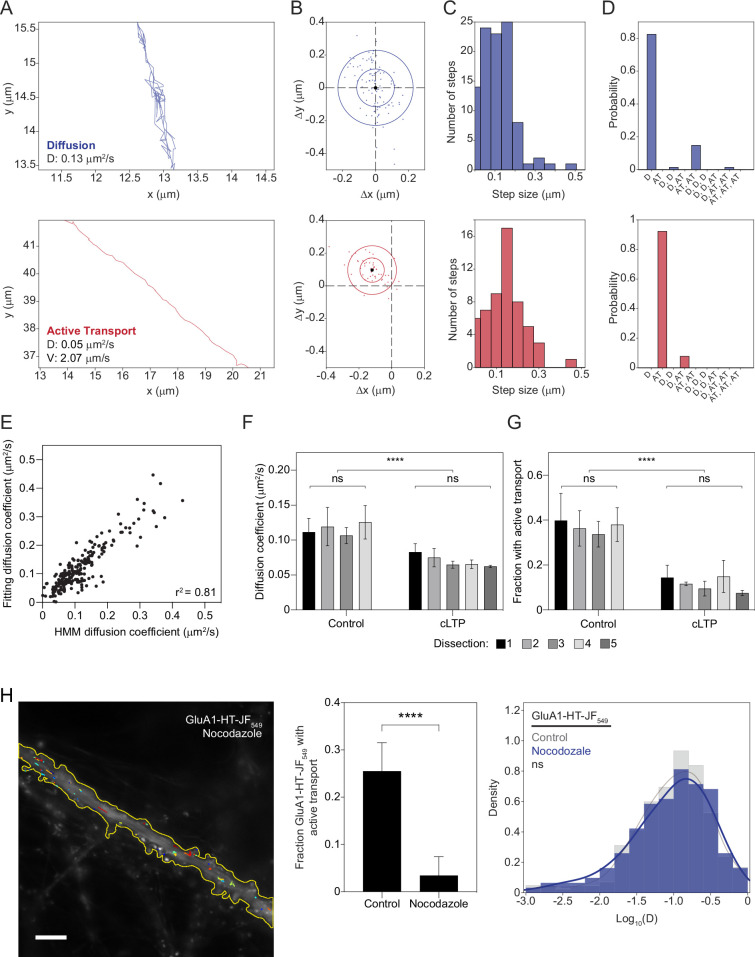
HMM-Bayes analysis can be used to infer the motion states of GluA1-HT-JF_549_ particles and determine motion parameters. (**A**) Representative trajectories for GluA1-HT-JF_549_ particles after block-and-chase protocol undergoing diffusion (top) and active transport (bottom). (**B**) Scatter plot of observed displacements (i.e. the vector between two successive points) along a trajectory for a particle exhibiting diffusion (top) or active transport (bottom). (**C**) Distributions of step sizes for the displacements presented in B. (**D**) Model probabilities for motion states (D, diffusion; AT, active transport) inferred by HMM-Bayes based on the observed displacements and step size distributions in (**B**) and (**C**). For a diffusive process, the displacements fit a normal distribution, from which HMM-Bayes can infer important motion parameters, such as the diffusion coefficient (D) and velocity (V; shown in A). Importantly, HMM-Bayes accounts for active transport by allowing for distributions of displacements with nonzero means (for example, B, bottom). (**E**) Scatterplot of diffusion coefficients for GluA1-HT-JF_549_ particles determined by fitting the linear portion of mean square displacement curves (y-axis) versus diffusion coefficients inferred by HMM-Bayes (x-axis). Diffusion coefficients determined by each method are highly correlated, demonstrating that HMM-Bayes accurately infers diffusion coefficients for diffusing trajectories. n=227 diffusion coefficients. (**F**) Mean diffusion coefficients for GluA1-HT-JF_549_ particles from different hippocampal dissections (i.e. different rats). Neurons from four different hippocampal dissections were used for Control experiments, while neurons from five different hippocampal dissections were used for chemical LTP (cLTP) stimulation experiments. For each dissection, we performed two to three timelapse imaging experiments to capture the motion of GluA1-HT-JF_549_ particles under the indicated conditions (Control versus cLTP). Error bars denote standard deviation. Significance between different dissections under the same treatment was determined by Kruskal-Wallis test. Control versus cLTP, ****p<0.0001. Significance between Control and cLTP was determined by Mann-Whitney test. (**G**) Fractions of GluA1-HT-JF_549_ particles with active transport from different hippocampal dissections. Error bars denote standard deviation. Significance between different dissections under the same treatment was determined by Kruskal-Wallis test. Control versus cLTP, ****p<0.0001. Significance between Control and cLTP was determined by Mann-Whitney test. These experiments (**F–G**) demonstrate that the variation in motion states and parameters between dissections is not significant. By contrast, variation due to cLTP stimulation is significantly greater than variation due to different dissections. Consequently, we can attribute differences in motion states and parameters to different treatment conditions rather than to biological differences between rats. (**H**) Images: trajectories for GluA1-HT-JF_549_ particles (after blocking with JF_646_-HTL and chasing with JF_549_-HTL) under Nocodazole treatment overlaid onto an epifluorescence image of GFP-Homer1c. GFP-Homer1c was used to define the boundaries of the dendritic shaft and spines. Scale bar, 5 μm. For video, see Figure 1—video 2. Bar graph: fraction of GluA1-HT-JF_549_ particles that exhibit active transport under control conditions versus Nocodazole treatment. ****p<0.0001 by Mann-Whitney test. n=8–10 timelapse imaging experiments for each condition. Each timelapse captures GluA1-HT-JF_549_ particle motion in one region of dendrite in one neuronal culture. Histogram: diffusion coefficients for GluA1-HT-JF_549_ particles under control conditions (Control; gray) versus Nocodazole treatment (Nocodazole; blue). n=154–209 GluA1-HT-JF_549_ particle trajectories pooled from 8 to 10 timelapses. Nocodazole treatment interferes with the polymerization of microtubules, thereby blocking kinesin- and dynein-based active transport. We demonstrate that Nocodazole dramatically reduces the fraction of GluA1-HT-JF_549_ particles that exhibit active transport based on HMM-Bayes analysis. Consequently, we believe that HMM-Bayes accurately infers motion states. Figure 1—figure supplement 5—source data 1.Related to Figure 1—figure supplement 5.

**Figure 1—figure supplement 6.**
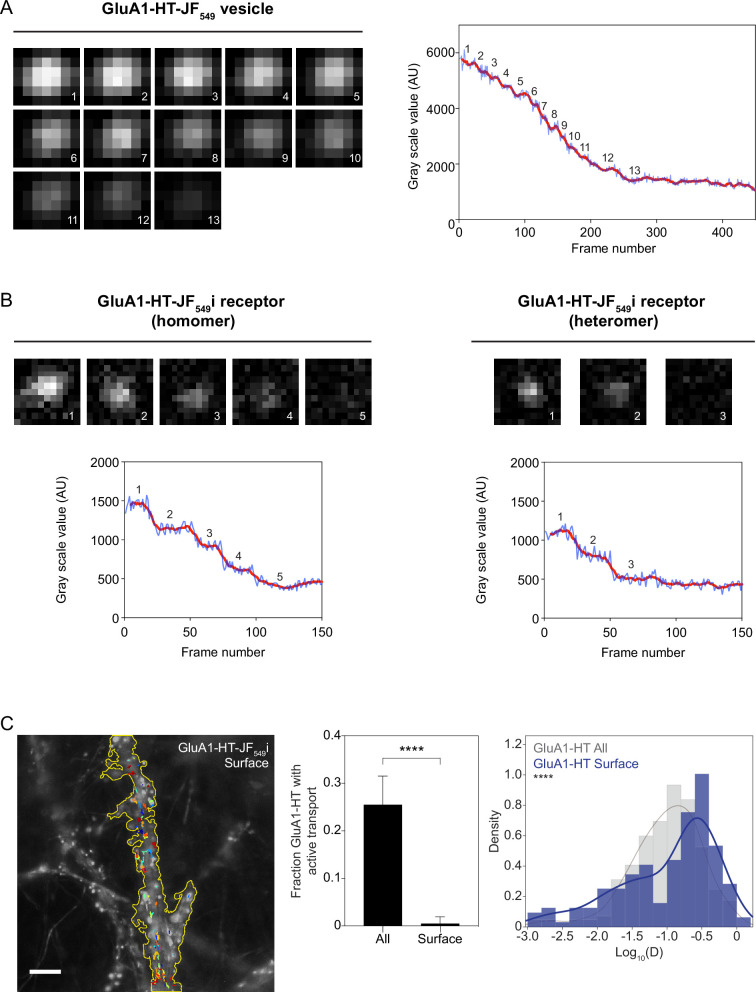
Identifying GluA1-HT vesicles based on their bleaching characteristics and motion. (**A**) Images: representative epifluorescence images of a GluA1-HT-JF_549_ vesicle as it is bleached due to repeated light exposure. Graph: mean fluorescence intensity of vesicle plotted against frame number. Red line represents a running average of 9 frames. Number indicates step that corresponds to representative image. Vesicles contain multiple GluA1-HT particles and therefore can bleach over many steps. (**B**) Images: representative epifluorescence images of a GluA1-HT-JF_549_i receptor on the surface of the cell membrane as it is bleached. GluA1-HT is labeled with a cell-membrane impermeable version of the JF_549_-HaloTag ligand (JF_549_i-HTL: ‘i’ for impermeable). Graphs: mean fluorescence intensity of receptor plotted against frame number. GluA1-HT-JF_549_i on the surface of dendritic shafts are individual receptors, which can contain up to four labeled subunits and therefore bleach in four steps (homomer, left). The most abundant AMPAR receptor type is the GluA1-GluA2 heterotetramer which will bleach in one to two steps (heteromer, right). (**C**) Images: trajectories for GluA1-HT-JF_549_i receptors (after blocking with JF_646_-HTL and chasing with JF_549_i-HTL) on the cell surface overlaid onto an epifluorescence image of GFP-Homer1c. Scale bar, 5 μm. For video, see Figure 1—video 3. Bar graph: fraction of GluA1-HT particles that exhibit active transport. All, GluA1-HT labeled with JF_549_-HTL (i.e. intracellular and surface GluA1-HT). Surface, GluA1-HT labeled with JF_549_i-HTL (i.e. surface GluA1-HT only). ****p<0.0001 by Mann-Whitney test. All, n=10 timelapse imaging experiments (each timelapse captures the motion of GluA1-HT-JF_549_ in one region of dendrite in one neuronal culture). Surface, n=8 timelapse imaging experiments. Histogram: diffusion coefficients for all GluA1-HT (All; gray) versus surface GluA1-HT (Surface; blue). ****p<0.0001 by Kolmogorov-Smirinov test. All, n=209 GluA1-HT-JF_549_ trajectories pooled from 10 timelapse experiments. Surface, n=159 GluA1-HT-JF_549_i trajectories pooled from eight timelapse experiments. In order to distinguish GluA1-HT vesicles from GluA1-HT receptors on neuronal surfaces, we labeled surface GluA1-HT receptors with JF_549_i-HTL, the cell-membrane impermeable variant of JF_549_-HTL, and characterized the bleaching rate and motion of surface receptors. These experiments demonstrate that surface receptors bleach much more rapidly than vesicles. We find that GluA1-HT surface receptors bleach within 100 frames. Importantly, GluA1-HT surface receptors very rarely exhibit active transport and generally have a higher diffusion coefficient than GluA1-HT vesicles unless GluA1-HT is trapped in a postsynaptic density (see Figure 1—figure supplement 7). GluA1-HT-JF_549_ particles (intracellular and surface) have a mean diffusion coefficient of 0.16±0.17 μm^2^/s, while GluA1-HT-JF_549_i particles (surface) have a mean diffusion coefficient of 0.22 ± 0.23 μm^2^/s. We consider particles with a diffusion coefficient of greater than 0.45 μm^2^/s (one standard deviation above the mean diffusion coefficient for GluA1-HT-JF_549_i particles) to likely be surface receptors. These features help us to further distinguish between the vesicular and surface population of GluA1-HT. Figure 1—figure supplement 6—source data 1.Related to Figure 1—figure supplement 6.

**Figure 1—figure supplement 7.**
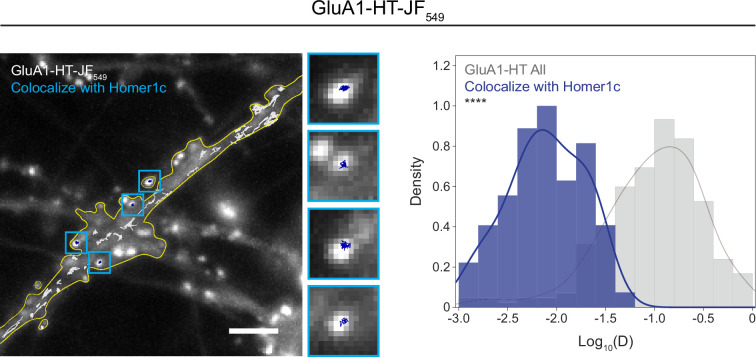
GluA1-HT-JF_549_ receptors trapped in postsynaptic densities have very low diffusion coefficients. Images: trajectories of GluA1-HT-JF_549_ particles (after blocking with JF_646_-HTL and chasing with JF_549_-HTL) overlaid onto an epifluorescence image of GFP-Homer1c. Blue squares are enlarged images of GluA1-HT trajectories that colocalize with GFP-Homer1c. Scale bar, 5 μm. For video, see Figure 1—video 4. Histogram: distributions of diffusion coefficients of GluA1-HT-JF_549_ particles that colocalize with GFP-Homer1c (Colocalize with Homer1c; blue) versus diffusion coefficients of all GluA1-HT-JF_549_ particles (All; gray). ****p<0.0001 by Kolmogorov-Smirnov test. n=209 trajectories for all GluA1-HT-JF_549_ particles and n=135 trajectories for GluA1-HT-JF_549_ particles that colocalize with Homer1c. Line represents the probability density function of each histogram estimated by kernel density estimation (KDE). GluA1-HT-JF_549_ particles that colocalize with Homer1c are likely receptors that are captured in postsynaptic densities, not receptors in vesicles, and therefore have very low diffusion coefficients (the mean diffusion coefficient for GluA1-HT-JF_549_ particles that colocalize with Homer1c is 0.01±0.009 μm^2^/s). Consequently, we filtered out GluA1-HT-JF_549_ particles from analysis (i.e. did not consider them to be vesicles) if they exhibited a diffusion coefficient below 0.02 μm^2^/s (one standard deviation above the mean diffusion coefficient for GluA1-HT-JF_549_ particles that colocalize with Homer1c). Figure 1—figure supplement 7—source data 1.Related to Figure 1—figure supplement 7.

**Figure 1—figure supplement 8.**
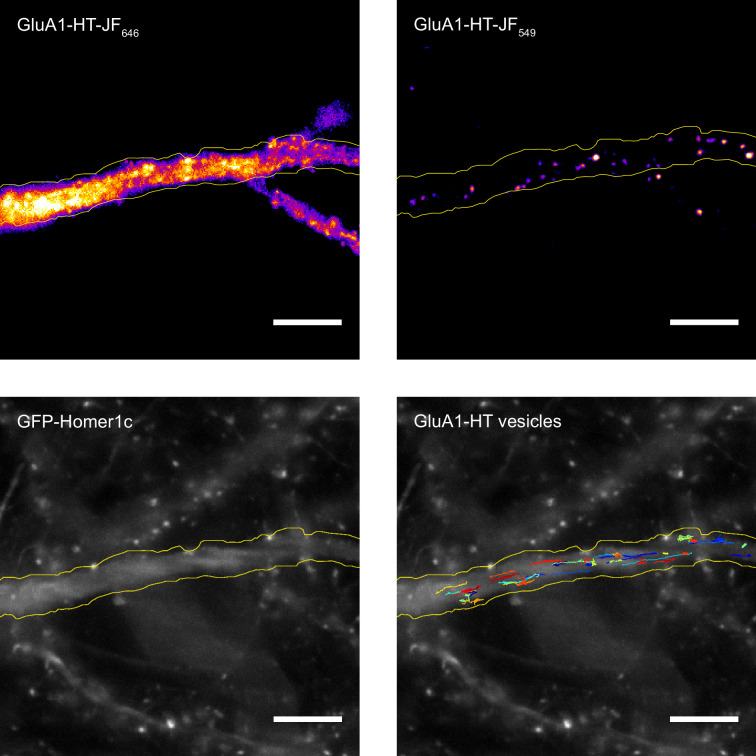
Block-and-chase protocol enables sparse labeling of GluA1-HT and the identification of GluA1-HT vesicles. Top row: representative widefield images of GluA1-HT blocked with JF_646_-HTL (GluA1-HT-JF_646_; top left) and chased with JF_549_-HTL (GluA1-HT-JF_549_; top right). Bottom row: epifluorescence image of GFP-Homer1c (GFP-Homer1c; bottom left) and trajectories for GluA1-HT vesicles (GluA1-HT vesicles; bottom right). Scale bar, 5 μm. For video, see Figure 1—video 1. To identify vesicles, we first filter particles that are clearly localized inside spines for the majority of steps in their trajectories. We then separate particles based on motion type (i.e. those that exhibit active transport). For particles that exhibit purely diffusive motion, we remove particles that bleach in fewer than 100 frames (i.e. the maximum number of frames required for a homomer with four labeled GluA1 subunits to bleach; Figure 1—figure supplement 6B). Lastly, we remove particles that have a diffusion coefficient greater than 0.45 μm^2^/s (i.e. particles on cell surface; Figure 1—figure supplement 6C) or less than 0.02 μm^2^/s (i.e. particles trapped in postsynaptic densities; Figure 1—figure supplement 7).

**Figure 1—figure supplement 9.**
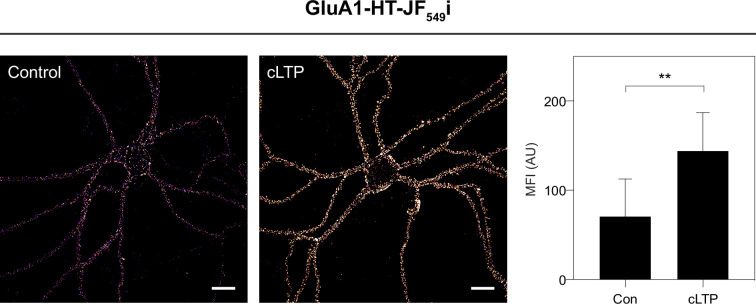
cLTP stimulation results in increased surface labeling of GluA1-HT by JF_549_i-HTL. Images: representative confocal images of the selective labeling of GluA1-HT on neuronal membranes using a membrane impermeable version of the JF_549_-HaloTag ligand (JF_549_i-HTL: ‘i’ for impermeable) without treatment (Control) and after cLTP (cLTP). Scale bars, 20 μm. Bar graph: mean fluorescence intensities (MFI) of JF_549_i-HTL-labeled GluA1-HT without treatment and after cLTP. Bars represent mean and standard deviation, **p=0.0047 by Mann-Whitney test. n=8 labeled neurons from three independent neuronal cultures for each condition. Figure 1—figure supplement 9—source data 1.Related to Figure 1—figure supplement 9.

**Figure 1—figure supplement 10.**
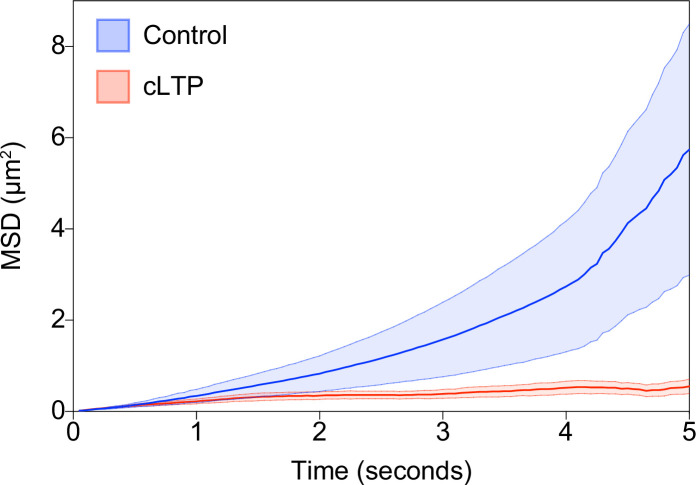
GluA1-HT vesicles exhibit subdiffusive motion during cLTP induction. Mean square displacement (MSD) of GluA1-HT vesicles under no treatment control condition (Control; blue) and during cLTP induction (cLTP; red). Thick lines represent mean while area represents standard error. n=41–48 Glu1-HT vesicle trajectories for each condition. Figure 1—figure supplement 10—source data 1.Related to Figure 1—figure supplement 10.

**Figure 1—figure supplement 11.**
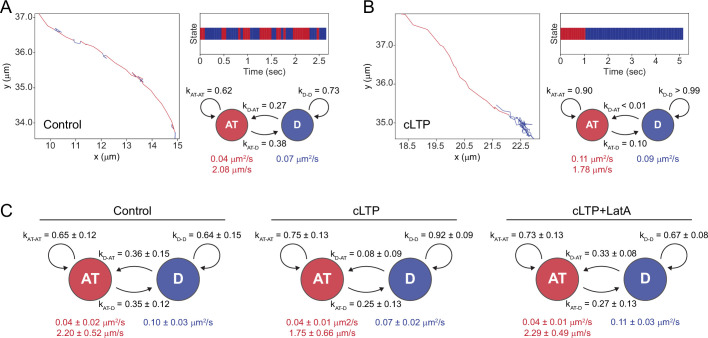
cLTP induction increases the probability GluA1-HT vesicles switch from active transport to diffusion. (**A**) Left: representative trajectory of a GluA1-HT vesicle under control conditions exhibiting stochastic switching between active transport (AT) and diffusive motion (D). Right top: temporal state sequence inferred by HMM-Bayes of the trajectory depicted on the left. Right bottom: transition probabilities and motion parameters for each motion state for the trajectory depicted on the left. k_AT-AT_, probability that a GluA1-HT vesicle remains in active transport; k_AT-D_, probability that a vesicle switch from active transport to diffusion; k_D-AT_, probability that a vesicle switch from diffusion to active transport; k_D-D_, probability that a vesicle remains in diffusion. Values in red are the velocity and diffusion coefficient of the vesicle during active transport while the value in blue is the diffusion coefficient of the vesicle during diffusion. (**B**) Left: representative trajectory of a GluA1-HT vesicle under cLTP induction exhibiting stochastic switching between active transport and diffusive motion. Right top: temporal state sequence inferred by HMM-Bayes of the trajectory depicted on the left. Right bottom: transition probabilities and motion parameters for each motion state for the trajectory depicted on the left. (**C**) Mean state-switching probabilities for multi-state GluA1-HT vesicles under control conditions (Control), cLTP induction (cLTP), and cLTP induction in the presence of Latrunculin A (cLTP +LatA). Control, k_AT-D_ vs k_D-AT_, p=0.8065; cLTP, k_AT-D_ vs k_D-AT_, ****p<0.0001; cLTP +LatA, k_AT-D_ vs k_D-AT_, *p=0.0348. Significance was determined by Mann-Whitney test. n=26–30 multi-state GluA1-HT vesicles trajectories for each condition. Figure 1—figure supplement 11—source data 1.Related to Figure 1—figure supplement 11.

**Figure 1—video 1.** Timelapse sequence of GluA1-HT-JF_549_ after block-and-chase labeling with JF_646_-HTL and JF_549_-HTL in the dendrite of a cultured rat hippocampal neuron. Scale bar, 5 μm. Top left: all GluA1-HT labeled with JF_646_-HTL (block; GluA1-HT-JF_646_). Top right: newly synthesized GluA1-HT labeled with JF_549_-HTL (chase; GluA1-HT-JF_549_). Bottom left: GFP-Homer1c. Bottom right: GluA1-HT-JF_549_ (green) overlaid onto GFP-Homer1c (magenta).

**Figure 1—video 2.** Timelapse sequence of GluA1-HT-JF_549_ after block-and-chase labeling with JF_646_-HTL and JF_549_-HTL in a dendrite that has been treated with Nocodazole. Scale bar, 5 μm. Left: GFP-Homer1c. Center: GluA1-HT-JF_549_ (chase). Right: GluA1-HT-JF_549_ (green) overlaid onto GFP-Homer1c (magenta). Related to Figure 1—figure supplement 5.

**Figure 1—video 3.** Timelapse sequence of GluA1-HT-JF_549_i on the surface of a dendrite after block-and-chase labeling with JF_646_-HTL and JF_549_i-HTL. Scale bar, 5 μm. Left: GFP-Homer1c. Center: GluA1-HT-JF_549_i (chase; surface). Right: GluA1-HT-JF_549_i (green) overlaid onto GFP-Homer1c (magenta). Related to Figure 1—figure supplement 6.

**Figure 1—video 4.** Timelapse sequence of GluA1-HT-JF_549_ after block-and-chase labeling with JF_646_-HTL and JF_549_-HTL, with GluA1-HT-JF_549_ particles that colocalize with GFP-Homer1c highlighted. Scale bar, 5 μm. Left: GFP-Homer1c. Center: GluA1-HT-JF_549_ (chase). Right: GluA1-HT-JF_549_ (green) overlaid onto GFP-Homer1c (magenta). Blue boxes highlight GluA1-HT-JF_549_ particles that colocalize with GFP-Homer1c. Related to Figure 1—figure supplement 7.

**Figure 1—video 5.** Timelapse sequence of GluA1-HT-JF_549_ after block-and-chase labeling with JF_646_-HTL and JF_549_-HTL in a dendrite with no stimulation. Scale bar, 5 μm. Left: GFP-Homer1c. Center: GluA1-HT-JF_549_ (chase). Right: GluA1-HT-JF_549_ (green) overlaid onto GFP-Homer1c (magenta).

**Figure 1—video 6.** Timelapse sequence of GluA1-HT-JF_549_ after block-and-chase labeling with JF_646_-HTL and JF_549_-HTL in a dendrite during cLTP induction. Scale bar, 5 μm. Left: GFP-Homer1c. Center: GluA1-HT-JF_549_ (chase). Right: GluA1-HT-JF_549_ (green) overlaid onto GFP-Homer1c (magenta).

### Neuronal activity confines GluA1 vesicles by disrupting vesicle motion in the dendritic shaft of cultured rat hippocampal neurons

To overcome the challenge of signal saturation (Figure 1B) and track single GluA1-HT vesicles inside the dendritic shaft, we developed a block-and-chase protocol to achieve sparse labeling of de novo synthesized endogenous GluA1 (Figure 1C). To avoid visualizing pre-existing GluA1-HT, GluA1-HT was first labeled with a saturating concentration of JF_646_-HTL in the presence of the translation inhibitor cycloheximide (CHX). Next, JF_646_-HTL and CHX were washed away, and after an incubation period to allow GluA1-HT translation to recover, de novo synthesized GluA1-HT was labeled with JF_549_-HTL. Because JF_549_-HTL labels only newly synthesized GluA1-HT, signal detected by fluorescence microscopy is dramatically reduced in this fluorescence channel, allowing us to identify sparse, punctate GluA1-HT-JF_549_ conjugates (Figure 1—video 1). We then used single-particle tracking (SPT) analysis to reconstruct trajectories, and hidden Markov modeling with Bayesian model selection (HMM-Bayes; Monnier et al., 2015) to determine important motion parameters (i.e. velocity and diffusion coefficient) and predict the motion type (i.e. active transport versus diffusion) of each trajectory (Figure 1—figure supplement 5; Jaqaman et al., 2008; Liu et al., 2018). Finally, we separated GluA1-HT vesicles from surface GluA1-HT based on motion type, fluorescence bleaching characteristics, and localization in the dendritic shaft (Figure 1—figure supplements 6–8).

Having established a pipeline to label, track, and analyze GluA1-HT vesicles, we sought to evaluate whether the motion of GluA1-HT vesicles is modulated by synaptic activity. Glycine-induced chemical LTP (cLTP) is an established method of stimulating synaptic plasticity and increasing the surface expression of GluA1 in cultured neurons (Lu et al., 2001; Passafaro et al., 2001; Park et al., 2006; Molnár, 2011). We hypothesized that cLTP induction might change the motion of GluA1-HT vesicles in the dendritic shaft to support increased GluA1-HT exocytosis during neuronal activity. Using a membrane impermeable variant of JF_549_-HTL termed JF_549_i-HaloTag ligand (JF_549_i-HTL; ‘i’ for impermeant; Xie et al., 2017), we validated that cLTP stimulates increased expression of GluA1-HT on neuronal surfaces (Figure 1—figure supplement 9). We then imaged and tracked GluA1-HT vesicles in dendrites during cLTP induction, and observed clear qualitative differences in motion compared to vesicles in the dendrites of unstimulated control neurons (Figure 1D, Figure 1—video 5 and Figure 1—video 6). Most strikingly, we observed a loss in long-range motion along the length of the dendritic shaft in cLTP-stimulated neurons. When HMM-Bayes is applied to characterize trajectories collected during cLTP induction, we find a significant decrease in the fraction of GluA1-HT vesicles undergoing active transport (Figure 1E, pie charts). The diffusion coefficients (D) for GluA1-HT vesicles undergoing diffusion are also significantly reduced by cLTP induction (Figure 1E, histogram). In addition, vesicles exhibit increased subdiffusion (i.e. constrained diffusion due to molecular crowding or interactions; Feder et al., 1996; Saxton, 2007) in response to cLTP induction (Figure 1—figure supplement 10).

These observations demonstrate that the overall motion of GluA1-HT vesicles is inhibited by cLTP, suggesting that vesicle motion is locally confined (defined in this work as the restriction of vesicle motion away from its initial position). We reasoned that if cLTP spatially confines GluA1-HT vesicles then it should, in addition to decreasing the fraction of vesicles undergoing active transport, prevent diffusing vesicles from transitioning to active transport and leaving their local regions. A critical feature of HMM-Bayes is the ability to infer multiple motion states from a single trajectory and determine state-transition probabilities (Figure 1—figure supplement 11A–B; Monnier et al., 2015). Using HMM-Bayes, we find multi-state GluA1-HT vesicles stochastically switch motion states from active transport to diffusion and from diffusion to active transport with approximately the same probability under unstimulated control conditions (Figure 1—figure supplement 11C, Control, k_AT-D_ vs k_D-AT_). By contrast, during cLTP, GluA1-HT vesicles have a greater probability of switching from active transport to diffusion than from diffusion to active transport (Figure 1—figure supplement 11C, cLTP, k_AT-D_ vs k_D-AT_). Furthermore, GluA1-HT vesicles undergoing diffusion have a high probability to continue diffusing (Figure 1—figure supplement 11C, cLTP, k_D-D_). Taken together, our observations suggest that cLTP induction results in the local confinement of GluA1-HT vesicles.

### Local induction of synaptic activity confines GluA1 vesicles near the site of activity by disrupting GluA1 vesicle motion

**Figure 2.**
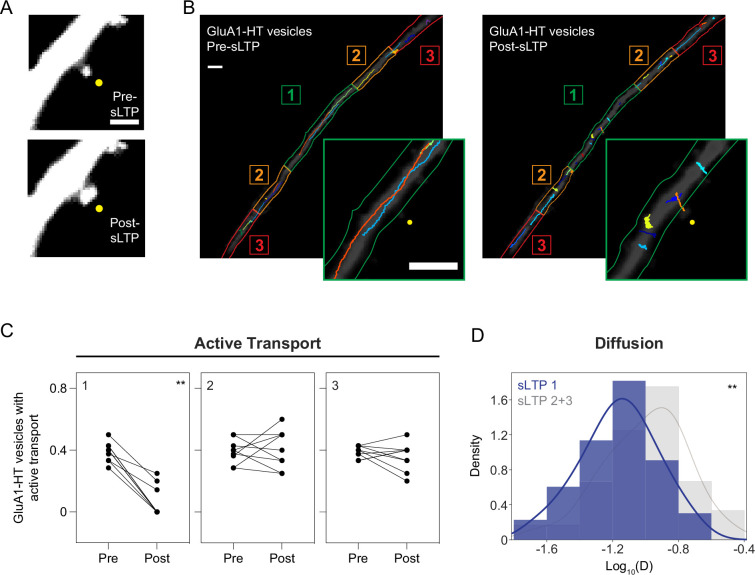
Structural LTP stimulation reduces active transport and diffusion of GluA1-HT vesicles proximal to the site of synaptic activity. (**A**) Representative epifluorescence images of a dendritic spine expressing GFP immediately before sLTP (Pre-sLTP) and after sLTP (Post-sLTP). Yellow dot indicates the site of uncaging. Scale bar, 2 μm. (**B**) GluA1-HT vesicle trajectories immediately before sLTP (Pre-sLTP) and after sLTP (Post-sLTP) overlaid on a dendrite expressing GFP. Dendrite is separated into three equal zones based on proximity to the stimulated spine to distinguish proximal and distal areas (Materials and methods). Insets: magnified images of trajectories near the site of uncaging (yellow dot). Scale bars, 5 μm. For video, see Figure 2—video 1. (**C**) Fractions of GluA1-HT vesicles exhibiting active transport pre- and post-sLTP in each zone. **p=0.0039 by Wilcoxon matched pairs test. Each dot represents the fraction of vesicles exhibiting active transport in the indicated zone for one sLTP stimulation experiment. n=9 sLTP stimulation experiments (each experiment targets one spine on one neuron) where GluA1-HT vesicle motion in the dendrite is captured immediately before and after sLTP stimulation. (**D**) Distributions of diffusion coefficients for GluA1-HT vesicles in Zone 1 (sLTP 1; blue) versus Zone 2+3 (sLTP 2+3; gray) after sLTP stimulation. Line represents the probability density function of each histogram estimated by kernel density estimation (KDE). **p=0.0037 by Kolmogorov-Smirnov test. n=60–66 GluA1-HT vesicle trajectories pooled together from nine sLTP stimulation experiments. Figure 2—source data 1.Related to Figure 2.

If confinement is a mechanism to increase the intracellular reservoir of GluA1-HT near sites of synaptic activity, then stimulating plasticity at a specific synapse should alter GluA1-HT vesicle motion near that synapse. Single-photon (1 P) 4-Methoxy-7-nitroindolinyl-caged-ʟ-glutamate (MNI) uncaging can be used to stimulate synaptic activity in a desired region (Ellis-Davies, 2007). We used a glutamate-uncaging evoked structural LTP protocol (sLTP; Matsuzaki et al., 2004) to induce structural plasticity at a spine of interest. This protocol has been shown to stimulate N-methyl-ᴅ-aspartate receptor (NMDAR)-mediated calcium transients, which result in spine expansion and increased AMPAR concentration at the targeted synapse – two important proxies for functional plasticity (Matsuzaki et al., 2001; Matsuzaki et al., 2004; Lee et al., 2009; Patterson and Yasuda, 2011; Huganir and Nicoll, 2013; Bosch et al., 2014; Kruijssen and Wierenga, 2019). First, we calibrated the strength of the laser so that it would not trigger calcium influx or structural plasticity in neighboring spines. In targeted spines, we observed a significant increase in the area of the spine head and in GluA1-HT after stimulation in dishes containing MNI, but not in dishes without MNI (Figure 2A and Figure 2—figure supplements 1–2). The increase in spine size persists 10 min after the cessation of sLTP, indicating that this protocol induces sustained changes to activity (Figure 2—figure supplement 3A, images and Spine area line graph).

**Figure 2—figure supplement 1.**
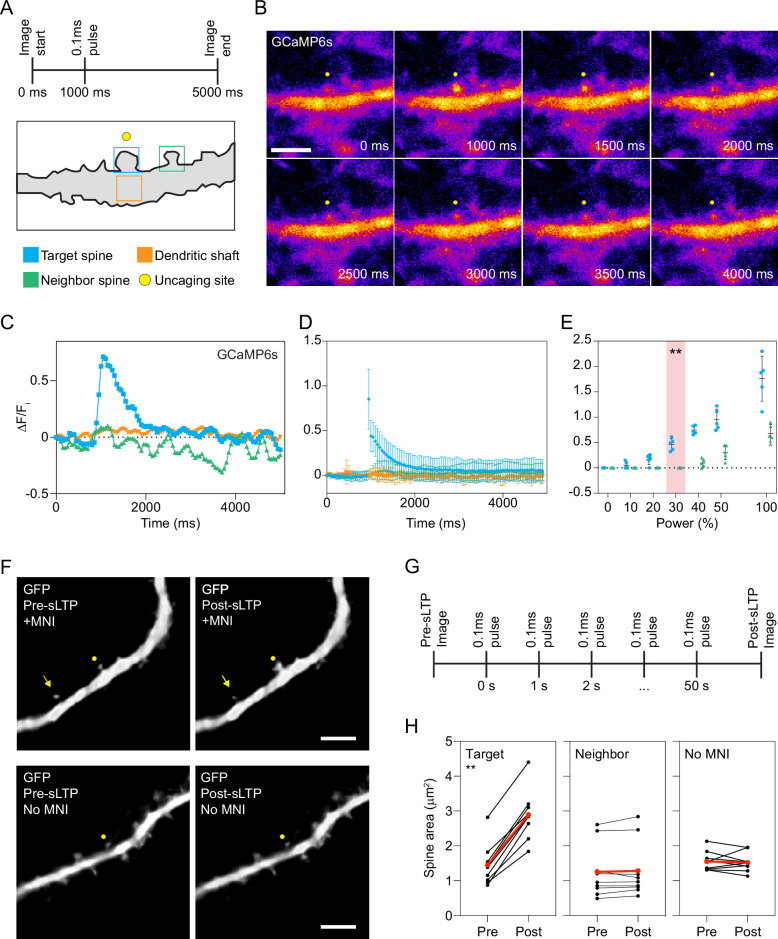
MNI uncaging calibration. (**A**) Top: laser stimulation protocol for MNI-caged-ʟ-glutamate (MNI) uncaging. Bottom: diagram of a dendrite and regions where GCaMP6s (Chen et al., 2013) fluorescence was measured over time (colored boxes) to calibrate laser power. (**B**) Representative epifluorescence timelapse images of GCaMP6s after MNI uncaging. Yellow dot indicates uncaging site. Scale bar, 5 μm. (**C**) Change in GCaMP6s fluorescence intensity (ΔF/F_i_) over time in the regions depicted in (**A**) after MNI uncaging. (**D**) Mean ΔF/F_i_ for GCaMP6s over time in a targeted spine (blue), in the dendritic shaft below the target spine (orange), or in a neighboring spine (green) after MNI uncaging. Error bars represent standard deviation. n=9 MNI uncaging timelapse imaging experiments. For each experiment, one spine is stimulated using the uncaging protocol depicted in (**A**). A new neuron is selected for each experiment. (**E**) Peak ΔF/F_i_ for GCaMP6s in a targeted spine (blue) or a neighboring spine (green) after MNI uncaging with increasing laser power. Crosshairs represent mean and standard deviation, and each dot represents a single uncaging experiment. Red bar indicates the laser power at which there was maximum activation of GCaMP6s in the target spine without appreciable activation of GCaMP6s in the neighboring spines. **p=0.0079 by Mann-Whitney test. n=5 MNI uncaging experiments per laser power. (**F**) Representative epifluorescence images of a dendritic spine immediately before sLTP (Pre-sLTP) and after sLTP (Post-sLTP) in the presence (above) or absence (below) of MNI. Yellow dot indicates uncaging site; yellow arrow indicates neighboring spine. Scale bars, 5 μm. (**G**) sLTP stimulation protocol to induce structural plasticity in dendritic spines. (**H**) Area of dendritic spines targeted for sLTP stimulation (left) and neighboring spines (middle) pre- and post-sLTP. Area of dendritic spines targeted for sLTP stimulation in the absence of MNI (right). Red dots and lines represent the mean spine area pre- and post-sLTP. **p=0.0032 by Wilcoxon matched pairs test. n=9 sLTP stimulation experiments (each sLTP experiment targets one spine on one neuron) for each condition. Figure 2—figure supplement 1—source data 1.Related to Figure 2—figure supplement 1.

**Figure 2—figure supplement 2.**
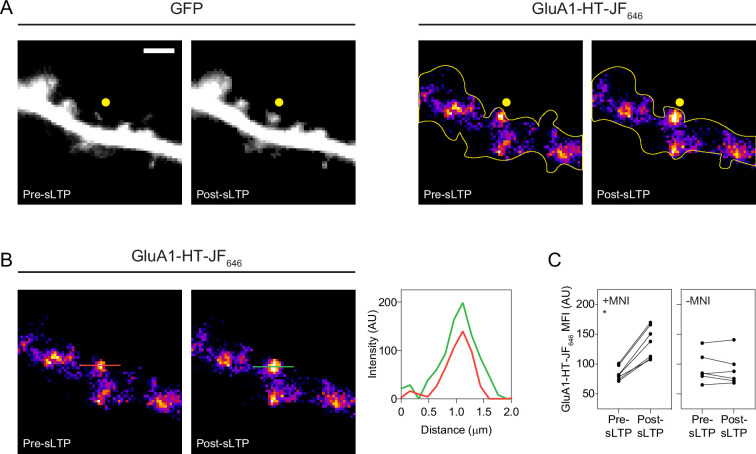
sLTP stimulation increases GluA1-HT-JF_646_ in targeted spines. (**A**) GFP: representative epifluorescence images of a dendritic spine expressing GFP immediately before sLTP (Pre-sLTP) and after sLTP (Post-sLTP). Yellow dot indicates the site of uncaging. Scale bar, 2 μm. GluA1-HT-JF_646_: representative epifluorescence images of GluA1-HT-JF_646_ in dendritic spine pre- and post-sLTP. (**B**) Line profiles: fluorescence intensity profiles of GluA1-HT-JF_646_ signals denoted by red (Pre-sLTP) and green (Post-sLTP) lines. (**C**) Line graph of GluA1-HT-JF_646_ mean fluorescence intensity (MFI) pre- and post-sLTP in the presence or absence of MNI. *p=0.0156 by Wilcoxon matched pairs test. n=6–7 sLTP stimulation experiments (each sLTP experiment targets one spine on one neuron) for each condition. Figure 2—figure supplement 2—source data 1.Related to Figure 2—figure supplement 2.

**Figure 2—figure supplement 3.**
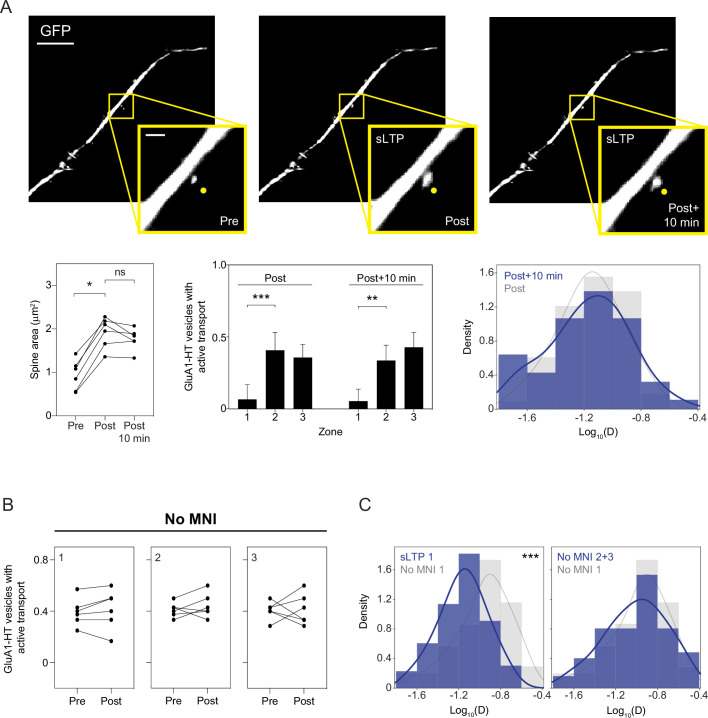
Motion of GluA1-HT vesicles 10 min after the cessation of sLTP stimulation is similar to the motion of vesicles immediately after sLTP stimulation. (**A**) Images: representative epifluorescence images of a dendritic spine immediately before sLTP (Pre-sLTP), immediately after sLTP (Post-sLTP), or 10 min after sLTP (Post +10 min). Scale bar, 10 μm. Insets: enlarged images of dendritic spine. Scale bar, 2 μm. Yellow dot, uncaging site. Left line graph: Spine areas of targeted spines pre-sLTP, post-sLTP, and 10 min after the cessation of sLTP. Pre vs Post, *p=0.0312. Significance was determined by Wilcoxon matched pairs test. n=6 sLTP stimulation experiments (each sLTP experiment targets one spine on one neuron). Center bar graph: mean fractions of GluA1-HT vesicles exhibiting active transport immediately after sLTP (Post) or 10 min after sLTP (Post +10 min) in each zone. Error bars represent standard deviation. Post: Zone 1 vs Zone 2, ***p=0.0001. Post +10 min: Zone 1 vs Zone 2, **p=0.0022. Significance was determined by Mann-Whitney test. n=6–9 sLTP stimulation experiments where GluA1-HT vesicle motion in the dendrite is captured immediately after sLTP stimulation or 10 min after the cessation of sLTP stimulation. Right histogram: distributions of diffusion coefficients in Zone 1 immediately after sLTP stimulation (Post; gray) versus 10 min after sLTP (Post +10 min; blue). Line represents the probability density function of each histogram estimated by kernel density estimation (KDE). n=47–66 GluA1-HT vesicle trajectories pooled from six to nine sLTP stimulation experiments for each condition. (**B**) Fractions of GluA1-HT vesicles exhibiting active transport pre- or post-sLTP stimulation in each zone in the absence of MNI. n=6 sLTP stimulation experiments. (**C**) Left: distributions of diffusion coefficients for GluA1-HT vesicles in Zone 1 after sLTP (sLTP 1; blue) versus vesicles in Zone 1 after stimulation in the absence of MNI (No MNI 1; gray). ***p=0.0002 by Kolmogorov-Smirnov test. Right: diffusion coefficients for GluA1-HT vesicles in Zone 1 (No MNI 1; gray) versus Zone 2+3 (No MNI 2+3; blue) after sLTP in the absence of MNI. n=52–66 GluA1-HT vesicle trajectories pooled from six to nine sLTP stimulation experiments for each condition. Figure 2—figure supplement 3—source data 1.Related to Figure 2—figure supplement 3.

**Figure 2—figure supplement 4.**
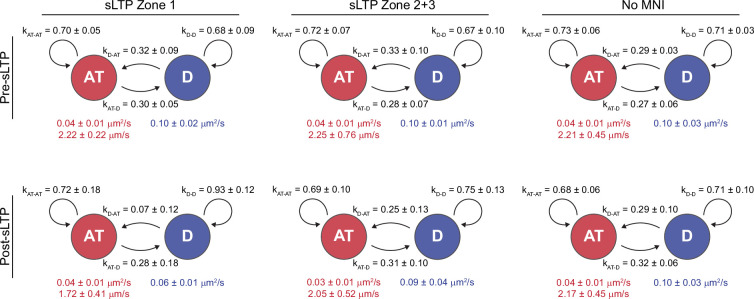
sLTP increases the probability GluA1-HT vesicles switch from active transport to diffusion proximal to the site of stimulation. Average state-switching probabilities for multi-state GluA1-HT vesicles before and after sLTP stimulation in Zone 1 (Zone 1), Zone 2+3 (Zone 2+3), and in Zone 1 in the absence of MNI (No MNI). k_AT-AT_, probability that a GluA1-HT vesicle remains in active transport (AT); k_AT-D_, probability that a vesicle switch from active transport to diffusion (D); k_D-AT_, probability that a vesicle switch from diffusion to active transport; k_D-D_, probability that a vesicle remains in diffusion. Values in red are the velocity and diffusion coefficient of the vesicle during active transport while the value in blue is the diffusion coefficient of the vesicle during diffusion. sLTP Zone 1: pre-sLTP, k_AT-D_ vs k_D-AT_, p=0.7104; post-sLTP, k_AT-D_ vs k_D-AT_, *p=0.0195. sLTP Zone 2+3: pre-sLTP, k_AT-D_ vs k_D-AT_, p=0.6200; post-sLTP, k_AT-D_ vs k_D-AT_, p=0.5887. No MNI: pre-sLTP, k_AT-D_ vs k_D-AT_, p=0.3829; post-sLTP, k_AT-D_ vs k_D-AT_, p=0.8182. Significance was determined by Mann-Whitney test. n=6–7 multi-state GluA1-HT vesicle trajectories for each condition. Figure 2—figure supplement 4—source data 1.Related to Figure 2—figure supplement 4.

**Figure 2—video 1.** Timelapse sequences of GluA1-HT-JF_549_ after block-and-chase labeling with JF_646_-HTL and JF_549_-HTL pre-sLTP (Figure 2—video 1, Pre-sLTP, left) and post-sLTP (Figure 2—video 1, Post-sLTP, right). Scale bar, 5 μm. Yellow dot indicates site of MNI uncaging.

To examine GluA1-HT vesicle motion proximal to the site of structural plasticity, we separated the dendritic shaft longitudinally (i.e. along the length of the dendrite) into three equal zones based on proximity to the site of uncaging and assessed the different types of motion that GluA1-HT vesicles exhibit in each zone after sLTP (Figure 2B and Figure 2—video 1). We find that sLTP results in reduced active transport and lower rates of diffusion for GluA1-HT vesicles in Zone 1 but not Zone 2 or Zone 3 (Figure 2C–D), indicating that stimulation disrupts GluA1-HT vesicle motion proximal, but not distal, to the site of synaptic activity. By contrast, sLTP stimulation in the absence of MNI failed to alter the fraction of vesicles exhibiting active transport or the diffusion coefficient of vesicles both proximal and distal to the site of stimulation (Figure 2—figure supplement 3B–C), demonstrating that changes in vesicle movement after sLTP are not the result of laser exposure. Furthermore, GluA1-HT vesicles in Zone 1 imaged 10 min after the cessation of sLTP stimulation exhibited similar motion behaviors to those imaged immediately after the cessation of sLTP (Figure 2—figure supplement 3A, Active transport bar graph and Diffusion histogram), suggesting that vesicles are confined.

To further test whether vesicles are confined near the site of synaptic activity, we used HMM-Bayes to determine the probabilities that vesicles switch between active transport and diffusion in each zone during sLTP. sLTP increases the probability that multi-state GluA1-HT vesicles undergoing active transport in Zone 1 switch to and stay in a diffusive state (Figure 2—figure supplement 4, sLTP Zone 1, k_D-AT_ vs k_AT-D_, and k_D-D_) in a manner dependent on the presence of MNI (Figure 2—figure supplement 4, No MNI, k_D-AT_ vs k_AT-D_, and k_D-D_). By contrast, multi-state vesicles in Zone 2 and Zone 3 have similar probabilities of switching between active transport and diffusion after sLTP (Figure 2—figure supplement 4, sLTP Zone 2+3, k_D-AT_ vs k_AT-D_). These observations demonstrate that multi-state GluA1-HT vesicles proximal to the site of stimulation have low probabilities of being transported away from the site of stimulation. Combined, these findings demonstrate that sLTP results in the confinement of GluA1-HT vesicles near the site of structural plasticity.

### Confinement of GluA1 vesicles during synaptic activity is mediated by F-actin-induced molecular crowding in the dendritic shaft

Having demonstrated that sLTP results in the confinement of GluA1-HT vesicles in the dendritic shaft near the site of structural plasticity, we sought to determine the molecular mechanisms involved in disrupting vesicle motion. We hypothesized that F-actin in the dendritic shaft might be involved because stimulating neuronal activity leads to elevated intracellular calcium levels and the activation of calcium signaling pathways that trigger the polymerization of actin (Okamoto et al., 2004; Okamoto et al., 2007; Okamoto et al., 2009). Moreover, recent studies have reported the rearrangement of F-actin networks in the dendritic shaft during neuronal activity (Schätzle et al., 2018; Lavoie-Cardinal et al., 2020), and found that F-actin networks can reposition lysosomes in neurites (Katrukha et al., 2017; van Bommel et al., 2019). Importantly, F-actin networks formed in response to sLTP are persistent (Okamoto et al., 2004), and therefore could be a mechanism to confine GluA1-HT vesicles even after the cessation of stimulation.

**Figure 3.**
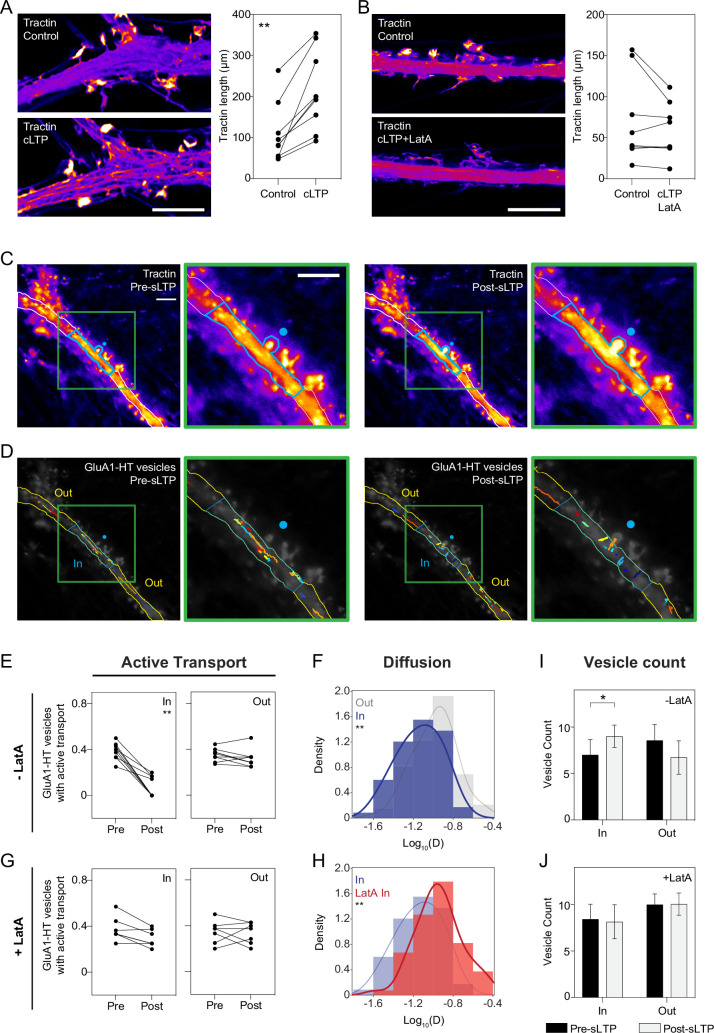
Reduced motion of GluA1-HT vesicles during synaptic activity is mediated by actin polymerization in the dendritic shaft. (**A**) Images: representative Airyscan images of F-tractin-mNeongreen (Tractin) in a dendrite before treatment (Control) and during cLTP (cLTP). Scale bar, 5 μm. Graph: combined length of tractin filaments (Tractin length) before treatment and during cLTP. **p=0.0039 by Wilcoxon matched pairs test. Each dot represents the tractin length in an imaged region of a dendrite. n=9 dendrite regions (each from one neuronal culture). (**B**) Same as (**A**), but in the presence of Latrunculin A (LatA). Scale bar, 5 μm. n=8 dendrite regions. (**C**) Representative epifluorescence images of tractin immediately before sLTP (Pre-sLTP) and after sLTP (Post-sLTP). Blue dot represents the site of uncaging. Blue outline denotes area with increased tractin signal after sLTP stimulation. Green inset: magnified image of tractin around the uncaging site. Scale bars, 10 μm. (**D**) GluA1-HT vesicle trajectories immediately before sLTP (Pre-sLTP) and after sLTP (Post-sLTP) inside (IN) and outside (OUT) the region where there was increased actin polymerization after sLTP stimulation. Green inset: magnified image of trajectories around the uncaging site. For video, see Figure 3—video 1. (**E**) Line graphs of fractions of GluA1-HT vesicles exhibiting active transport inside (IN) and outside (OUT) regions of actin polymerization pre- and post-sLTP. **p=0.0039 by Wilcoxon matched pairs test. Each dot represents the fraction of vesicles exhibiting active transport inside or outside the region with actin polymerization for a single sLTP stimulation experiment. n=9 sLTP stimulation experiments (each experiment targets one spine on one neuron) where GluA1-HT vesicle motion in the dendrite is captured immediately before and after sLTP stimulation. (**F**) Distributions of diffusion coefficients for GluA1-HT vesicles in regions with actin polymerization (IN; blue) versus regions without actin polymerization (OUT; gray) after sLTP stimulation. Lines represent the probability density function of each histogram estimated by kernel density estimation (KDE). **p=0.0053 by Kolmogorov-Smirnov test. n=59–73 GluA1-HT vesicle trajectories pooled together from nine sLTP stimulation experiments. (**G**) Same as (**E**), but in the presence of LatA. IN region defined as the 30 μm region flanking the uncaging site (the average length of dendrite where actin polymerization occurs after sLTP; Figure 3—figure supplement 3A), as we do not detect actin polymerization in the presence of LatA. n=7 sLTP stimulation experiments. (**H**) Distributions of diffusion coefficients for GluA1-HT vesicles in regions with actin polymerization (IN; blue) versus GluA1-HT vesicles in similar sized regions in the presence of LatA (LatA IN; red). **p=0.0013 by Kolmogorov-Smirnov test. n=59–67 GluA1-HT vesicle trajectories pooled together from seven to nine sLTP stimulation experiments for each condition. (**I–J**) Bar graphs of adjusted vesicle counts inside (IN) or outside (OUT) regions with actin polymerization after sLTP (**I**) or sLTP in the presence of LatA (**J**). Error bars represent standard deviation. *p=0.0111 by Mann-Whitney test. n=7–9 sLTP stimulation experiments for each condition. Figure 3—source data 1.Related to Figure 3.

To determine whether actin polymerization occurs in the dendritic shaft of cultured rat hippocampal neurons during synaptic activity, we tested whether cLTP would lead to the redistribution of F-tractin (tractin), an actin binding peptide that is used as a marker for F-actin (Schell et al., 2001). Prior to cLTP, tractin is diffusely distributed in the dendritic shaft and concentrated in spines (Figure 3A, Control). During cLTP induction, tractin in the dendritic shaft redistributes into a network of filaments (Figure 3A, cLTP). The combined length of these tractin filaments in the dendritic shaft is significantly greater during cLTP (Figure 3A, Tractin length, and Figure 3—figure supplement 1), suggesting that cLTP stimulates actin polymerization in the dendritic shaft. To eliminate the possibility that changes in the distribution of tractin are due to morphological changes in the dendrite, neurons were also transduced with a plasmid expressing tdTomato, which did not dramatically redistribute during cLTP (Figure 3—figure supplement 2A).

**Figure 3—figure supplement 1.**
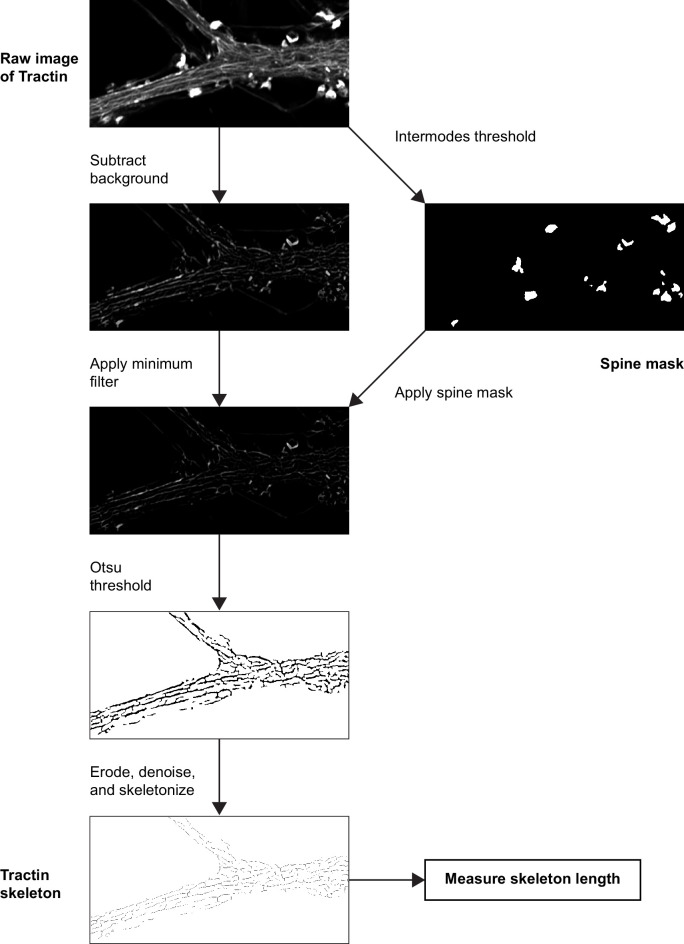
Measuring the length of tractin filaments. To measure the length of tractin filaments, we first generated a mask for spines by thresholding the brightest signals in an image (as F-actin is concentrated in spines, tractin signal is typically strongest in spines) using the Intermodes method, an automatic intensity thresholding method. Next, we applied rolling ball background subtraction and a minimum filter to improve the signal-to-noise ratio of tractin filaments. We then applied the spine mask to remove tractin signals from spines. We used Otsu’s method to threshold the resulting image and generate a binary mask of the tractin filaments. We then eroded and denoised the image to remove punctate signal and reduce thick signals that cannot be properly skeletonized. Finally, we skeletonized this binary mask and used the AnalyzeSkeleton ImageJ plugin (Arganda-Carreras et al., 2010) to measure the length of filaments. Dendrites prior to cLTP stimulation often did not have obvious tractin filaments. Consequently, we first processed post-cLTP images, and then applied thresholding values used for the post-cLTP images (identified using Otsu’s method) to the pre-cLTP image.

**Figure 3—figure supplement 2.**
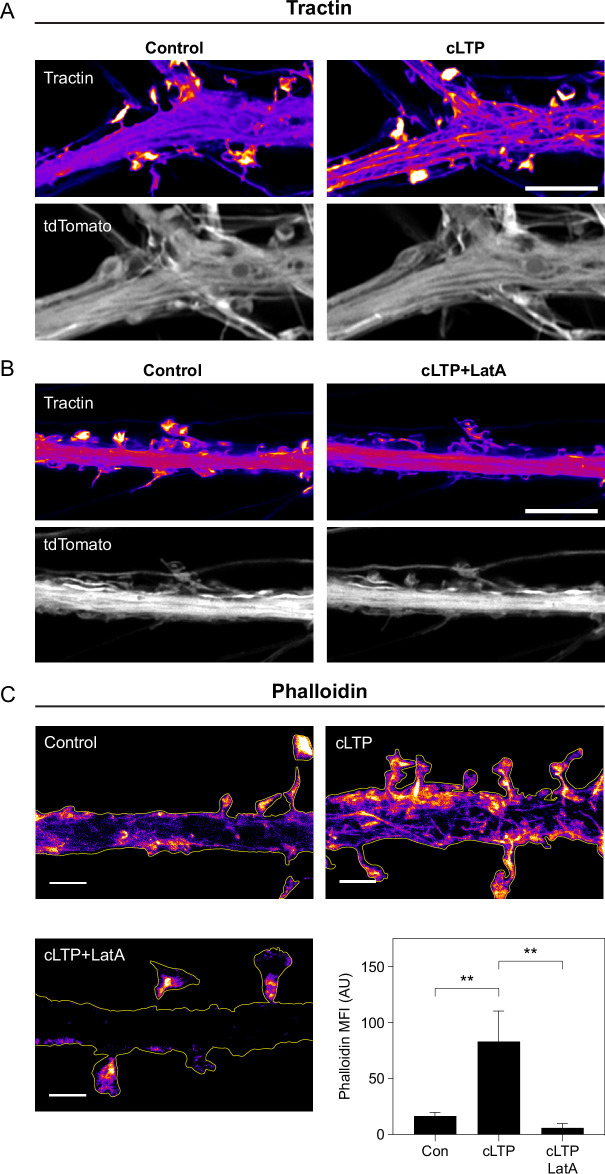
cLTP stimulation leads to increased actin polymerization. (**A**) Representative Airyscan images of tractin and tdTomato in a dendrite under control conditions and during cLTP. Scale bar, 5 μm. (**B**) Representative Airyscan images of tractin and tdTomato in a dendrite under control conditions and during cLTP in the presence of Latrunculin A (LatA). Scale bar, 5 μm. (**C**) Images: representative STED images of phalloidin staining in dendrites without treatment (Control), during cLTP (cLTP), and during cLTP in presence of LatA (cLTP +LatA). Scale bars, 2 μm. Graph: mean fluorescence intensity (MFI) of phalloidin staining. Bars represent mean and standard deviation. Con vs cLTP, **p=0.0079; cLTP vs cLTP +LatA, **p=0.0079. Significance was determined by Mann-Whitney test. n=5 phalloidin images (each image is of one region of dendrite from one neuron) for each condition. Figure 3—figure supplement 2—source data 1.Related to Figure 3—figure supplement 2.

**Figure 3—figure supplement 3.**
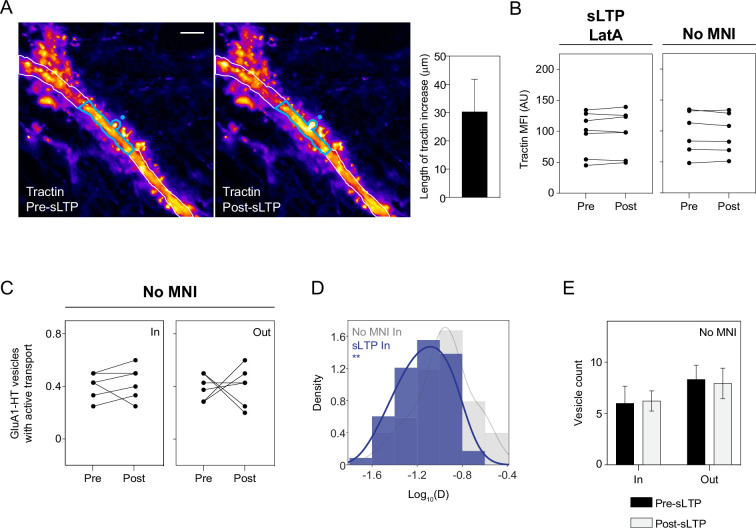
sLTP stimulation increases actin polymerization in the dendritic shaft proximal to the site of stimulation. (**A**) Images: representative epifluorescence images of tractin immediately before sLTP (Pre-sLTP) and after sLTP (Post-sLTP). Blue dot indicates the site of uncaging. Blue outline denotes area with increased tractin signal after stimulation. Scale bar, 10 μm. Bar graph: mean length (along the length of the dendrite) of the regions proximal to stimulated spines where increased tractin MFI was observed after sLTP (i.e. length of the blue outline). Error bar represents standard deviation. n=9 sLTP stimulation experiments (each experiment targets one spine on one neuron). (**B**) Mean fluorescence intensity (MFI) of tractin in dendrites pre- and post-sLTP in the presence of LatA (sLTP +LatA) and sLTP stimulation in the absence of MNI (No MNI). Tractin intensity was measured across the entire length of the dendrite because there were no specific regions where an increase in tractin intensity could be observed. n=6–7 sLTP stimulation experiments for each condition. (**C**) Line graphs of fractions of GluA1-HT vesicles exhibiting active transport before and after sLTP stimulation in the absence of MNI (No MNI). Because we did not detect significant increases in actin polymerization for No MNI, IN regions were determined by isolating the 30 μm region flanking the uncaging site, which was the mean length of the region where actin polymerization occurred after sLTP. n=6 sLTP stimulation experiments where GluA1-HT vesicle motion in the dendrite is captured immediately before and after sLTP stimulation. (**D**) Distributions of diffusion coefficients for GluA1-HT vesicles in regions with actin polymerization after sLTP (sLTP IN; blue) versus in equivalent regions after stimulation in the absence of MNI (No MNI IN; gray). Lines represent the probability density function of each histogram estimated by kernel density estimation (KDE). **p=0.0064 by Kolmogorov-Smirnov test. n=51–59 GluA1-HT vesicle trajectories pooled from six to nine sLTP stimulation experiments. (**E**) Bar graphs of adjusted vesicle counts after sLTP stimulation in the absence of MNI (No MNI). Error bars represent standard deviation. n=6 sLTP stimulation experiments. Figure 3—figure supplement 3—source data 1.Related to Figure 3—figure supplement 3.

**Figure 3—figure supplement 4.**
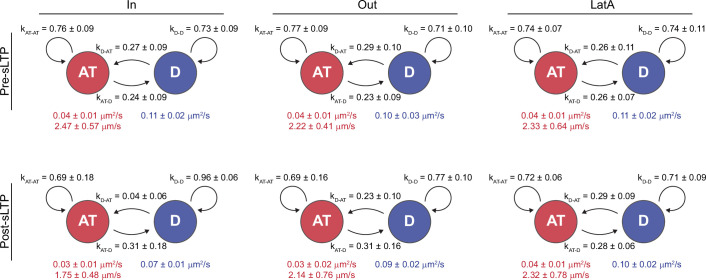
sLTP-induced actin polymerization increases the probability GluA1-HT vesicles switch from active transport to diffusion. Average state-switching probabilities for multi-state GluA1-HT vesicles before and after sLTP stimulation inside regions with actin polymerization (IN), outside regions with actin polymerization (OUT), and in the presence of LatA (LatA). k_AT-AT_, probability that a GluA1-HT vesicle remains in active transport (AT); k_AT-D_, probability that a vesicle switch from active transport to diffusion (D); k_D-AT_, probability that a vesicle switch from diffusion to active transport; k_D-D_, probability that a vesicle remains in diffusion. Values in red are the velocity and diffusion coefficient of the vesicle during active transport while the value in blue is the diffusion coefficient of the vesicle during diffusion. IN: pre-sLTP, k_AT-D_ vs k_D-AT_, p=0.4848; post-sLTP, k_AT-D_ vs k_D-AT_, *p=0.0317. OUT: pre-sLTP, k_AT-D_ vs k_D-AT_, p=0.3823; post-sLTP, k_AT-D_ vs k_D-AT_, p=0.4848. LatA: pre-sLTP, k_AT-D_ vs k_D-AT_, p=0.9015; post-sLTP, k_AT-D_ vs k_D-AT_, p>0.9999. Significance was determined by Mann-Whitney test. n=5–8 multi-state GluA1-HT vesicle trajectories for each condition. Figure 3—figure supplement 4—source data 1.Related to Figure 3—figure supplement 4.

**Figure 3—figure supplement 5.**
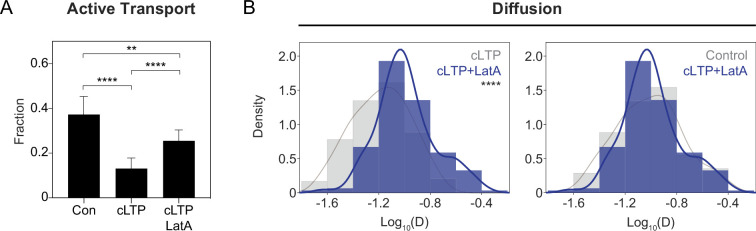
Blocking actin polymerization with LatA disrupts the effect of cLTP on the motion of GluA1-HT vesicles. (**A**) Fraction of GluA1-HT vesicles exhibiting active transport in dendritic shafts without treatment (Con), during cLTP (cLTP), and during cLTP in the presence of LatA (cLTP +LatA). Con vs cLTP, ****p<0.0001; Con vs cLTP +LatA, **p=0.0010; cLTP vs cLTP +LatA, ****p<0.0001. Significance was determined by Mann-Whitney test. n=9–14 timelapse imaging sequences (each timelapse captures the motion of GluA1-HT vesicles in one region of dendrite in one neuronal culture) for each condition. (**B**) Histograms: distributions of diffusion coefficients for GluA1-HT vesicles in dendritic shafts during cLTP +LatA (blue) versus cLTP (gray; left) or Control (gray; right). Line represents the probability density function of each histogram estimated by kernel density estimation (KDE). cLTP vs cLTP +LatA, ****p<0.0001 by Kolmogorov-Smirnov test. n=227–360 GluA1-HT vesicle trajectories pooled from 9 to 14 timelapse imaging sequences. Figure 3—figure supplement 5—source data 1.Related to Figure 3—figure supplement 5.

**Figure 3—figure supplement 6.**
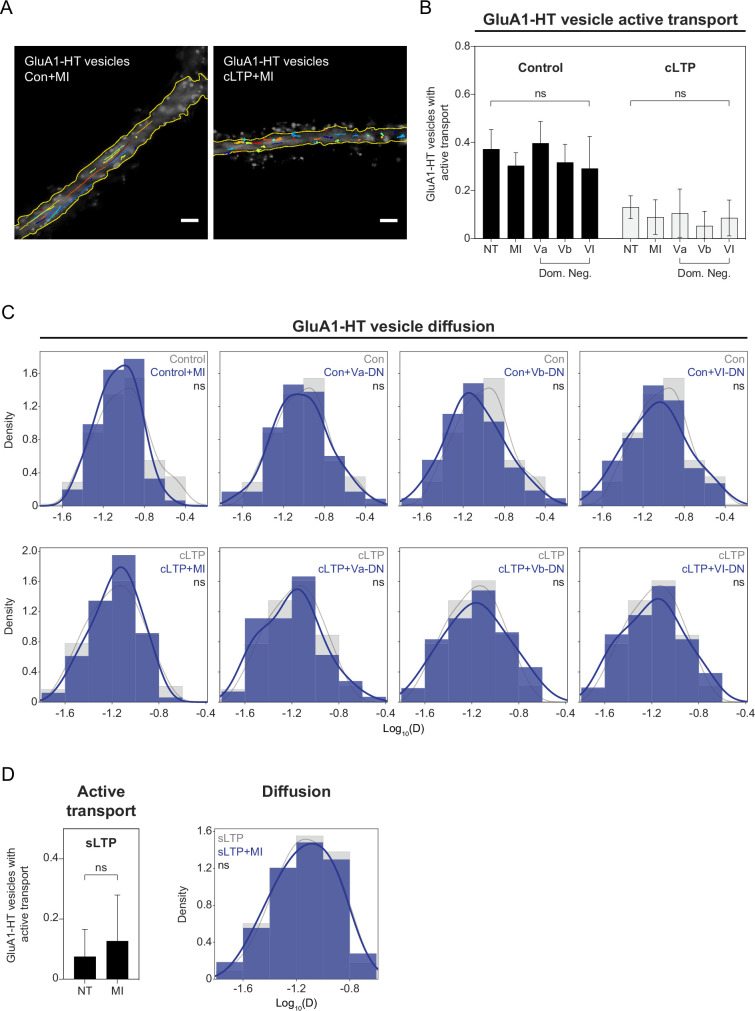
Inhibition of myosin V and VI does not affect the motion states of GluA1-HT vesicles. (**A**) GluA1-HT vesicle trajectories in the presence of a myosin V and VI inhibitor cocktail (MI) without stimulation (Con +MI) or during cLTP induction (cLTP +MI). Trajectories are overlaid onto images of GFP-Homer1c. Scale bars, 5 μm. For videos, see Figure 3—video 2 and Figure 3—video 3. (**B**) Fractions of GluA1-HT vesicles exhibiting active transport in the presence of inhibitory C-terminal domains of Myosin Va, Vb, or VI (dominant-negative), or pharmacological inhibition (MI), without stimulation or during cLTP. NT, not treated with inhibitors or dominant-negative C-terminal domains. Bars represent mean and standard deviation. Significance was determined by Mann-Whitney test. n=3–14 timelapse imaging sequences (each timelapse captures the motion of GluA1-HT vesicles in one region of dendrite in one neuronal culture) for each condition. (**C**) Distributions of diffusion coefficients of GluA1-HT vesicles in dendritic shafts in the presence of inhibitory C-terminal domains of Myosin Va, Vb, or VI (dominant-negative; blue), or pharmacological inhibition (MI; blue), without stimulation (top row) or during cLTP (bottom row). Lines represent the probability density function of each histogram estimated by kernel density estimation (KDE). n=54–360 GluA1-HT vesicle trajectories pooled from 3 to 14 timelapse imaging sequences for each condition. (**D**) Left bar graph: fractions of GluA1-HT vesicles exhibiting active transport in the presence of a myosin inhibitor cocktail (MI) without stimulation or after sLTP. NT, not treated with inhibitors. Bars represent mean and standard deviation. Significance was determined by Mann-Whitney test. n=6–9 sLTP stimulation experiments (each experiment targets one spine on one neuron) where GluA1-HT vesicle motion in the dendrite is captured immediately after sLTP stimulation. Right histogram: distribution of diffusion coefficients of GluA1-HT vesicles after sLTP stimulation in dendritic shafts in the presence of a myosin inhibitor cocktail (MI; blue). Lines represent the probability density function of each histogram estimated by kernel density estimation (KDE). Significance was determined by Kolmogorov-Smirnov test. n=54–59 GluA1-HT vesicle trajectories pooled from six to nine sLTP stimulation experiments for each condition. Figure 3—figure supplement 6—source data 1.Related to Figure 3—figure supplement 6.

**Figure 3—video 1.** Timelapse sequences of GluA1-HT-JF_549_ after block-and-chase labeling with JF_646_-HTL and JF_549_-HTL pre-sLTP (Figure 3—video 1, Pre-sLTP, left) and post-sLTP (Figure 3—video 1, Post-sLTP, right). Scale bar, 5 μm. Yellow dot indicates site of MNI uncaging. Performed in neuron also expressing tractin to characterize the motion of GluA1-HT vesicles in areas where actin polymerization occurs.

**Figure 3—video 2.** Timelapse sequence of GluA1-HT-JF_549_ after block-and-chase labeling with JF_646_-HTL and JF_549_-HTL in a dendrite that has been treated with a myosin inhibitor cocktail with no cLTP stimulation (Con +MI). Scale bar, 5 μm. Left: GFP-Homer1c. Center: GluA1-HT-JF_549_ (chase). Right: GluA1-HT-JF_549_ (green) overlaid onto GFP-Homer1c (magenta). Related to Figure 3—figure supplement 6.

**Figure 3—video 3.** Timelapse sequence of GluA1-HT-JF_549_ after block-and-chase labeling with JF_646_-HTL and JF_549_-HTL in a dendrite that has been treated with a myosin inhibitor cocktail during cLTP induction (cLTP +MI). Scale bar, 5 μm. Left: GFP-Homer1c. Center: GluA1-HT-JF_549_ (chase). Right: GluA1-HT-JF_549_ (green) overlaid onto GFP-Homer1c (magenta). Related to Figure 3—figure supplement 6.

To confirm that the change in tractin distribution during cLTP induction is the result of actin polymerization, we imaged tractin during cLTP in dendrites that were also treated with Latrunculin A (LatA), an inhibitor of actin polymerization (Figure 3B and Figure 3—figure supplement 2B). Treating neurons with LatA not only prevents redistribution of tractin during cLTP, but also reduces the intensity of tractin signal in dendritic spines (Figure 3B, cLTP +LatA), demonstrating that actin polymerization leads to the redistribution of tractin during cLTP. Because the expression of actin-binding peptides may result in artificial F-actin structures (Melak et al., 2017), we also labeled F-actin with phalloidin after cLTP and imaged using STED microscopy. cLTP resulted in greater phalloidin labeling in dendritic shafts, while LatA treatment during cLTP decreased phalloidin labeling (Figure 3—figure supplement 2C), recapitulating our finding that cLTP induces actin polymerization in the dendritic shaft.

We next sought to determine whether sLTP-mediated changes in local actin networks play a role in positioning vesicles near sites of stimulation. We observed a significant increase in tractin fluorescence (MFI) in spines stimulated with sLTP and in the dendritic shaft proximal to these spines, reflecting increased actin polymerization at these locations (Figure 3C, blue outline). Tractin signal increases in an approximately 30 μm longitudinal section along the length of the dendritic shaft surrounding the sLTP-stimulated spine (Figure 3—figure supplement 3A). The increase in tractin MFI during sLTP is dependent on actin polymerization and is not an artifact of photostimulation (Figure 3—figure supplement 3B).

Having found that sLTP increases actin polymerization in the dendritic shaft proximal to the uncaging site, we tested whether sLTP-mediated actin polymerization confined GluA1-HT vesicles. By tracking GluA1-HT vesicles after sLTP (Figure 3D and Figure 3—video 1), we find that sLTP significantly reduces the fraction of GluA1-HT vesicles undergoing active transport, as well as the diffusion coefficient of GluA1-HT vesicles, inside but not outside regions of the dendritic shaft with actin polymerization (Figure 3E–F). Moreover, multi-state GluA1-HT vesicles undergoing active transport inside, but not outside, regions of actin polymerization during sLTP have an increased probability of switching to diffusion (Figure 3—figure supplement 4). By contrast, sLTP stimulation has no effect on the motion of vesicles in cultures with no MNI (Figure 3—figure supplement 3C–D). Importantly, the effect of sLTP on GluA1-HT vesicle motion is disrupted by LatA treatment (Figure 3G–H and Figure 3—figure supplement 4). Similarly, LatA prevented cLTP-mediated changes in GluA1-HT vesicle mobility, demonstrating that cLTP-induced actin polymerization results in the confinement of GluA1-HT vesicles as well (Figure 3—figure supplement 5 and Figure 1—figure supplement 11).

These results demonstrate that sLTP-mediated actin polymerization in the dendritic shaft confines GluA1-HT vesicles near the site of stimulation, but it is unclear whether confinement actually generates an increased number of vesicles near these sites. After adjusting the number of vesicles for photobleaching (Materials and methods), we find a significant increase in the number of GluA1-HT vesicles inside, but not outside, regions of actin polymerization after sLTP (Figure 3I and Figure 3—figure supplement 3E). Moreover, the increase in GluA1-HT vesicles is blocked by the addition of LatA (Figure 3J). Combined, these observations demonstrate that neurons use actin polymerization as a mechanism to confine and increase the number of vesicles near the sites of synaptic activity.

Having established actin polymerization as the mechanism that mediates stimulation-dependent GluA1-HT vesicle confinement, we sought to determine the mechanism by which F-actin perturbed vesicle motion. AMPARs interact with myosins Va, Vb, and VI (Correia et al., 2008; Wang et al., 2008; Nash et al., 2010; EstevesdaSilva et al., 2015), which are involved in the calcium-dependent, short-range recruitment of AMPARs in endosomes to and from dendritic spines. To test if interactions between GluA1 and myosin V and/or VI anchor GluA1 vesicles to F-actin during neuronal activity, we inhibited myosin Va, Vb, and VI by expressing dominant-negative c-terminal domains of these proteins, and by using a pharmacological inhibitor cocktail (MI), during cLTP stimulation (Figure 3—figure supplement 6A–C, Figure 3—video 2 and Figure 3—video 3). Inhibition of myosin did not alter the fractions of GluA1-HT vesicles exhibiting active transport, or the diffusion coefficients of GluA1-HT vesicles, either under unstimulated conditions or during cLTP (Figure 3—figure supplement 6B–C). Likewise, acute pharmacological inhibition of myosin did not affect the fraction of GluA1-HT vesicles exhibiting active transport, or the diffusion coefficient of GluA1-HT vesicles, after sLTP stimulation (Figure 3—figure supplement 6D). Based on these observations, we conclude that F-actin disrupts GluA1-HT vesicle motion in a manner independent of myosin activity.

**Figure 4.**
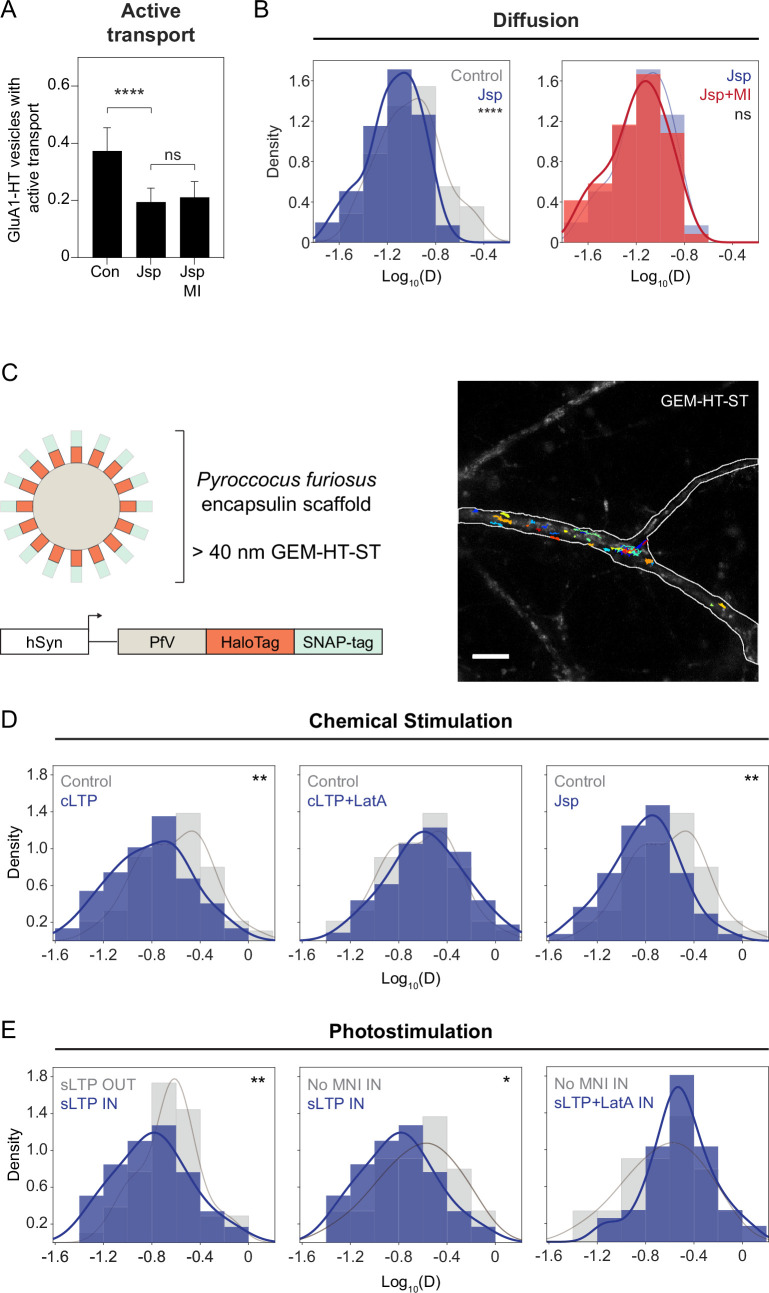
F-actin-induced molecular crowding inhibits the motion of particles near the sites of synaptic activity. (**A**) Fractions of GluA1-HT vesicles exhibiting active transport in dendritic shafts without treatment (Con) versus during treatment with Jasplakinolide (Jsp) or Jsp with pharmacological inhibition of myosin (Jsp +MI). Bars represent mean and standard deviation. Con vs Jsp, ****p<0.0001. Significance was determined by Mann-Whitney test. n=9–12 timelapse imaging sequences (each timelapse captures the motion of GluA1-HT vesicles in one region of dendrite in one neuronal culture) for each condition. (**B**) Left: distributions of diffusion coefficients of GluA1-HT vesicles in dendritic shafts without treatment (Control; gray) versus during treatment with Jsp (Jsp; blue). Lines represent the probability density function of each histogram estimated by kernel density estimation (KDE). Control vs Jsp, ****p<0.0001 by Kolmogorov-Smirnov test. Right: distributions of diffusion coefficients of GluA1-HT vesicles in dendritic shafts during treatment with Jsp (Jsp; blue) versus Jsp with pharmacological inhibition of myosin (Jsp +MI; red). n=60–227 GluA1-HT vesicle trajectories pooled from 9 to 12 timelapse imaging sequences for each condition. (**C**) Schematic: GEM-HT-ST rheological probe (top). GEM-HT-ST was created by fusing HaloTag (HT) and SNAP-tag (ST) to the PfV protein from *Pyroccocus furiosus* (bottom; Delarue et al., 2018). When expressed, PfV-HT-ST fusion proteins self-assemble into a 40 nm encapsulin scaffold that can be labeled with either JF_549_-HTL or JF_549_-SNAP-tag ligand (JF_549_-STL). Image: trajectories of GEM-HT-ST labeled with JF_549_-HTL. Scale bar, 10 μm. For video, see Figure 4—video 1. (**D**) Distributions of diffusion coefficients of GEM-HT-ST after chemical stimulation. Control (gray) vs cLTP (blue), **p=0.0047; Control (gray) vs Jsp (blue), **p=0.0011. Significance was determined by Kolmogorov-Smirnov test. n=57–94 GEM-HT-ST trajectories pooled together from six to nine timelapse imaging sequences for each condition. (**E**) Distributions of diffusion coefficients of GEM-HT-ST after sLTP in regions where actin polymerization occurred versus controls. IN region was determined by isolating a 30 μm region flanking the uncaging site (the average length of dendrite where actin polymerization occurs after sLTP; Figure 3—figure supplement 3A). sLTP IN (blue) vs sLTP OUT (gray), **p=0.0083; sLTP IN (blue) vs No MNI IN (gray), *p=0.0446. n=44–59 GEM-HT-ST trajectories pooled together from six to nine sLTP stimulation experiments (each experiment targets one spine on one neuron) where GEM-HT-ST motion in the dendrite is captured immediately after sLTP stimulation. Figure 4—source data 1.Related to Figure 4.

Previous studies have demonstrated that F-actin can constrain the motion of lysosomes (van Bommel et al., 2019), leading us to speculate that actin polymerization itself could block the motion of GluA1-HT vesicles. Treatment of neurons with Jasplakinolide (Jsp), an F-actin stabilizer that promotes actin polymerization, is sufficient to inhibit active transport and reduce the rate of diffusion of GluA1-HT vesicles (Figure 4A and Figure 4B, left histogram). Furthermore, the Jsp-mediated reduction in GluA1-HT vesicle motion is not altered by pharmacological inhibition of myosin, indicating that F-actin itself plays a role in the reduced motion of GluA1-HT vesicles (Figure 4A and Figure 4B, right histogram).

**Figure 4—video 1.** Timelapse sequence of GEM-HT-ST labeled with JF_549_-HTL in the dendrite of a cultured rat hippocampal neuron under control conditions. Scale bar, 5 μm.

Previous studies have found that actin can induce molecular crowding in biological systems such as neuronal axons and prevent active transport of vesicles and organelles (Sood et al., 2018). To test if actin polymerization could disrupt vesicle motion by changing the rheological properties of the dendritic cytoplasm, we evaluated whether cLTP and sLTP can alter the diffusion of a rheological probe consisting of a genetically encoded multimeric nanoparticle (GEM; Delarue et al., 2018; Figure 4C). GEM is based on the encapsulin protein PfV of *Pyroccocus furiosus* (Figure 4C, schematic), which self-assembles into an icosahedral scaffold of 120 monomers whose size is more similar to GluA1-HT vesicles than are soluble fluorescent proteins. When fused to both HaloTag and the self-labeling SNAP-tag (ST), the particle arising from expression of GEM-HT-ST can be labeled with Janelia Fluor dye ligands and tracked in dendrites (Figure 4C, image, and Figure 4—video 1).

We find that GEM-HT-ST diffusion is significantly reduced during cLTP induction (Figure 4D, cLTP), and this reduction is dependent on actin polymerization as LatA prevents the decrease in diffusion coefficient (Figure 4D, cLTP +LatA). Moreover, actin polymerization stimulated by Jsp is sufficient to reduce the motion of GEM-HT-ST (Figure 4D, Jsp). sLTP also results in a reduction in the rate of GEM-HT-ST diffusion in regions of dendritic shafts where actin polymerization occurs (Figure 4E, sLTP IN vs sLTP OUT) in a manner that is dependent on the presence of MNI (Figure 4E, No MNI IN) and actin polymerization (Figure 4E, sLTP +LatA IN). These experiments demonstrate that synaptic activity changes the rheological properties of the dendritic shaft by stimulating actin polymerization. Combined with our findings that triggering actin polymerization is sufficient to disrupt GluA1-HT vesicle motion (via Jsp treatment) and that myosin inhibition did not alter activity-mediated changes in motion, these observations are consistent with the hypothesis that actin polymerization confines GluA1-HT vesicles by altering the properties of the dendritic cytoplasm independent of direct interactions between GluA1 and myosin.

### Local increase in GluA1 exocytosis triggered by synaptic activity is dependent on actin-mediated GluA1 vesicle confinement and myosin activity

**Figure 5.**
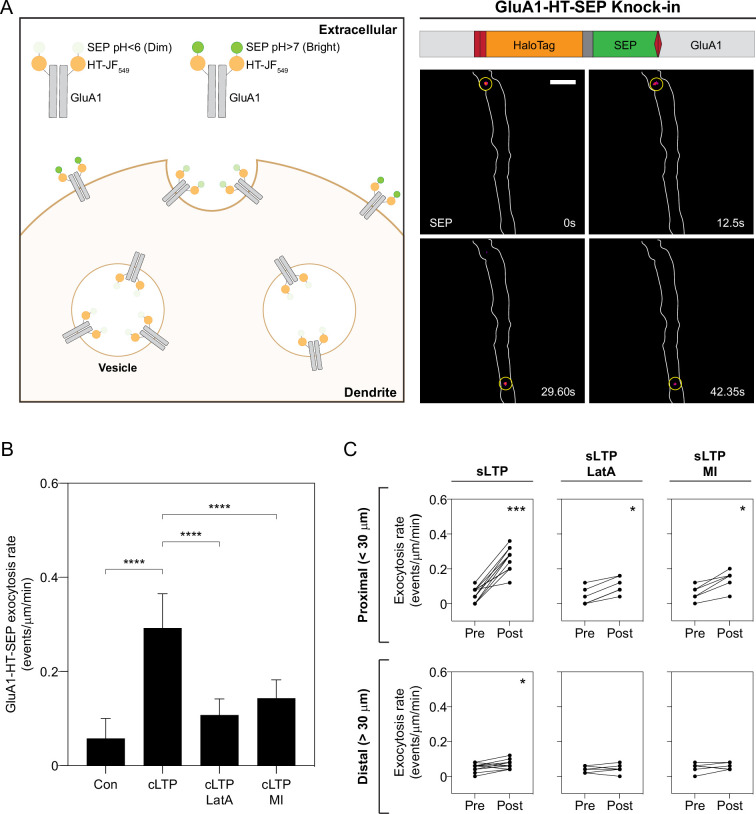
Increased GluA1-HT-SEP exocytosis triggered by synaptic activity is dependent on actin polymerization and myosin activity. (**A**) Tagging endogenous GluA1 with a HaloTag-pHluorin tandem fusion reporter (HT-SEP) to track GluA1 exocytosis events. Diagram: endogenous GluA1 tagged with HT-SEP (GluA1-HT-SEP, schematic of fusion on right) has low SEP fluorescence inside vesicles due to the low pH of the vesicle lumen. When a GluA1 exocytosis event occurs, SEP will be exposed to the neutral pH of the extracellular medium, resulting in increased fluorescence. Because receptors are temporarily spatially restricted during exocytosis, a spot with fluorescence can be observed during the event. Images: timelapse of two GluA1-HT-SEP exocytosis events (yellow circles). Scale bar, 5 μm. For video, see Figure 5—video 1. (**B**) Bar graph of GluA1-HT-SEP exocytosis in response to cLTP in the absence or presence of Latrunculin A (LatA) or pharmacological myosin inhibition (MI). Bars represent mean events per μm per min and standard deviation. Con vs cLTP, ****p<0.0001; cLTP vs cLTP +LatA, ****p<0.0001; cLTP vs cLTP +MI, ****p<0.0001. Significance was determined by Mann-Whitney test. n=12 timelapse imaging sequences (each timelapse captures GluA1-HT-SEP exocytosis in one region of dendrite in one neuronal culture) for each condition. (**C**) Line graphs of GluA1-HT-SEP exocytic events before and after sLTP (sLTP), sLTP in the presence of LatA (sLTP +LatA), and sLTP in the presence of MI (sLTP +MI). Proximal: GluA1-HT-SEP exocytic events within the 30 μm region surrounding the site of uncaging (i.e. the average length of dendrite where actin polymerization occurs near the site of uncaging after sLTP; see Figure 3—figure supplement 3A). sLTP, ***p=0.0005; sLTP +LatA, *p=0.0312; sLTP +MI, *p=0.0312. Distal: GluA1-HT-SEP exocytic events outside of the 30 μm region surrounding the site of uncaging. sLTP, *p=0.0430. Significance was determined by Wilcoxon matched pairs test. Each dot represents the number of exocytic events in the indicated region. n=6–12 sLTP stimulation experiments (each experiment targets one spine on one neuron) where GluA1-HT-SEP exocytosis in the dendrite is captured immediately before and after sLTP stimulation. Figure 5—source data 1.Related to Figure 5.

Although we have shown that actin polymerization can concentrate GluA1 vesicles near the sites of synaptic activity, it is unclear whether this mechanism contributes to increased trafficking of GluA1 to synapses during activity – whether positioning vesicles near the sites of activity also results in increased exocytosis of GluA1-HT at these sites. To study the exocytic rates of endogenous GluA1, we fused super ecliptic pHluorin (SEP) to HaloTag and knocked this tandem reporter into the NTD of GluA1 such that the tag is exposed to the low pH lumen of vesicles during transport (Figure 5A and Figure 5—figure supplements 1–2). Intracellular GluA1-HT-SEP has low fluorescence until exocytosis, at which point it is exposed to the neutral pH extracellular medium and exhibits strong fluorescence (Figure 5A, diagram). During exocytosis, GluA1-HT-SEP released from vesicles is temporarily confined at the sites of exocytosis and appears as bright puncta under fluorescence microscopy (Figure 5A, images on right, and Figure 5—video 1). GluA1-HT-SEP exocytic events occur at a low rate prior to stimulation, but increase dramatically during cLTP induction (Figure 5B, cLTP, and Figure 5—video 2), consistent with previous findings (Kopec et al., 2006). The increase in GluA1-HT-SEP exocytosis is dependent on actin polymerization, as LatA reduces the rate of exocytosis during cLTP induction (Figure 5B, cLTP +LatA). Acute pharmacological inhibition of myosin also disrupts cLTP-stimulated GluA1-HT-SEP exocytosis (Figure 5B, cLTP +MI), suggesting that while myosin does not play a role in disrupting transport of GluA1-HT vesicles, it plays a role in regulating exocytosis of GluA1-HT-SEP. These observations demonstrate that actin polymerization and myosin mediate GluA1-HT-SEP exocytosis during cLTP.

**Figure 5—figure supplement 1.**
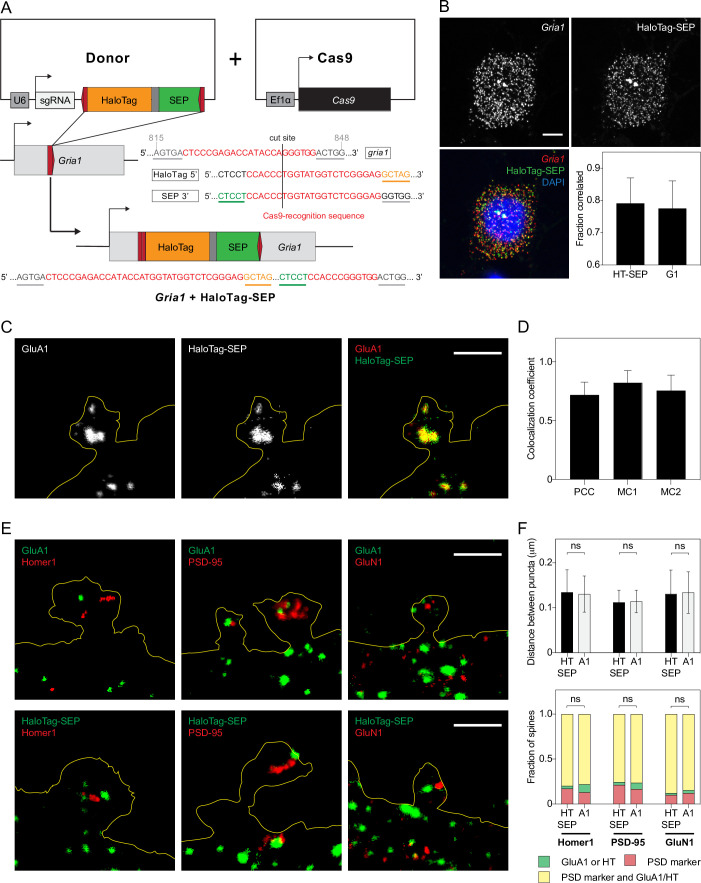
Endogenous GluA1 tagged with HaloTag-SEP reporter via HITI is trafficked to postsynaptic densities. (**A**) Schematic of *Gria1* gene targeting with HaloTag-SEP by HITI. (**B**) Images: representative confocal images of a two-color HCR RNA-FISH labeling experiment with probes targeting HaloTag-SEP mRNA (HaloTag-SEP) or *Gria1* mRNA (*Gria1*). Scale bar, 5 µm. Bar graph: fraction of HaloTag-SEP mRNA spots located within 0.5 μm of *Gria1* mRNA spots, and fraction of *Gria1* mRNA spots located within 0.5 μm of HaloTag-SEP mRNA spots. n=10 images (each image is of a neuron labeled with both probes for *Gria1* mRNA and HaloTag-SEP mRNA). Correlated localization was used to establish intramolecular labeling between two probe sets (Materials and methods). (**C**) Representative STED images of a dendritic spine expressing endogenous GluA1 tagged with HaloTag-SEP labeled with anti-GluA1 antibody (GluA1) and anti-HaloTag antibody (HaloTag-SEP). Scale bar, 2 μm. (**D**) Pearson correlation coefficients (PCC) and Manders’ overlap coefficients (MC1 and MC2) between HaloTag and GluA1 labeling. Bars represent mean and standard deviation. Significance was determined by Mann-Whitney test. n=8 images (each image is of a region of dendrite from one neuron labeled with anti-GluA1 and anti-HaloTag antibodies). (**E**) Representative STED images of dendritic spines labeled with anti-GluA1 (top) or anti-HaloTag (bottom) antibodies and anti-Homer1, anti-PSD-95, or anti-GluN1 antibodies. Scale bars, 2 μm. (**F**) Top: mean distances between the centers of HaloTag-SEP (HT-SEP) or GluA1 (A1) fluorescence puncta and Homer1, PSD-95, or GluN1 fluorescence puncta. Bars represent mean and standard deviation. n=50–145 unique pairs of puncta from seven to nine dendrite images for each labeling combination (e.g. HaloTag-SEP and Homer1). Bottom: fractions of spines with only GluA1 or HaloTag-SEP; only a postsynaptic density (PSD) marker; or both GluA1 or HaloTag-SEP and a PSD marker. n=50–110 spines from seven to nine dendrite images for each labeling combination. Significance was determined by Mann-Whitney test. Figure 5—figure supplement 1—source data 1.Related to Figure 5—figure supplement 1.

**Figure 5—figure supplement 2.**
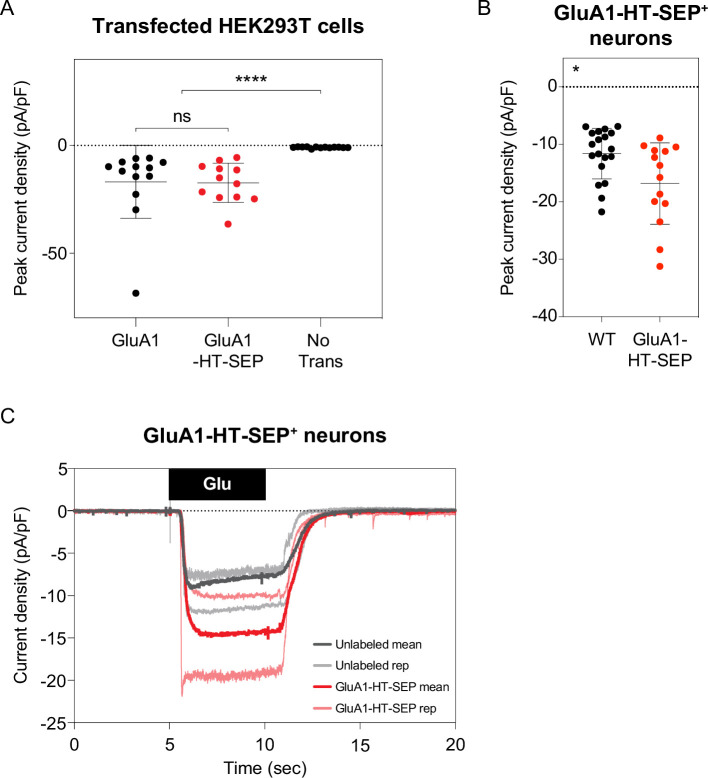
Currents elicited by GluA1-HT-SEP are similar to currents elicited by untagged GluA1. (**A**) Currents elicited by human embryonic kidney (HEK) 293T cells expressing GluA1 or GluA1-HT-SEP in response to locally perfused glutamate. Crosshairs represent mean and standard deviation of peak current densities (pA/pF) for each condition. Each dot represents a single recording. ****p<0.0001 by Mann-Whitney test. GluA1, n=13; GluA1-HT-SEP, n=12; No Transfection, n=13. Current densities for HEK293T cells were recorded following local application of 2 mM glutamate with 50 µM cyclothiazide for 10 s. (**B**) Currents elicited by GluA1-HT-SEP and unlabeled neurons in response to locally perfused glutamate. *p=0.0192 by Mann-Whitney test. GluA1-HT-SEP, n=14; unlabeled, n=19. Current densities for GluA1-HT-SEP neurons and unlabeled neurons were recorded following a 5 s 100 µM glutamate stimulation at a –70 mV holding potential. (**C**) Current density traces for GluA1-HT-SEP and unlabeled neurons in response to glutamate. Thin lines represent currents from two individual cells under each condition while thick lines represent mean currents. The small but notable reduction in mean trace peak amplitudes is a consequence of data decimation necessary for figure generation. GluA1-HT-SEP exhibits a modest but significant increase in current in edited neurons, possibly reflecting some disruptive properties of the HT-SEP tandem reporter. Nevertheless, GluA1-HT-SEP is trafficked to synapses in a similar manner to GluA1, and its function is not inhibited by the insertion of HT-SEP. Figure 5—figure supplement 2—source data 1.Related to Figure 5—figure supplement 2.

**Figure 5—figure supplement 3.**
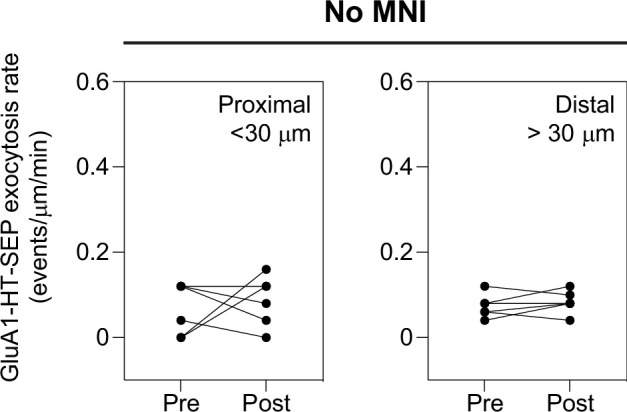
sLTP stimulation in the absence of MNI does not increase GluA1-HT-SEP exocytosis. Line graphs of GluA1-HT-SEP exocytic events before and after sLTP stimulation in the absence of MNI (No MNI). Proximal: GluA1-HT-SEP exocytic events within the 30 μm region surrounding the site of uncaging (i.e. the average length of dendrite where actin polymerization occurs near the site of uncaging during sLTP; see Figure 3—figure supplement 3A). Distal: GluA1-HT-SEP exocytic events outside of the 30 μm region surrounding the site of uncaging. n=6 sLTP stimulation experiments (each experiment targets one spine on one neuron) where GluA1-HT-SEP exocytosis in the dendrite is captured immediately before and after sLTP stimulation. Figure 5—figure supplement 3—source data 1.Related to Figure 5—figure supplement 3.

**Figure 5—video 1.** Timelapse sequence of GluA1-HT-SEP exocytosis in the dendrite of a cultured rat hippocampal neuron under control conditions. Scale bar, 5 μm. White circles denote sites of exocytosis.

**Figure 5—video 2.** Timelapse sequence of GluA1-HT-SEP exocytosis in a dendrite during cLTP induction. Scale bar, 5 μm. White circles denote sites of exocytosis.

**Figure 5—video 3.** Timelapse sequence of GluA1-HT-SEP exocytosis in a dendrite pre-sLTP (Figure 5—video 3, Pre-sLTP, left) and post-sLTP (Figure 5—video 3, Post-sLTP, right). Scale bar, 5 μm. Yellow dot indicates site of MNI uncaging. White circles denote sites of exocytosis outside the 30 μm region flanking the uncaging site (OUT). Green circles denote sites of exocytosis inside the 30 μm region flanking the uncaging site (IN).

We next tested whether sLTP results in local increases in GluA1-HT-SEP exocytosis, and if the increase in exocytosis is spatially correlated with, and dependent on, actin polymerization (Figure 5C and Figure 5—video 3). In the absence of a marker for F-actin (due to overlapping fluorescence signals between tractin and SEP), we defined the area proximal to the site of stimulation – the 30 µm region surrounding the uncaging site – as the region of actin polymerization based on our previous observations (Figure 3—figure supplement 3A). sLTP stimulation increases GluA1-HT-SEP exocytosis events to a much greater extent proximal than distal to the site of uncaging (Figure 5C, sLTP). Similar to their effect on cLTP-mediated GluA1-HT-SEP exocytosis, LatA treatment and acute myosin inhibition both partially block sLTP-mediated increases in GluA1-HT-SEP exocytosis (Figure 5C, sLTP +LatA and sLTP +MI). When MNI is removed from the media, there is no increase in exocytic events after sLTP (Figure 5—figure supplement 3). We conclude that the accumulation of GluA1-HT vesicles near sites of sLTP is spatially correlated with increased exocytosis of GluA1-HT-SEP, and that local disruption of GluA1-HT vesicle motion and the increase in GluA1-HT-SEP exocytosis are both dependent on actin polymerization in the dendritic shaft.

**Figure 6.**
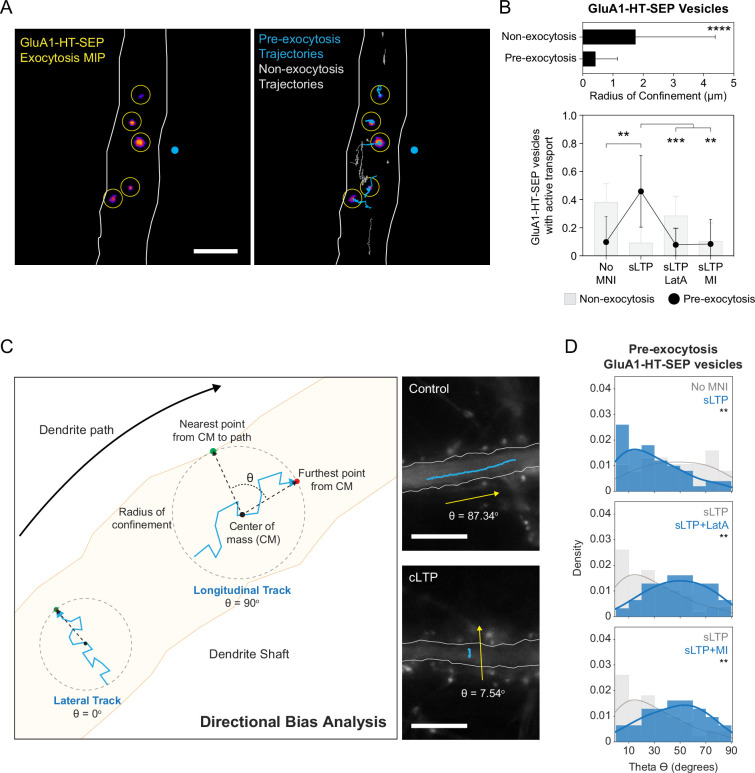
GluA1-HT-SEP vesicles are drawn from a local pool in the dendritic shaft prior to exocytosis and exhibit lateral motion that is dependent on actin polymerization and myosin in the shaft during synaptic activity. (**A**) Left: representative maximum intensity projection (MIP) of GluA1-HT-SEP exocytosis over time after sLTP (i.e. the brightest pixels from each image in a timelapse compressed into a single image). Yellow circles indicate exocytic events. Blue dot indicates site of uncaging. Right: GluA1-HT-SEP vesicle trajectories overlaid on the MIP of exocytosis over time. Blue trajectories are GluA1-HT-SEP vesicles that undergo exocytosis (Pre-exocytosis). Gray trajectories are GluA1-HT-SEP vesicles with no detected exocytosis (Non-exocytosis) during the span of imaging. Scale bar, 5 μm. For video. See Figure 6—video 1. (**B**) Top: mean radius of confinement for the trajectories of GluA1-HT-SEP vesicles that do not undergo exocytosis (Non-exocytosis) and that do undergo exocytosis (Pre-exocytosis) under unstimulated conditions. Error bars represent standard deviation. ****p<0.0001 by Kolmogorov-Smirnov test. n=65–176 GluA1-HT-SEP vesicle trajectories pooled from five timelapses imaging experiments (each timelapse captures GluA1-HT-SEP motion and exocytosis in one region of dendrite in one neuronal culture). Bottom: bar graph of motion types (Figure 6—figure supplement 1) for non-exocytosis GluA1-HT-SEP vesicles (gray bars) and pre-exocytosis GluA1-HT-SEP vesicles (black dots) after sLTP. Only trajectories in regions where actin polymerization occurred were used (see Figure 3—figure supplement 3A). No MNI vs sLTP, **p=0.0018; sLTP vs sLTP +LatA, ***p=0.0003; sLTP vs sLTP +MI, **p=0.0017. Significance was determined by Mann-Whitney test. n=9–12 sLTP stimulation experiments (each experiment targets one spine on one neuron) where GluA1-HT-SEP motion and exocytosis in the dendrite are captured immediately after sLTP stimulation. (**C**) Diagram of directional bias analysis. The directional bias of a trajectory can be determined by calculating the angle, theta (Θ), created between the line from the center of mass of a trajectory (CM) to the nearest point on the dendrite path and the line from the CM to the furthest point from the CM. Theta of 90^o^ indicates longitudinal movement while theta of 0^o^ indicates lateral movement. Images: representative trajectories of GluA1-HT vesicles traveling longitudinally under control conditions (Control; top) or laterally after cLTP (cLTP; bottom). (**D**) Directional bias for GluA1-HT-SEP vesicles prior to exocytosis (pre-exocytosis) after sLTP. Top: distribution of theta for vesicles after sLTP. sLTP (blue) vs No MNI (gray), **p=0.0067. Middle: distribution of theta for vesicles after sLTP in the presence of an actin polymerization inhibitor (LatA). sLTP +LatA (blue) vs sLTP (gray), **p=0.0032. Bottom: distribution of theta for vesicles after sLTP in the presence of acute myosin inhibition (MI). sLTP +MI (blue) vs sLTP (gray), **p=0.0078. Lines represent the probability density function of each histogram estimated by kernel density estimation (KDE). Significance was determined by Kolmogorov-Smirnov test. n=31–50 GluA1-HT-SEP trajectories pooled from 9 to 12 sLTP stimulation experiments for each condition. Figure 6—source data 1.Related to Figure 6.

To determine if the concentration of GluA1-HT-SEP vesicles near the site of synaptic activity contributes to increased trafficking of GluA1-HT-SEP to the cell surface, we sparsely labeled GluA1-HT-SEP vesicles with JF dye ligands and simultaneously tracked vesicle motion and exocytosis (Figure 6A and Figure 6—video 1). We sought to determine whether GluA1-HT-SEP vesicles destined for exocytosis (i.e. pre-exocytosis vesicles) are drawn from a local source or from distal loci via long-range active transport immediately prior to exocytosis. If pre-exocytosis GluA1-HT-SEP vesicles are drawn from local sources, they should travel relatively short net distances to the sites of exocytosis. To test this hypothesis, we measured the radius of confinement (the area in which a trajectory is confined) for the trajectories of pre-exocytosis GluA1-HT-SEP vesicles in unstimulated cells (Figure 6B, top bar graph). The trajectories of GluA1-HT-SEP vesicles that undergo exocytosis (Pre-exocytosis) have smaller radii of confinement when compared to vesicles that do not exocytose (Non-exocytosis), demonstrating that GluA1-HT-SEP vesicles are not imported from distal loci immediately prior to exocytosis.

**Figure 6—figure supplement 1.**
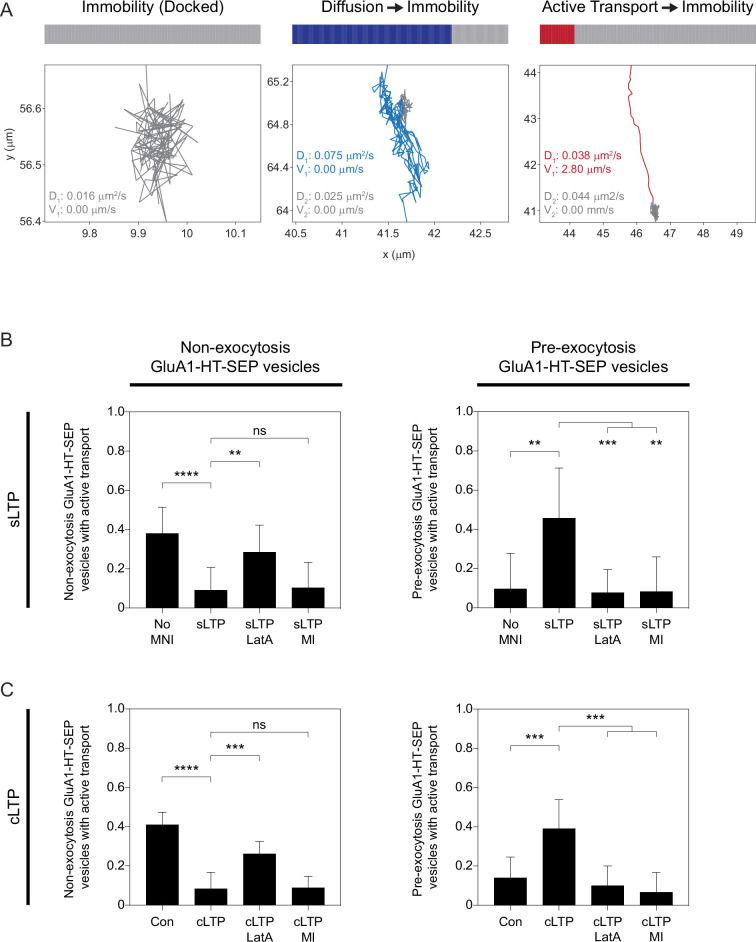
Stimulation increases the fractions of pre-exocytosis GluA1-HT-SEP vesicles exhibiting active transport prior to docking. (**A**) Temporal state sequences (top) and trajectories (bottom) for pre-exocytosis GluA1-HT-SEP vesicles exhibiting immobility, diffusion followed by immobility, and active transport followed by immobility. Pre-exocytosis GluA1-HT-SEP vesicles exhibit at least two motion states before exocytosis, where the final motion state is immobility. We therefore interpret immobility to be vesicle docking prior to exocytosis. (**B**) Fractions of non-exocytosis (left) or pre-exocytosis (right) GluA1-HT-SEP vesicles exhibiting active transport after sLTP, sLTP in the presence of LatA (sLTP +LatA), or sLTP in the presence of pharmacological myosin inhibitors (sLTP +MI). Error bars represent standard deviation. Non-exocytosis: No MNI vs sLTP, ****p<0.0001; sLTP vs sLTP +LatA, **p=0.0017. Pre-exocytosis: No MNI vs sLTP, **p=0.0018; sLTP vs sLTP +LatA, ***p=0.0003; sLTP vs sLTP +MI, **p=0.0017. Significance was determined by Mann-Whitney test. n=9–12 sLTP stimulation experiments (each experiment targets one spine on one neuron) where GluA1-HT-SEP motion and exocytosis in the dendrite are captured immediately after sLTP stimulation. (**C**) Fractions of non-exocytosis (left) or pre-exocytosis (right) GluA1-HT-SEP vesicles exhibiting active transport during cLTP, cLTP in the presence of LatA (cLTP +LatA), or cLTP in the presence of pharmacological myosin inhibitors (cLTP +MI). Error bars represent standard deviation. Non-exocytosis: Control vs cLTP, ****p<0.0001; cLTP vs cLTP +LatA, ***p<0.0001. Pre-exocytosis: Control vs cLTP, ***p=0.0001; cLTP vs cLTP +LatA, ***p=0.0001; cLTP vs cLTP +MI, ***p=0.0001. Significance was determined by Mann-Whitney test. n=9–12 timelapses imaging experiments (each timelapse captures GluA1-HT-SEP motion and exocytosis in one region of dendrite in one neuronal culture) for each condition. Figure 6—figure supplement 1—source data 1.Related to Figure 6—figure supplement 1.

**Figure 6—figure supplement 2.**
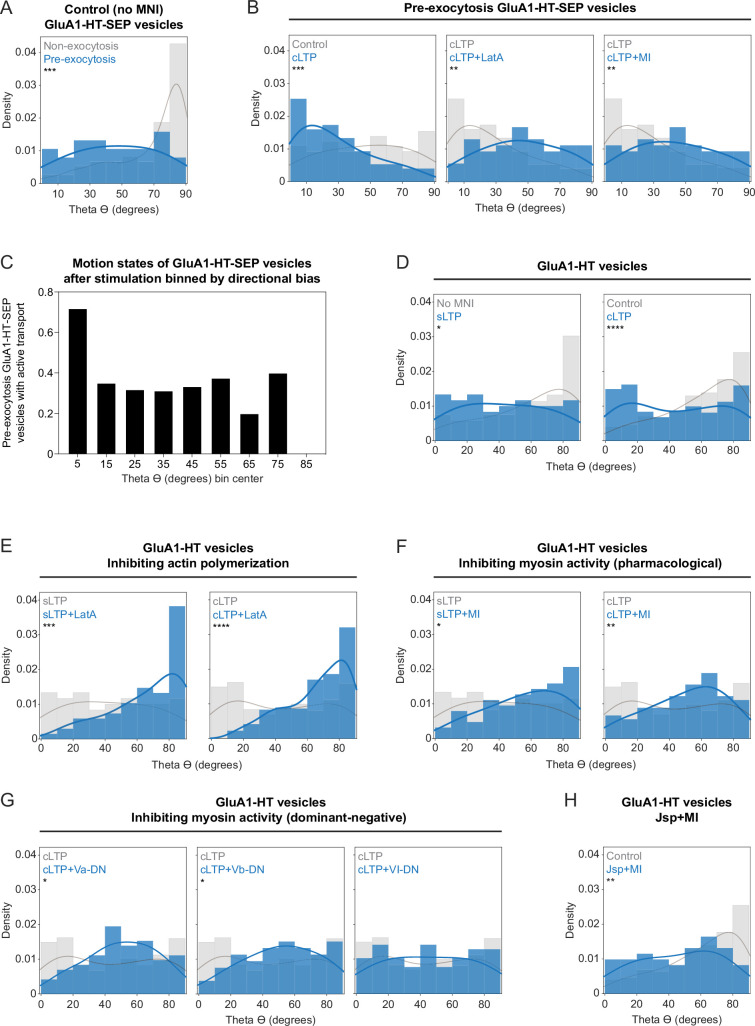
Stimulation changes the directional bias of GluA1-HT vesicles and pre-exocytosis GluA1-HT-SEP vesicles. (**A**) Distributions of theta for non-exocytosis (gray) and pre-exocytosis (blue) GluA1-HT-SEP vesicles after sLTP stimulation in the absence of MNI (No MNI). Lines represent the probability density function of each histogram estimated by kernel density estimation (KDE). ***p=0.0002. n=113 GluA1-HT-SEP vesicle trajectories pooled from 12 sLTP stimulation experiments (each experiment targets one spine on one neuron) where GluA1-HT-SEP motion and exocytosis in the dendrite are captured immediately after sLTP stimulation. (**B**) Distributions of theta for pre-exocytosis GluA1-HT-SEP vesicles during cLTP, cLTP in the presence of LatA (cLTP +LatA), or cLTP in the presence of pharmacological myosin inhibitors (cLTP +MI). Con vs cLTP, ***p=0.0004; cLTP vs cLTP +LatA, **p=0.0011; cLTP vs cLTP +MI, **p=0.0088. n=54–75 GluA1-HT-SEP vesicle trajectories pooled from 9 to 12 timelapses imaging experiments (each timelapse captures GluA1-HT-SEP motion and exocytosis in one region of dendrite in one neuronal culture) for each condition. (**C**) Fraction of pre-exocytosis GluA1-HT-SEP vesicles with active transport after sLTP or cLTP stimulation sorted into bins based on their directional biases. n=125 GluA1-HT-SEP vesicle trajectories pooled from 12 sLTP stimulation experiments and 12 cLTP experiments. (**D**) Distributions of theta for GluA1-HT vesicles after sLTP or cLTP. No MNI vs sLTP, *p=0.0158; control vs cLTP, ****p<0.0001. n=51–59 GluA1-HT vesicle trajectories pooled from six to nine sLTP stimulation experiments and n=227–360 GluA1-HT vesicle trajectories pooled from 12 to 14 cLTP experiments for each condition. (**E**) Distributions of theta for GluA1-HT vesicles after sLTP or cLTP in the presence of LatA. sLTP vs sLTP +LatA, ***p=0.0004; cLTP vs cLTP +LatA, ****p<0.0001. n=59–67 GluA1-HT vesicle trajectories pooled from six to nine sLTP stimulation experiments and n=227–307 GluA1-HT vesicle trajectories pooled from 12 to 14 cLTP experiments for each condition. (**F**) Distributions of theta for GluA1-HT vesicles after sLTP or cLTP in the presence of pharmacological myosin inhibitors (MI). sLTP vs sLTP +MI, *p=0.0263; cLTP vs cLTP +MI, **p=0.0090. n=54–69 GluA1-HT vesicle trajectories pooled from six to nine sLTP stimulation experiments and n=82–360 GluA1-HT vesicle trajectories pooled from 9 to 14 cLTP experiments for each condition. (**G**) Distributions of theta for GluA1-HT vesicles after cLTP in the presence of dominant-negative inhibition of myosin Va (cLTP +Va DN), Vb (cLTP +Vb DN), or VI (cLTP +VI DN). cLTP vs cLTP +Va DN, *p=0.0121; cLTP vs cLTP +Vb DN, *p=0.0205. n=53–360 GluA1-HT vesicle trajectories pooled from 3 to 14 cLTP experiments for each condition. We note that inhibition of myosin VI by expressing its dominant-negative c-terminal domain does not alter the directional bias of vesicles in response to cLTP. Consequently, we believe that the change in directional bias exhibited by vesicles in response to synaptic activity is primarily driven by myosin Va and Vb. (**H**) Distributions of theta for GluA1-HT vesicles in response to Jasplakinolide treatment in the presence of pharmacological inhibitors for myosin (Jsp +MI). **p=0.0095. n=60–227 GluA1-HT vesicle trajectories pooled from three to nine chemical stimulation experiments for each condition. Significance was determined by Kolmogorov-Smirnov test for all distributions. Figure 6—figure supplement 2—source data 1.Related to Figure 6—figure supplement 2.

**Figure 6—video 1.** Timelapse sequence of GluA1-HT-SEP labeled with JF_549_-HTL (after blocking with JF_646_-HTL) in a dendrite after sLTP stimulation. Scale bar, 5 μm. Left: particle motion of GluA1-HT-SEP labeled with JF_549_-HTL (chase; JF_549_). Center: GluA1-HT-SEP exocytosis (SEP). Right: timelapse of GluA1-HT-SEP particle motion (JF_549_; green) merged with GluA1-HT-SEP exocytosis (SEP; magenta). White circles denote sites of exocytosis.

Based on the observations that pre-exocytosis GluA1-HT-SEP vesicles have small search spaces and that stimulation reduces active transport for GluA1-HT vesicles (Figure 1D–E and Figure 2B–C), we anticipated that stimulation would also reduce active transport for pre-exocytosis GluA1-HT-SEP vesicles, and that these vesicles diffuse over short distances to the sites of exocytosis. To test this idea, we used HMM-Bayes to infer the motion of pre-exocytosis GluA1-HT-SEP vesicles after inducing structural plasticity with sLTP stimulation. Pre-exocytosis GluA1-HT-SEP vesicles exhibit two or more motion states, where the final state before exocytosis is immobility (i.e. vesicles are immobilized by docking immediately prior to exocytosis; Figure 6—figure supplement 1A). Thus, we characterized the motion states of vesicles prior to immobility (docking). Similar to GluA1-HT vesicles, non-exocytosis GluA1-HT-SEP vesicles (i.e. vesicles that do not exocytose) exhibit reduced active transport in response to sLTP (Figure 6B, bottom bar graph, No MNI vs sLTP, and Figure 6—figure supplement 1B, Non-exocytosis). Surprisingly, sLTP increased the fraction of pre-exocytosis GluA1-HT-SEP vesicles exhibiting active transport (Figure 6B, bottom line graph, No MNI vs sLTP, and Figure 6—figure supplement 1B, Pre-exocytosis). Similarly, cLTP also increased active transport of pre-exocytosis GluA1-HT-SEP vesicles (Figure 6—figure supplement 1C). These results show that synaptic activity stimulates the active transport of GluA1-HT-SEP vesicles to exocytic sites, even though the net distances they travel are short.

As active transport delivers vesicular cargo much faster than diffusion, this finding is consistent with the model that AMPARs need to be rapidly trafficked to exocytic sites during synaptic activity in order to maintain a rapidly accessible membrane-bound reservoir of receptors (Huganir and Nicoll, 2013). Nevertheless, it is striking that synaptic activity increases the active transport of pre-exocytosis GluA1-HT-SEP vesicles while simultaneously confining the motion of non-exocytosis GluA1-HT-SEP vesicles. AMPAR-containing endosomes are primarily trafficked along to the length of dendrites by microtubule-based motors (EstevesdaSilva et al., 2015; Setou et al., 2002; Hoerndli et al., 2013), but can also be recruited by myosin Va and Vb to the sites of exocytosis during LTP induction (Correia et al., 2008; Wang et al., 2008). Thus, we hypothesized that actin polymerization in the dendritic shaft plays a dual role during synaptic activity by: (1) disrupting microtubule-based transport of GluA1 vesicles near synaptic activity (increasing the concentration of GluA1 vesicles); and (2) acting as a substrate for the myosin-based transport of a minority of vesicles to exocytic sites. This may explain the different motion states we observe between pre- and non-exocytosis GluA1-HT-SEP vesicles during stimulation. Interestingly, we primarily observe GluA1-HT vesicles undergoing transport parallel to the length of the dendritic shaft (longitudinal motion) prior to stimulation (Figure 1D, control and Figure 2B, Pre-sLTP). After stimulation, we find GluA1-HT vesicles that exhibit motion perpendicular to the length of the dendritic shaft (lateral motion; Figure 1D, cLTP and Figure 2B, Post-sLTP). Consequently, we asked if vesicles that changed their directional bias in response to stimulation were in fact pre-exocytosis vesicles being transported to their exocytic sites by myosin.

To examine this possibility, we first measured the directional bias, described by the angle theta, of pre-exocytosis GluA1-HT-SEP vesicle trajectories in response to sLTP-stimulated synaptic activity (Figure 6C). Theta of ~90^o^ indicates that the vesicle is moving parallel to the dendritic shaft (longitudinal motion; Figure 6C, top trajectory), whereas theta of ~0^o^ indicates the vesicle is moving perpendicular to the dendritic shaft (lateral motion; Figure 6C, bottom trajectory). When we examine the directional bias for non-exocytosis GluA1-HT-SEP vesicles in the absence of sLTP stimulation, we find a strong bias for longitudinal motion (Figure 6—figure supplement 2A, Non-exocytosis vesicles). By contrast, pre-exocytosis GluA1-HT-SEP vesicles do not exhibit strong biases for either longitudinal or lateral motion in the absence of stimulation (Figure 6D, top histogram, No MNI, and Figure 6—figure supplement 2A, Pre-exocytosis vesicles), indicating that vesicles travel in random directions to exocytic sites in the absence of synaptic activity. However, when synaptic activity is induced by sLTP, pre-exocytosis GluA1-HT-SEP vesicles have a strong bias for lateral motion (Figure 6D, top histogram, sLTP). Likewise, cLTP induction also leads to increased lateral motion for pre-exocytosis GluA1-HT-SEP vesicles (Figure 6—figure supplement 2B, Control vs cLTP). These results show vesicles move laterally to exocytic sites in response to synaptic activity, which we speculate is due to vesicles moving from the center to the periphery of the dendritic shaft.

We then sought to determine whether increased lateral motion during synaptic activity is driven by myosin. We first examined the motion states of pre-exocytosis GluA1-HT-SEP vesicles that move laterally during stimulation and find most exhibit active transport (Figure 6—figure supplement 2C). This result suggests that GluA1-HT-SEP vesicles move by motor-based transport to exocytic sites. Moreover, when actin polymerization or myosin activity are inhibited, the fraction of pre-exocytosis GluA1-HT-SEP vesicles exhibiting active transport is significantly reduced (Figure 6B, bottom line graph, sLTP +LatA and sLTP +MI). Next, we tested the effect of inhibiting actin polymerization on directional bias and find that LatA strongly reduces the lateral motion of pre-exocytosis GluA1-HT-SEP vesicles in response to sLTP (Figure 6D, middle histogram, sLTP +LatA). Similarly, pharmacological inhibition of myosin also blocked lateral motion in response to sLTP (Figure 6D, bottom histogram, sLTP +MI). Inhibition of either actin polymerization or myosin activity also prevents increased lateral motion of pre-exocytosis vesicles in response to cLTP (Figure 6—figure supplement 2B, cLTP vs cLTP +LatA and cLTP +MI). Together, these findings demonstrate that pre-exocytosis GluA1-HT-SEP vesicles are transported laterally by myosin to the sites of exocytosis in response to synaptic activity.

Lastly, we sought to confirm our finding that actin polymerization itself is sufficient to block longitudinal motion. We examined theta for GluA1-HT vesicles in response to stimulation and find a decrease in longitudinal motion and increase in lateral motion (Figure 6—figure supplement 2D, sLTP and cLTP), similar to what we observe for pre-exocytosis vesicles. However, when GluA1-HT vesicles are treated with LatA during stimulation, we observe a decrease in lateral motion and a strong increase in longitudinal motion (Figure 6—figure supplement 2E, sLTP +LatA and cLTP +LatA). By contrast, inhibition of myosin during stimulation reduces lateral motion but does not increase longitudinal motion (Figure 6—figure supplement 2F, sLTP +MI and cLTP +MI, and Figure 6—figure supplement 2G). Moreover, inducing actin polymerization with Jasplakinolide (Jsp) while blocking myosin activity reduces longitudinal motion without increasing lateral motion (Figure 6—figure supplement 2H, Jsp +MI). These observations support our conclusion that F-actin is necessary and sufficient to confine GluA1 vesicle motion near sites of synaptic activity. Nevertheless, myosin also promotes the surface expression of GluA1 by mediating its active transport to the sites of exocytosis.

## Discussion

In this study, we have developed a novel method to identify, track and characterize the motion of vesicles containing GluA1, enabling us to better understand how AMPARs are delivered specifically to sites undergoing plasticity. We use homology-independent targeted integration (HITI) to tag endogenous GluA1 with HaloTag (GluA1-HT) and then a block-and-chase labeling protocol with Janelia Fluor (JF) dye ligands to achieve a sparse labeling density suitable for the detection of GluA1-HT vesicles. We then utilize single-particle tracking (SPT) followed by hidden Markov modeling with Bayesian model selection (HMM-Bayes) to describe the motion of GluA1-HT vesicles during chemical and structural LTP (cLTP and sLTP). Using this strategy, we find that GluA1-HT vesicles become confined by actin polymerization in the dendritic shaft proximal to sites of stimulation, resulting in an increased intracellular reservoir of GluA1-HT near these sites. Using a pHluorin-HaloTag fusion with GluA1 (GluA1-HT-SEP), we examine how local vesicular reservoirs of GluA1 contribute to GluA1 exocytosis. We find that pre-exocytosis GluA1-HT-SEP vesicles undergo short-range transport perpendicular to the length of the dendritic shaft near sites of stimulation in a manner dependent on both actin polymerization and myosin activity.

**Figure 7.**
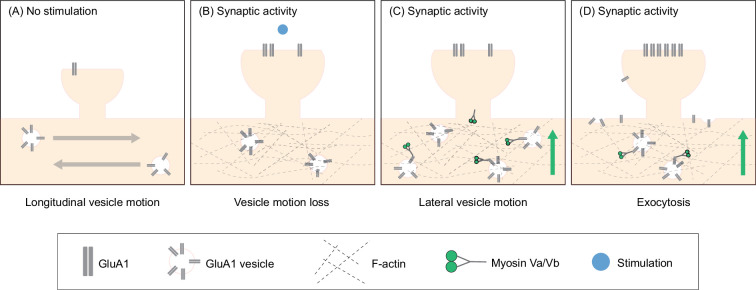
Actin polymerization in the dendritic shaft proximal to the site of synaptic activity promotes GluA1 exocytosis by increasing the local pool of GluA1 vesicles and facilitating myosin-dependent transport to the dendrite periphery. (**A**) Under unstimulated conditions, GluA1 vesicles are transported longitudinally along the dendritic shaft. (**B**) When synaptic activity is induced, actin polymerizes in the dendritic shaft near the site of activity. Actin polymerization disrupts the longitudinal motion of vesicles resulting in an increased pool of GluA1 vesicles near the site of activity. (**C**) Myosin V, which is inactive and sequestered in dendritic spines in unstimulated conditions, is activated by calcium influx during synaptic activity. Myosin subsequently translocates to the base of the dendritic spines. (**D**) Myosin V recruits GluA1 vesicles to the periphery resulting in increased exocytosis of GluA1.

Based on these findings, we propose a new model in which neurons utilize actin polymerization in the dendritic shaft to specify the location to which AMPARs are delivered during synaptic activity. First, actin polymerization occurs in the dendritic shaft proximal to the site of synaptic activity, resulting in molecular crowding in the dendritic cytoplasm at this location (Figure 7A–B). The increased crowding inhibits the longitudinal motion of GluA1 vesicles, concentrating intracellular GluA1 near the site of synaptic activity (Figure 7A–B). F-actin then acts as a substrate for myosin Va and Vb – activated by the influx of calcium (Correia et al., 2008; Wang et al., 2008) – to recruit GluA1 vesicles to the dendrite membrane (Figure 7C). Here, GluA1 vesicles undergo exocytosis, increasing the amount of surface bound GluA1 that can then diffuse into synapses (Figure 7D).

Our labeling strategy has key advantages over previously described methods that enable us to image and track GluA1 vesicles. Primarily, we edit genomic copies of *Gria1* to express GluA1-HT and thus avoid pitfalls associated with expressing tagged GluA1 from a plasmid – overexpressing GluA1 can lead to mislocalization and excess formation of calcium permeable GluA1 homomers (Diering and Huganir, 2018), altering conductance and neuronal activity. The low level of GluA1-HT expression driven by native *Gria1* promoters is also advantageous because it enables us to achieve sparse particle labeling through a block-and-chase protocol without photobleaching. As photobleaching removes all signal from a designated area, particles are tracked as they travel from outside to inside this region. Consequently, data from photobleaching experiments may be biased towards fast moving particles (i.e. those with active transport) and may omit slowly diffusing particles. HITI can potentially introduce indels into target genes and knock out copies of *Gria1* in some neurons (Suzuki et al., 2016), but we find a low frequency of indels around the insertion site of HaloTag (Figure 1—figure supplement 1D), and also that *Gria1* expression levels in transfected neurons are similar to those in untransfected neurons (Figure 1—figure supplement 2B). These observations indicate that HITI-mediated editing of *Gria1* does not often lead to unwanted mutations, especially those that knock out copies of *Gria1*. Nevertheless, tagging strategies with higher accuracy (e.g. vSlender; Nishiyama et al., 2017) could be viable alternatives to HITI.

Our observations of GluA1 vesicle trafficking differ somewhat from previous reports (EstevesdaSilva et al., 2015; Bowen et al., 2017; Hangen et al., 2018). Hangen et al., 2018 found that GluA1 vesicles undergoing active transport had decreased velocity and paused (i.e. temporarily lost active transport) more frequently during cLTP and photostimulation. By contrast, we find vesicles switch their motion state to diffusion and rarely switch back to active transport (i.e. vesicles stably lose active transport) in response to cLTP and sLTP. Vesicles also exhibit reduced diffusion coefficients after stimulation. Based on these differences, we conclude that GluA1 vesicles are confined, not paused, near sites of synaptic activity. These discrepancies are likely attributable to differences in our methodological approaches. Importantly, our labeling and analysis strategy enabled us to characterize the diffusion coefficient and state-switching probabilities of GluA1-HT vesicles, not just parameters associated with active transport. In addition, we use a specific MNI uncaging protocol to stimulate spine plasticity (not just calcium transients). We find that this protocol also induces actin polymerization in the dendritic shaft, which we demonstrate is the mechanism underlying vesicle confinement.

The precise mechanistic details of how actin polymerization is stimulated in the dendritic shaft and how it regulates GluA1 vesicles during synaptic activity remain to be determined. F-actin is found at the base of dendritic spines (Schätzle et al., 2018), and in the dendritic shaft in shaft synapses (van Bommel et al., 2019) and in periodic submembrane actin rings (Lavoie-Cardinal et al., 2020). Whether these F-actin networks remodel to regulate GluA1 vesicle motion during synaptic activity is not known. Furthermore, it is unclear if F-actin also coordinates the localization of other proteins and organelles near synaptic activity. For example, F-actin patches position lysosomes in shaft synapses to support AMPAR turnover (Goo et al., 2017; van Bommel et al., 2019), and thus could play a similar role near sites of synaptic activity. However, if actin-induced molecular crowding is generally obstructive to motion, additional mechanisms may be necessary to ensure only relevant particles localize to sites of synaptic activity. For example, myosin cargo adaptors that interact with GluA1 may further enhance the specificity of GluA1 transport (Correia et al., 2008; Hammer and Sellers, 2012), while other motors might enable GluA1-negative vesicles to bypass F-actin blockades (Ferro et al., 2019). Importantly, AMPARs are transported to multiple loci along a single dendrite, suggesting there must also be a mechanism to ensure not all GluA1 vesicles are trapped at a single synapse. One possibility is that there is a sufficient pool of GluA1 vesicles undergoing anterograde and retrograde transport to reach multiple synapses. Further investigation could clarify how actin polymerization is regulated in the dendritic shaft, and how F-actin filters specific cargo in response to synaptic activity.

Previous studies have found that AMPARs are primarily transported along the length of the dendrites on microtubules (EstevesdaSilva et al., 2015; Setou et al., 2002; Hoerndli et al., 2013; Hoerndli et al., 2015), but we and others find that short-ranged transport near the site of synaptic activity is mediated by Myosin Va/b (Correia et al., 2008; Wang et al., 2008). These observations suggest that GluA1 vesicles are transferred from microtubules to actin for local transport. The exact mechanism for this exchange is not known but our findings indicate that F-actin-induced crowding results in the detachment of GluA1 vesicles from microtubules prior to attachment and transport on F-actin. Thus, F-actin enhances local transport by acting as a substrate for myosin-based cargos, but also by creating a cytoplasmic environment that strongly disfavors microtubule-based transport (preventing transport away from the stimulated site). Such a mechanism could have important implications for how cargo in general is transferred since changes to the subcellular environment could alter modes of transport indirectly rather than through the interactions between motors, cargo, and cytoskeleton.

The precise control of synaptic protein trafficking is vital to synaptic transmission, and thus learning and memory. Nevertheless, many mechanisms regulating synaptic protein trafficking remain to be fully understood. Here, we identify a novel mechanism through which actin polymerization in the dendritic shaft can regulate the surface expression of GluA1 specifically at sites with stimulating inputs. Because F-actin can exert direct and indirect effects on a variety of particles in the dendritic cytoplasm, our findings raise interesting questions regarding whether actin polymerization is a general mechanism to coordinate the delivery of proteins during synaptic plasticity. Elucidating whether and how actin regulates the motion of proteins in the dendritic shaft during neuronal activity could help us better understand the cellular bases for learning and memory.

## Materials and methods

Animal work was conducted according to the Institutional Animal Care and Use Committee guidelines of Janelia Research Campus (IACUC protocol #21–0206).

### Plasmid construction

To generate the HaloTag donor construct (px552-sg-Gria1-HT), miRFP670 was first amplified from pBAD/His-miRFP670 (Shcherbakova et al., 2016) and cloned into the KpnI and EcoRI sites of PX552 (Swiech et al., 2015). HaloTag was then amplified from LZ10 PBREBAC-H2BHalo (Li et al., 2016) using primers that add glycine-serine linkers and *Gria1* sequences to be targeted by Cas9. This HaloTag amplicon was cloned into the XbaI site of PX552 to generate px552-Gria1-HT. To introduce the single guide RNA (sgRNA) insert into px552-Gria1-HT for Cas9 to target *Gria1*, we ordered the 20 bp *Gria1* sequence to be targeted by Cas9 with 5’ overhangs (ACC and AAC) from Integrated DNA Technologies (IDT, Newark, NJ), and cloned the target sequence into the SapI sites of px552-Gria1-HT (as previously described in Swiech et al., 2015), creating px552-sg-Gria1-HT. For the HaloTag-SEP donor construct (px552-sg-Gria1-HT-SEP), HaloTag was amplified from LZ10 PBREBAC-H2BHalo and SEP was amplified from pAS1NB c Rosella I (Rosado et al., 2008). We used primers that add glycine-serine linkers and *Gria1* sequences to be targeted by Cas9 to the 5’-end of HaloTag and the 3’-end of SEP, and primers that introduce a short glycine-serine linker between HaloTag and SEP. The PCR amplicons for HaloTag and SEP were fused using overlap extension PCR, and cloned into the MfeI and NheI sites of px552-sg-Gria1-HT to generate px552-sg-Gria1-HT-SEP. The Cas9 expression construct (PX551) was previously described (Swiech et al., 2015). To generate the tractin construct (hSyn-tractin), F-tractin-mNeongreen (also known as ITPKA-mNeongreen) was amplified from the ITPKA-mNeongreen construct (Chertkova et al., 2017) and cloned into the KpnI and BsrGI sites of px552-Gria1-HT. Additional sequences between the inverted terminal repeats (ITRs) that are not related to the expression of F-tractin-mNeongreen (i.e. the sgRNA insert and the HaloTag donor sequence) were removed by digesting the construct with MluI and NheI and inserting a short double-stranded DNA oligo with 5’ overhangs that are complementary to MluI and NheI, creating hSyn-tractin. The GEM-HT-ST construct (hSyn-GEM) was created by amplifying PfV from pCMV-PfV-Sapphire-IRES-DSRed (Delarue et al., 2018), fusing PfV, HaloTag, and SNAP-tag by overlap extension PCR, and cloning the product into the KpnI and BsrGI sites of hSyn-tractin, creating hSyn-GEM. To generate a construct to express the dominant-negative c-terminal domain of myosin VI, we purchased a gBlock of human myosin VI corresponding to amino acids 835–1285 (IDT, Newark, NJ; Correia et al., 2008) and cloned the gBlock into the BsrGI and EcoRI sites of the pEGFP-C1 vector (pEGFP-MyosinVI-Ctail; Clontech). To generate pCAG-GluA1-P2A-GFP, GluA1 was amplified from pCMV2-SEP-GluA1 (Blanco-Suarez and Hanley, 2014) and fused to P2A-GFP by overlap extension PCR. This amplicon was cloned into the SphI and XhoI sites of pCAGGS (Niwa et al., 1991). To generate pCAG-GluA1-HT-P2A-GFP, HaloTag was amplified from px552-Gria1-HT and fused to the N-terminal of GluA1 by overlap extension PCR. This amplicon was cloned into the SphI and BstBI sites of pCAG-GluA1-P2A-GFP. To generate pCAG-GluA1-HT-SEP-P2A-mScarlet, HaloTag-SEP was first amplified from px552-sg-Gria1-HT-SEP and fused to the N-terminal of GluA1 by overlap extension PCR. This amplicon was then cloned into the SphI and BstBI sites of pCAG-GluA1-P2A-GFP to generate pCAG-GluA1-HT-SEP-P2A-GFP. Lastly, we replaced GFP with mScarlet by cloning P2A-mScarlet into the AgeI and XhoI sites of pCAG-GluA1-HT-SEP-P2A-GFP. All plasmids were propagated in Stbl3 *E. coli* cells.

### Cell culture and transfection

Human embryonic kidney (HEK) 293T cells were cultured in Dulbecco’s Modified Eagle Medium (DMEM) with 10% fetal bovine serum (FBS) at 37 °C and 5% CO_2_. At 70% confluence, HEK293T cells were dissociated and transiently transfected in suspension with plasmids expressing rat GluA1 (pCAG-GluA1-P2A-GFP), GluA1-HT (pCAG-GluA1-HT-P2A-GFP), or GluA1-HT-SEP (pCAG-GluA1-HT-SEP-P2A-Scarlet) at 0.4 μg using Lipofectamine 3000. Cells were then plated onto poly-ʟ-lysine functionalized coverslips. After 48 hr of recovery, cells were used for voltage-clamp recordings. Dissociated hippocampal neurons were prepared from P0 Sprague-Dawley rat pups (Charles River). Hippocampi were dissected out and digested with papain in dissection solution (10 mM HEPES in Hanks’ balanced salt solution; HBSS). After digestion, the tissues were gently triturated in minimum essential media (MEM) with 10% FBS and filtered with a 40 μm cell strainer. The cell density and viability were determined by labeling neurons with trypan blue and counting cells with a Countess 3 cell counter. To transfect neurons via electroporation, 500,000 neurons were resuspended in 20 μL complete P3 (P3 solution with supplement) and moved to a cuvette containing 0.5 μg of each plasmid to be transfected. Samples were then electroporated using an Amaxa 4D-Nucleofector with settings CU-110. 80 μL of plating media (MEM with 10% fetal bovine serum, 28 mM glucose, 2.4 mM NaHCO_3_, 100 μg/mL transferrin, 25 μg/mL insulin, 2 mM ʟ-glutamine) was added to the cuvette immediately after electroporation and samples were allowed to recover for 5 min at 37 °C and 5% CO_2_. The electroporated sample was then removed from the cuvette and added to an Eppendorf tube with 500 μL plating media. Approximately 50,000–75,000 cells were spread onto the poly-ᴅ-lysine-coated coverslip of a 10 mm MatTek dish. Six hours later, plating media was replaced by 2 mL NbActiv4 neuronal culture media. Half of the neuronal culture media was removed and replaced with fresh NbActiv4 every week until the neurons were used.

### Adeno-associated virus (AAV) packaging and transduction

px552-sg-Gria1-HT, px552-sg-Gria1-HT-SEP, PX551, hSyn-tractin, hSyn-GEM, hSyn-GCaMP6s, CAG-tdTomato, and hSyn-GFP were packaged into adeno-associated virus (AAV) by the Janelia Research Campus Viral Services Shared Resource. Briefly, HEK293T cells were transiently transfected with 84 µg of DNA at a ratio of pHelper plasmid:capsid plasmid:AAV construct = 3:2:5. Transfected cells were replenished with fresh serum- and phenol-free DMEM at 6–8 hr post-transfection and incubated for 3 days at 37 °C and 5% CO_2_. AAVs were collected from both cells and supernatant and purified by two rounds of continuous cesium chloride density gradient. AAV preparations were dialyzed, concentrated to 100 µL, and sterilized by filtration. The final viral titers were measured by quantitative PCR (qPCR) on the inverted terminal repeats (ITRs). AAVs were pseudotyped with AAV2/9, SL1 (retro), or rh10 capsids. For AAV2/9-hSyn-tractin, AAV2/9-hSyn-GEM, AAV2/9-hSyn-GCaMP6s, AAV2/9-CAG-tdTomato, or AAV2/9-hSyn-GFP, 1x10^8^ genomic copies of virus was mixed into 50 μL of NbActiv4 and then added directly into neuronal cultures between 3 and 7 days in vitro (DIV3-7). Fluorescence signals from these reporters were detectable 3 days after transduction.

### Homology-independent targeted integration (HITI)

We used homology-independent targeted integration (HITI) to insert HaloTag or HaloTag-SEP into the endogenous loci of *Gria1* (see Figure 1A and Figure 1—figure supplement 1). px552-sg-Gria1-HT, px552-sg-Gria1-HT-SEP, and PX551 were delivered into cultured neurons by either electroporation or AAV-mediated transduction. For electroporation, 0.5 μg of px552-sg-Gria1-HT/HT-SEP and 0.5 μg PX551 were mixed with neurons suspended in complete P3 and electroporated as described above (see Cell culture and transfection). For AAV-mediated transduction, 1x10^10^ genomic copies of rh10-px552-sg-Gria1-HT/HT-SEP virus and 1x10^9^ genomic copies of rh10-PX551 virus were mixed into 50 μL of NbActiv4 and then added directly into neuronal cultures at DIV3. HaloTag and HaloTag-SEP positive cells could be observed 7 days after transduction. To determine the HaloTag knock-in efficiency (HaloTag KI), we first counted the number of neurons expressing HaloTag and the number of neurons expressing the miRFP670 transfection marker (also expressed from px552-sg-Gria1-HT/HT-SEP) in a dish. We then calculated the knock-in efficiency by dividing the number of HaloTag^+^ neurons with the number of neurons that have been transfected with both px552-sg-Gria1-HT and PX551:

$$HaloTag\;KI=\frac{\#\;HaloTag^{+}\;neurons}{{\left(\frac{\#\;miRFP670^{+}\;neurons}{\#\;total\;neurons}\right)}^{2}\times \#\;total\;neurons}$$

Because PX551 has no transfection marker, we assumed that the rate of PX551 transfection is equal to the rate of px552-sg-Gria1-HT transfection. Consequently, we extrapolate that the rate of co-transfection is equal to the rate of px552-sg-Gria1-HT transfection squared.

### Sequencing

To determine the rate of indels in *Gria1* caused by HaloTag insertion, we first extracted genomic DNA from transfected neurons using a DNEasy Blood and Tissue Kit (Qiagen). The insertion sites of HaloTag were amplified using primers that flanked either the 5’ or 3’ end of HaloTag. For the 5’ end, the forward primer sits 123 bp upstream of HaloTag in Exon 6, while the reverse primer sits 442 bp downstream of the insertion site inside HaloTag. For the 3’ end, the forward primer sits 513 bp upstream of the insertion site inside HaloTag, while the reverse primer sits 93 bp downstream of the insertion site in Exon 6. Amplicons were then cloned into pUC vectors and transformed into *E. coli*. Single colonies were selected and the amplicons flanking the insertion sites were sent to GENEWIZ (Azenta Life Sciences) for Sanger sequencing. To identify examples in which HaloTag was inserted in the incorrect orientation, we reversed the HaloTag primers. While we could amplify mock plasmid DNA with HaloTag fused to *Gria1* in the incorrect orientation, we failed to amplify genomic DNA, indicating that the majority of HaloTag insertion are in the correct orientation.

### Janelia Fluor (JF) dye labeling

Janelia Fluor 549 HaloTag ligand (JF_549_-HTL), Janelia Fluor 646 HaloTag ligand (JF_646_-HTL), and cell-membrane impermeable Janelia Fluor 549 HaloTag ligand (JF_549_i-HTL) were kind gifts from Dr. Luke Lavis. Dyes were reconstituted in DMSO and diluted to working concentrations of 10 nM (JF_549_-HTL and JF_549_i-HTL) or 20 nM (JF_646_-HTL) in imaging buffer (150 mM NaCl, 2 mM CaCl_2_, 2 mM MgCl_2_, 5 mM KCl, 10 mM HEPES, 30 mM ᴅ-glucose, pH 7.4) before use. To label HaloTag expressed in rat hippocampal neurons, NbActiv4 was removed and replaced with imaging buffer containing working concentrations of JF dye-HTL. Neurons were incubated with JF dye-HTL for 30 min at 37 °C and 5% CO_2_. Cells were then washed three times with imaging buffer and allowed to incubate for another 30 min at 37 °C and 5% CO_2_. After the second incubation, cells were washed an additional three times with imaging buffer and then used for stimulation or imaging. For block-and-chase labeling experiments, neurons expressing HaloTag were first labeled with 20 nM JF_646_-HTL under 50 nM cycloheximide (CHX) in NbActiv4 for 1 hr at 37 °C and 5% CO_2_. JF_646_-HTL and CHX were removed by rinsing neurons with NbActiv4 three times. Neurons were then incubated in NbActiv4 for 3 hr at 37 °C and 5% CO_2_ to allow protein synthesis to recover. After the recovery period, neurons were labeled with 10 nM JF_549_-HTL in imaging buffer for 30 min at 37 °C and 5% CO_2_. Finally, neurons were rinsed an additional three times with imaging buffer and stimulated or imaged.

### Immunofluorescence

Cultured rat hippocampal neurons were fixed between DIV12 and 21. Neurons were fixed with fixation buffer (1 x PBS, 4% PFA, 4% sucrose, 1 mM MgCl_2_, 0.1 mM CaCl_2_) for 20 min. Fixation solution was quenched with 0.1 M glycine in PBS-MC (1xPBS, 1 mM MgCl_2_, 0.1 mM CaCl_2_) for 10 min, and neurons were washed three times for 5 min each wash with PBS-MC. Neurons were then simultaneously permeabilized and blocked with 0.1% saponin blocking buffer (0.1% saponin and 5% normal goat serum in PBS-MC) for 1 hr at room temperature. Neurons were labeled with primary antibodies in 0.02% saponin blocking buffer overnight at 4 °C. Neurons were washed three times for 10 min each wash with 0.02% saponin blocking buffer. Neurons were labeled with secondary antibodies in 0.02% saponin blocking buffer for 1 hr at room temperature. Neurons were then washed three times for 10 min each wash with 0.02% saponin blocking buffer and an additional three times for 5 min each wash with PBS. After labeling, neurons were mounted in Prolong Diamond Antifade Mountant for 24 hr. Stimulation emission depletion (STED) microscopy was performed on an Abberior Expert Line STED microscope equipped with 405/440/485/561/640 nm laser lines for illumination and 595/775 nm laser lines for depletion. Emitted light was collected with four spectral avalanche photodiodes (APDs). A 60 x Plan Apochromat oil-immersion objective (NA = 1.42) was used for all STED imaging. The pinhole size was set to 0.71 airy units (AU). A pixel size of 20 nm was used. 20% depletion laser was used in *xy* and 10% was used in *z*.

### Colocalization analysis

To examine colocalization between HaloTag and GluA1, we performed intensity-based colocalization on antibody-labeled immunofluorescence images using Imaris. STED images were saved as TIFFs and imported to Imaris. To perform colocalization analysis in a specific neuron of interest (e.g. a neuron expressing HaloTag), we used Imaris to create a surface object of the cell of interest and then masked signal outside of the surface object. We then applied an automatic intensity threshold using Otsu’s method – an established thresholding method that enables us to minimize subjective analysis – to each individual fluorescence channel to be used for colocalization analysis. Using these threshold levels, we created a new volume channel corresponding to the colocalizing voxels between the two fluorescence channels. We report both the Pearson’s coefficient (which indicates whether the intensities of two signals co-vary) and the Manders’ coefficients (which indicate whether two signals overlap). For punctate signals that co-occurred inside a spine, but did not colocalize (i.e. HaloTag/GluA1 and postsynaptic density markers), we measured the distance between fluorescence signals using Abberior Imspector. First, images from the two channels (e.g. TRITC for HaloTag and Cy5 for PSD-95) were overlaid and a line profile was applied to adjacent signals (i.e. a single line was drawn through the center of an orange puncta and far-red puncta) in a single spine. We then applied a gaussian fit to each fluorescence intensity profile and measured the distance between the two peaks.

### Hybridization chain reaction RNA fluorescence in situ hybridization (HCR RNA-FISH)

Probes targeting *Rattus norvegicus Gria1* and HaloTag mRNA were designed by Molecular Instruments. Cultured rat hippocampal neurons were fixed as described (see Immunofluorescence). Neurons were permeabilized with 0.1% Triton X-100 in PBS-MC for 15 min. Neurons were washed three times with PBS-MC for 5 min each wash and then two times with 2 x SSC (0.3 M NaCl and 0.03 M sodium citrate) for 5 min each wash. Neurons were next incubated for 30 min at 37 °C in 30% probe hybridization buffer (30% formamide, 5 x SSC, 9 mM citric acid pH 6, 0.1% Tween-20, 50 μg/mL heparin, 1 x Denhardt’s solution, 10% low molecular weight dextran sulfate). *Gria1* and HaloTag mRNA were then labeled with *Gria1-* and HaloTag-targeting hybridization probes in 30% probe hybridization buffer for 12 hr at 37 °C. After hybridization, neurons were washed four times with 30% probe wash buffer (30% formamide, 5 x SSC, 9 mM citric acid pH 6, 0.1% Tween-20, 50 μg/mL heparin) and three times with 5 x SSCT (5 x SSC, 0.1% Tween 20) for 5 min each wash at room temperature. Neurons were then incubated with amplification buffer (5 x SSC, 0.1% Tween-20, 10% low molecular weight dextran sulfate) for 30 min at room temperature. Hybridization probes were then amplified with amplification probes conjugated with Alexa 488 or Alexa 546 in amplification buffer for 45 min at room temperature. After amplification, neurons were washed two times with 5 x SSCT for 5 min each, two times with 5 x SSCT for 30 min each, and once with 5 x SSCT for 5 min at room temperature. After HCR RNA-FISH labeling, cells were mounted in Prolong Diamond Antifade Mountant for 24 hr. Confocal images of the labeled samples were taken with an inverted Carl Zeiss LSM 880 microscope equipped with 405/488/561/594/633 nm laser lines for illumination, and 2 multi-Alkali photomultiplier tubes (PMTs) and a 32-channel spectral gallium arsenide phosphide (GaAsP) PMT. QUASAR detection windows were adjusted optimally for each fluorophore. A 63 x Plan Apochromat oil-immersion objective (NA = 1.4) was used for all HCR RNA-FISH imaging. The pinhole size was set to 1 airy unit (AU) based on the longest emission wavelength detected.

### mRNA quantification and correlated localization analysis

Fluorescent puncta representative of individual mRNA were identified and counted using the FISH-Quant program for MATLAB (Mueller et al., 2013). First, neurons were identified using JF_646_-HTL labeled GluA1-HT and manually outlined. Overlapping neurons were removed from analysis. A Gaussian kernel filter was applied to HCR RNA-FISH images to enhance spot-like features. A pre-detection threshold was determined based on the dimmest and brightest pixel on each image and applied to the image. Additional false positives were then removed based on their low quality score. Pre-detected spots were then fit with a 3D Gaussian function. Detected spots were then visually inspected for one neuronal cell body to determine if the detection parameters were adequate, after which the detection parameters were applied to the remaining cells in the image to quantify the number of spots in each cell. We used object-based colocalization to determine whether spots from two channels (i.e. HaloTag mRNA labeling and *Gria1* mRNA labeling) have correlated localization (Figure 1—figure supplement 2C). Two-channel images were first deconvolved with Zen Black and then imported to Imaris. mRNA in each channel were detected using the Imaris Spot Detection algorithm with a default quality filter for thresholding. Detected spots were visually inspected. We then examined the colocalization of thresholded spots from the two channels. A distance cutoff (i.e. the maximum distance within which two spots can be considered correlated in their localization) of 0.5 μm was used, as this corresponds to a physical distance of 1470 linearized basepairs (half the maximum distance in which probes from the two channels can be separated if they are both on the same mRNA – i.e. if *Gria1-*HaloTag is synthesized as a single mRNA). We report the percentage of spots in one channel that are within 0.5 μm of spots from the second channel and vice versa as a metric to determine whether the two labels have hybridized to a single mRNA (i.e. *Gria1-*HaloTag).

### Single-particle tracking

GluA1-HT and GluA1-HT-SEP vesicles were imaged with a Nikon Eclipse TiE inverted microscope equipped with 405/488/561/642 nm laser lines, three iXon Ultra 897 electron multiplying charge-coupled device (EMCCD) cameras connected via a tri-cam splitter for simultaneous multicolor acquisition, an automatic total internal reflection fluorescence (TIRF) illuminator, and a perfect focusing system. A Tokai Hit Stage Top Incubator was used to maintain constant environmental conditions of 5% CO_2_ and 37 °C during imaging. A 100 x TIRF Apochromat oil-immersion objective (NA = 1.49) was used for imaging. To image GluA1-HT and GluA1-HT-SEP vesicles, we employed highly inclined and laminated sheet (HILO) illumination. Specifically, the TIRF illuminator was adjusted to deliver the laser beam with an incident angle smaller than the total internal reflection angle, which generated a highly inclined light sheet centered on the focal plane. Compared with standard epifluorescence illumination, HILO illumination reduces background from out-of-focus excitation. Images were acquired at 20 Hz. The *xy* pixel size for all GluA1-HT and GluA1-HT-SEP single-particle tracking experiments was 160 nm. 2D single-particle tracking was performed with a custom MATLAB program. Single molecule localization (x,y) was obtained through 2D Gaussian fitting and tracking was based on the multiple-target tracing (MTT) algorithm. The localization and tracking parameters in SPT experiments are listed in the Table 1. The resulting tracks were manually curated and inspected to ensure tracks were accurately reconstructed.

**Table 1.** Localization and tracking parameters for the MTT program.

Localization error	1E-6	
Deflation loops	3	
Maximum number competitors	3	
Maximum diffusion coefficient (µm^2^/s)	1	For GluA1-HT
Maximum diffusion coefficient (µm^2^/s)	3	For GEM

### Hidden Markov modeling with Bayesian model selection (HMM-Bayes) analysis

The diffusion and transport states of individual GluA1-HT and GluA1-HT-SEP trajectories were analyzed with the HMM-Bayes MATLAB program using default parameters. HMM-Bayes classifies each jumping step in a trajectory as either diffusion or active transport and also calculates the diffusion coefficient and velocity of each step. Active transport (AT) is modeled as directed motion (V) with Brownian diffusion (D) according to the equation:

$$<r^{2}>=4D\Delta t+(V\Delta t{)}^{2}$$

We defined the maximum number of unique motion states that can be inferred for a trajectory to be 3 (i.e. a trajectory can be assigned 1, 2, or 3 unique states). Diffusion coefficients determined by HMM-Bayes analysis were validated by comparing these values to diffusion coefficients for the same trajectories determined by MSD curve fitting with MSDanalyzer (Tarantino et al., 2014) in MATLAB (minimal fitting of R^2^=0.8; see Figure 1—figure supplement 5 for more detail). For any multi-state trajectory that exhibited ambiguity in its motion states (i.e. its motion states appeared to differ subjectively from the motion states assigned by HMM-Bayes, and the motion states were predicted by HMM-Bayes with low probability), we segmented the trajectory based on the apparent motion of each segment and reanalyzed each segment with HMM-Bayes and MSDanalyzer.

### Vesicle identification

After reconstructing trajectories for GluA1-HT and GluA1-HT-SEP particles and characterizing their motion, we identified vesicles for further analysis. To identify vesicles, we first removed particles that clearly localized inside spines for the majority of steps in their trajectories. Next, we filtered all particles that exhibited active transport based on HMM-Bayes analysis. For particles that exhibit only diffusive motion, we then filtered out all particles that bleach completely within 100 frames (the maximum number of frames required for a GluA1-HT homotetramer to bleach; Figure 1—figure supplement 6B). Lastly, we removed particles that have diffusion coefficients greater than 0.45 μm^2^/s (i.e. particles on cell surface; Figure 1—figure supplement 6C) or less than 0.02 μm^2^/s (i.e. particles trapped in postsynaptic densities; Figure 1—figure supplement 7). To separate vesicles into zones (e.g. in Figure 2), we first measured the length of the dendritic shaft using the GFP fill. As images were typically centered on uncaging sites, we separated the length of the dendritic shaft into 3 equal sections based on proximity to the uncaging site. For example, if a dendrite is 90 μm long, Zone 1 would be the 30 μm length of dendrite immediate flanking the uncaging site; Zone 2 would be the two 15 μm lengths of dendrite flanking Zone 1; and so forth for Zone 3.

### Chemical stimulation

To determine whether chemical LTP (cLTP) increased the concentration of GluA1-HT on neuronal surfaces, we washed cultured neurons three times with stimulation buffer (150 mM NaCl, 2 mM CaCl_2_, 5 mM KCl, 10 mM HEPES, 30 mM ᴅ-glucose, 20 mM bicuculline, 1 μM strychnine, pH 7.4) and then stimulated neurons with 0.2 mM glycine in stimulation buffer for 15 min at 37 °C and 5% CO_2_. Neurons were rinsed twice with wash buffer (150 mM NaCl, 2 mM CaCl_2_, 2 mM MgCl_2_, 5 mM KCl, 10 mM HEPES, 30 mM ᴅ-glucose, 20 mM bicuculline, 1 μM strychnine, pH 7.4) and then incubated with 25 nM membrane impermeable JF_549_-HTL (JF_549_i-HTL) in wash buffer for 30 min. Cells were then washed three times with wash buffer and fixed as described (see Immunofluorescence). For live-cell GluA1-HT vesicle tracking experiments, the dish was placed onto the stage of a Nikon Eclipse TiE microscope after three washes with stimulation buffer (see Single-particle tracking for imaging setup). After a desired field of view was identified, stimulation buffer was exchanged for 0.2 mM glycine in stimulation buffer. A desired Z plane was identified and imaging commenced immediately. For Jasplakinolide (Jsp) stimulation experiments, neurons were first washed with imaging buffer and placed onto the Nikon Eclipse TiE microscope. After a desired field of view was identified, Jsp in imaging buffer was added to the dish to a final concentration of 0.25 μM. After 10 min of incubation with Jsp, we commenced imaging. For experiments with Latrunculin A (LatA), neurons were pretreated with 1 μM LatA for 10 min. In addition, LatA was added to every buffer for stimulation and imaging. For experiments examining the effect of Nocodazole on the active transport of particles, neurons were pretreated with 500 nM Nocodazole for four hours prior to imaging. 500 nM Nocodazole was also added to imaging buffer during imaging. For myosin V and VI inhibition, a cocktail of 4 μM Pentabromopseudilin (PBP), 10 μM MyoVin-1, and 4 μM of 2,4,6-Triiodophenol (TIP) was added to stimulation buffer during chemical stimulation and photostimulation.

### Photostimulation

For all photostimulation experiments, neurons were washed three times with photostimulation buffer (150 mM NaCl, 2 mM CaCl_2_, 5 mM KCl, 10 mM HEPES, 30 mM ᴅ-glucose, pH 7.4, plus 1 μM Tetrodotoxin; TTX). Photostimulation buffer was then replaced with 2 mM MNI-caged-ʟ-glutamate (MNI) in photostimulation buffer, and samples were placed onto a Nikon Eclipse TiE microscope (see Single-particle tracking for imaging setup). For all MNI uncaging experiments, we used a 405 nm uncaging laser (20 mW at the fiber end,~1 μm spot diameter; Nikon). To calibrate the strength of the 405 nm uncaging laser, we used GCaMP6s (Chen et al., 2013), a genetically encoded calcium indicator, to monitor the influx of calcium in response to MNI uncaging. Neurons expressing GCaMP6s were identified using the FITC channel. The 405 nm uncaging laser was parked 1 μm from the tip of a dendritic spine head and fired once for 0.1 ms to determine whether the laser power was sufficient to evoke calcium influx at the targeted spine, in neighboring spines, and in the dendritic shaft. After performing uncaging once and recording the calcium influx, we moved to another spine on another neuron and repeated uncaging. We varied the laser power between 10 and 100% to determine the laser power that would maximally activate GCaMP6s only in the targeted spine. For glutamate uncaging-evoked sLTP experiments, we used neurons expressing GFP and GluA1-HT (or just GluA1-HT-SEP for exocytosis experiments). We selected dendritic spines to be targeted for glutamate uncaging-evoked sLTP based on their morphology in the FITC channel. We then positioned the 405 nm uncaging laser 1 μm from the tip of the dendritic spine head. sLTP was stimulated by firing the 405 nm uncaging laser at 1 Hz for 50 s with a pulse width of 0.1 ms. After photostimulation, we checked whether the dendritic spine head of interest had expanded in area in the FITC channel. To examine the effect of sLTP on GluA1-HT vesicle motion, we performed timelapse imaging on GluA1-HT vesicles in the TRITC channel (see Single-particle tracking) immediately before and after sLTP stimulation. For experiments using GluA1-HT-SEP, we identified spines for stimulation using the Cy5 channel (i.e. GluA1-HT-JF_646_) due to the loss of the FITC channel to SEP. To account for photobleaching and determine the adjusted number of vesicles after photostimulation, we determined the vesicle counts before and after sLTP stimulation in no MNI controls. We then used the fold loss in vesicles after sLTP stimulation in no MNI controls to correct the number of vesicles after sLTP stimulation across all conditions.

### Tractin imaging

To examine the effect of cLTP on the length of F-actin fibers in the dendritic shaft, cultured rat hippocampal neurons expressing tractin were imaged using an inverted Carl Zeiss LSM 880 microscope (see Immunofluorescence) with a 63 x Plan Apochromat oil-immersion objective (NA = 1.4). To achieve higher resolution, emitted photons were collected with a 32-channel spectral GaAsP PMT for Airyscan processing. As these experiments were performed on live cells, a PeCon Incubator XL enclosure with Lab-Tek S1 heating insert and CO_2_ lid were used to maintain constant environmental conditions of 5% CO_2_ and 37 °C during imaging. Prior to imaging, neurons expressing tractin were washed three times with stimulation buffer (see Chemical stimulation) and placed onto the Zeiss LSM 880 stage. A dendrite of interest expressing tractin was identified and a z-stack of the dendrite acquired. After acquisition of the dendrite before cLTP (pre-cLTP), stimulation buffer was exchanged for 0.2 mM glycine in stimulation buffer. After 15 min, a z-stack of the dendrite (post-cLTP) was acquired with identical imaging parameters. Airyscan processing was applied to both the pre- and post-cLTP images. The F-actin skeletons were then quantified as described in Figure 3—figure supplement 1. Briefly, we improved signal-to-noise by applying background subtraction and a minimum filter to the raw images. We then masked tractin signals in spines. We used Otsu’s method to threshold filaments and generate a binary mask, which we then skeletonized. Finally, we measured the length of the skeleton using the AnalyzeSkeleton ImageJ plugin (Arganda-Carreras et al., 2010).

### Phalloidin labeling

Neurons were fixed and permeabilized as described above (see Immunofluorescence), except MgCl_2_ and CaCl_2_ were removed from all buffers. Neurons were then blocked for 1 hr in 0.1% Triton X-100 blocking buffer (3% bovine serum albumin and 0.1% Triton X-100 in PBS) at room temperature. F-actin was then labeled with 0.13 μM Abberior Star 635 p conjugated phalloidin (Abberior) in 0.1% Triton X-100 blocking buffer for 1 hr at room temperature. After labeling, neurons were washed once with PBS and mounted in MOWOIL-DABCO. Superresolution images were acquired using an Abberior Expert Line STED microscope (see Immunofluorescence). While STED imaging greatly improved resolution, it also reduced the strength of F-actin signals. Consequently, we could not use the method described above (see Tractin imaging and Figure 3—figure supplement 1) to measure the length of F-actin fibers labeled with phalloidin. Instead, we masked phalloidin signal in spines using the Intermodes method - a method for automatic intensity thresholding - and measured the mean fluorescence intensity of phalloidin inside the dendritic shaft.

### Radius of confinement and directional bias analysis

To determine the directional bias of a trajectory using angle analysis, we implemented a custom MATLAB program. Specifically, for a given trajectory, we defined the radius of confinement as the distance from the centroid of a trajectory to the furthest point in the trajectory to the centroid. Next, we created a mask around the dendrite in which the trajectory is localized and set the dendritic path along the length of the dendrite. We then determined the point on the dendritic path that is closest to the centroid of the trajectory. The angle, theta Θ, created by these three points (the point on the dendritic path closest to the centroid, the centroid itself, and the point on the trajectory furthest from the centroid) indicates whether the motion of the trajectory is biased *towards* the length of the dendritic path or *along* the length of the dendritic path. Theta ~ 0^o^ indicates that the motion of the trajectory is biased perpendicular to the dendritic path (i.e. lateral motion) while theta ~ 90^o^ indicates that the motion of the trajectory is biased parallel to the dendritic path (i.e. longitudinal motion).

### Electrophysiology

Whole-cell voltage-clamp recordings were performed to determine the preservation of GluA1-HT and GluA1-HT-SEP functionality. Cultured hippocampal neurons were assessed at DIV7-8. Labeling GluA1-HT/HT-SEP with JF_549_-HTL was performed 12 hr prior to patch clamp experiments. GluA1-HT/HT-SEP positive and negative neurons were identified via epifluorescence. Whole-cell configuration was achieved using pipettes pulled and polished to a resistance of 2–5 MΩ. SylGard 184 coating was applied to reduce pipette capacitance. Pipette solution composition included 120 mM Cs-MES, 5 mM NaCl, 10 mM TEA-Cl, 5 mM Lidocaine, 1.1 mM EGTA, 10 mM HEPES, 0.3 mM Na-GTP, 4 mM Mg-ATP. External solution contained 135 mM NaCl, 3 mM KCl, 2 mM MgCl_2_, 1.5 mM CaCl_2_, 10 mM TEA-Cl, 10 mM HEPES, 10 mM Glucose. The external solution was supplemented with 200 nM TTX and 10 µM AP5. Cell capacitance was estimated and corrected to 80% with a 10 µsec lag. Gap free voltage clamp recordings were performed at 100 kHz acquisition frequency with a 1.0 kHz Bessel filter. Voltage was clamped to –70 mV, correcting for liquid junction potential. Following baseline measurements, 100 µM glutamate in pH adjusted external solution was locally perfused for 5 s using a fused silicate pipe positioned within 100–200 µm of the neuron recorded. The neuron then recovered with continued bath perfusion and local perfusion of glutamate-free external solution. For analysis, the gap-free epochs were reduced 50-fold. The data was normalized to current density via cell capacitance estimations. Peak current density for each response was determined. To determine whether GluA1-HT and GluA1-HT-SEP were functional using a heterologous expression system that does not express GluA1, HEK293T cells were transfected to express GluA1-P2A-GFP, GluA1-HT-P2A-GFP, or GluA1-HT-SEP-P2A-Scarlet (see Cell culture and transfection). After 48 hours of recovery, voltage-clamp recordings of glutamate induced GluA1 currents were performed similarly to the above described neuronal recordings. Patch pipettes pulled to 2–4 MΩ resistance were filled with internal solution containing 2.8 mM KCl, 140 mM NaCl, 2 mM MgCl_2_, 2 mM CaCl_2_, 12 mM glucose, and 10 mM HEPES at pH 7.3. After whole-cell access was achieved, a brief baseline recording of external bath solution was performed; the external solution was comprised of 140 mM KCl, 5 mM MgCl_2_, 5 mM EGTA, and 10 mM HEPES at pH 7.3. Due to the propensity of glutamate contamination in immortalized cell-line cultures, we anticipated desensitization of functionally expressed GluA1. To re-sensitized GluA1, we performed 2 min of constant, local perfusion of external solution supplemented with 50 µM cyclothiazide. After the cyclothiazide pretreatment, 2 mM glutamate with 50 µM cyclothiazide was locally applied to the patched cell for 10 s; this stimulation was followed by a washout period with normal external solution. Currents were adjusted to the control-bath baseline and normalized via cell capacitance. Data is reported as peak glutamate induced current.

### Statistical analysis and experimental replicates

All statistical tests and correlation analysis were performed in GraphPad Prism, version 8. Specific statistical tests to determine the significance of results are indicated in the figure legends. Histograms and probability functions were generated using the Distribution Fitter Application (Statistics and Machine Learning toolbox) in MATLAB. For experiments where GluA1-HT particles were tracked after chemical stimulation (e.g. cLTP induction), particles were tracked in one segment of a dendrite and then the neuronal culture was discarded. In other words, each timelapse is of a dendrite from an independent neuronal culture. This was done to roughly synchronize the start of each timelapse after chemical stimulation. For all MNI uncaging experiments, one spine was targeted per neuron (i.e. we identified a spine on a new neuron for each sLTP stimulation experiment). A maximum of three spines (i.e. three neurons) were targeted for MNI uncaging in one culture before the culture was discarded. Targeted spines were separated by at least 3 mm to minimize the possibility that a spine was exposed to uncaged MNI from a previous experiment. Typically, two to three neuronal cultures were prepared from a single dissection. Consequently, experimental replicates were performed on neurons derived from at least two rats. Nevertheless, we found no significant difference in either the fraction of GluA1-HT vesicles exhibiting active transport or the mean diffusion coefficient of GluA1-HT vesicles between neurons derived from different dissections under a single condition (Figure 1—figure supplement 5F–G). In other words, variation stemming from technical and biological replicates was significantly less than variation due to treatments.

### Materials availability

Newly created materials, including custom MATLAB codes, for this study are available upon request from the corresponding author.

## Data Availability

All data generated or analyzed during this study are included in the manuscript and supporting files, source data files have been provided for all figures.

## Additional files

MDAR checklist

## Appendix 1

**Appendix 1—key resources table:**

Reagent type (species) or resource	Designation	Source or reference	Identifiers	Additional information
Antibody	anti-AMPA receptor 1 (GluA1; Extracellular; Guinea pig polyclonal)	Alomone Labs	AGP-009	IF(1:250)
Antibody	anti-Chicken IgY, Alexa Fluor Plus 647 (Goat polyclonal)	Thermo Fisher Scientific	A-32933	IF(1:500)
Antibody	anti-Chicken IgY, Star Orange (Goat polyclonal)	abberior GmbH	STORANGE-1005–500 UG	IF(1:500)
Antibody	anti-Chicken IgY, Star Red (Goat polyclonal)	abberior GmbH	STRED-1005–500 UG	IF(1:500)
Antibody	anti-GluA1 (Mouse monoclonal)	Synaptic Systems	182 011	IF(1:500)
Antibody	anti-GluN1 (Mouse monoclonal)	Synaptic Systems	114 011	IF(1:1000)
Antibody	anti-Guinea Pig, Alexa Fluor 488 (Goat polyclonal)	Thermo Fisher Scientific	A-11073	IF(1:500)
Antibody	anti-Guinea Pig IgG, Alexa Fluor 555 (Goat polyclonal)	Thermo Fisher Scientific	A-21435	IF(1:500)
Antibody	anti-Guinea Pig IgG, Alexa Fluor 594 (Goat polyclonal)	Thermo Fisher Scientific	A-11076	IF(1:500)
Antibody	anti-Guinea Pig IgG, Alexa Fluor 647 (Goat polyclonal)	Thermo FIsher Scientific	A-21450	IF(1:500)
Antibody	anti-HaloTag (Rabbit polyclonal)	Promega	G9281	IF(1:500)
Antibody	anti-Homer1 (Chicken polyclonal)	Synaptic Systems	160 006	IF(1:1000)
Antibody	anti-Mouse IgG, Alexa Fluor 488 (Goat polyclonal)	Thermo Fisher Scientific	A-11001	IF(1:500)
Antibody	anti-Mouse IgG, Alexa Fluor 546 (Goat polyclonal)	Thermo Fisher Scientific	A-11030	IF(1:500)
Antibody	anti-Mouse IgG, Alexa Fluor 594 (Goat polyclonal)	Thermo Fisher Scientific	A-32742	IF(1:500)
Antibody	anti-Mouse IgG, Alexa Fluor 647 (Goat polyclonal)	Thermo Fisher Scientific	A-21236	IF(1:500)
Antibody	anti-Mouse IgG, Atto 647 N (Goat polyclonal)	Rockland Immunochemicals Inc.	610-156-121	IF(1:500)
Antibody	anti-PSD-95 (7E3-1B8; Mouse monoclonal)	Thermo Fisher Scientific	MA1-046	IF(1:1000)
Antibody	anti-PSD-95 (Guinea pig polyclonal)	Synaptic Systems	124 014	IF(1:2000)
Antibody	anti-Rabbit IgG, Alexa Fluor 488 (Goat polyclonal)	Thermo Fisher Scientific	A-11034	IF(1:500)
Antibody	anti-Rabbit IgG, Alexa Fluor 546 (Goat polyclonal)	Thermo Fisher Scientific	A-11035	IF(1:500)
Antibody	anti-Rabbit IgG, Alexa Fluor 647 (Goat polyclonal)	Thermo Fisher Scientific	A-21245	IF(1:500)
Antibody	anti-Rabbit IgG, Atto 594 (Goat polyclonal)	Rockland Immunochemicals Inc.	611-155-122	IF(1:500)
Antibody	anti-Rabbit IgG, Star Red (Goat polyclonal)	abberior GmbH	STRED-1002–500 UG	IF(1:500)
Cell line (*Homo sapiens*)	Human embryonic kidney (HEK) 293T	ATCC	CRL-3216	
Chemical compound, drug	2,4,6-Triiodophenol (TIP)	Thermo Fisher Scientific	AAA1714506	4 μM
Chemical compound, drug	20 x SSC	Thermo Fisher Scientific	AM9763	
Chemical compound, drug	Cycloheximide (CHX)	Sigma-Aldrich	C4859	50 nM
Chemical compound, drug	DABCO	Sigma-Aldrich	D27802	
Chemical compound, drug	D-AP5	Tocris	0106	10 μM
Chemical compound, drug	Dimethyl sulfoxide (DMSO), Anhydrous	Thermo Fisher Scientific	D12345	
Chemical compound, drug	Glycine	Tocris	0219	0.2 mM
Chemical compound, drug	Jasplakinolide (Jsp)	Tocris	2792	
Chemical compound, drug	JF_549_-HTL	Grimm et al., 2015	N/A	10 nM
Chemical compound, drug	JF_549_i-HTL	Xie et al., 2017	N/A	10 nM
Chemical compound, drug	JF_646_-HTL	Grimm et al., 2015	N/A	20 nM
Chemical compound, drug	Latrunculin A (LatA)	Tocris	3973	1 μM
Chemical compound, drug	Lipofectamine 3000	Thermo Fisher Scientific	L3000001	
Chemical compound, drug	MNI-caged-L-glutamate (MNI)	Tocris	14-905-0	2 mM
Chemical compound, drug	Mowoil 4–88	Sigma-Aldrich	813831	
Chemical compound, drug	MyoVin-1	Sigma-Aldrich	475984	10 μM
Chemical compound, drug	NbActiv4 Minus Phenol Red	Transnetyx	NB4PR500	
Chemical compound, drug	Nocodazole	Tocris	1228	500 nM
Chemical compound, drug	Normal Goat Serum	Thermo Fisher Scientific	31873	
Chemical compound, drug	Papain	Worthington Biochemical Corporation	LK003176	
Chemical compound, drug	Paraformaldehyde (PFA)	Electron Microscopy Sciences	15713	
Chemical compound, drug	Pentabromopseudilin (PBP)	Sigma-Aldrich	SML2428	4 μM
Chemical compound, drug	Phalloidin Star 635 p	abberior GmbH	ST635P-0100–20 UG	0.17 μM
Chemical compound, drug	Poly-ᴅ-Lysine (PDL) Hydrobromine	Sigma-Aldrich	P6407	
Chemical compound, drug	ProLong Diamond Antifade Mountant with DAPI	Thermo Fisher Scientific	P36971	
Chemical compound, drug	Saponin	Sigma-Aldrich	47036	
Chemical compound, drug	Tetrodotoxin Citrate (TTX)	Tocris	1069	200 nM
Chemical compound, drug	Triton X-100	Sigma-Aldrich	T8787	
Commercial assay or kit	HCR RNA-FISH Bundle for HaloTag and *gria1* mRNA	Molecular Instruments	N/A	
Commercial assay or kit	P3 Primary Cell 96-well Nucleofector Kit (96 RCT)	Lonza	V4SP-3096	
Recombinant DNA reagent	CAG-tdTomato (plasmid)	James Wilson	Addgene #105554	
Recombinant DNA reagent	hSyn-GEM (plasmid)	This paper	N/A	Plasmid for expressing GEM-HT-ST in neurons
Recombinant DNA reagent	hSyn-GFP (plasmid)	James Wilson	Addgene #105539	
Recombinant DNA reagent	hSyn-tractin (plasmid)	This paper	N/A	Plasmid for expressing F-tractin-mNeongreen in neurons
Recombinant DNA reagent	ITPKA-mNeongreen (plasmid)	Chertkova et al., 2017	Addgene # 98883	
Recombinant DNA reagent	LZ10 PBREBAC-H2BHalo (plasmid)	Li et al., 2016	Addgene #91564	
Recombinant DNA reagent	pAS1NB c Rosella I (plasmid)	Rosado et al., 2008	Addgene #71245	
Recombinant DNA reagent	pBAD/His-miRFP670 (plasmid)	Shcherbakova et al., 2016	Addgene #79884	
Recombinant DNA reagent	pCAGGS (plasmid)	Niwa et al., 1991	BCCM/LMBP #2453	
Recombinant DNA reagent	pCAG-GluA1-P2A-GFP (plasmid)	This paper	N/A	Plasmid for expressing GluA1-P2A-GFP in HEK293T cells for patching assays
Recombinant DNA reagent	pCAG-GluA1-HT-P2A-GFP (plasmid)	This paper	N/A	Plasmid for expressing GluA1-HT-P2A-GFP in HEK293T cells for patching assays
Recombinant DNA reagent	pCAG-GluA1-HT-SEP-P2A-mScarlet (plasmid)	This paper	N/A	Plasmid for expressing GluA1-HT-SEP-P2A-mScarlet in HEK293T cells for patching assays
Recombinant DNA reagent	pCMV-PfV-Sapphire-IRES-DSRed (plasmid)	Delarue et al., 2018	Addgene #116934	
Recombinant DNA reagent	pCMV2-SEP-GluA1 (plasmid)	Blanco-Suarez and Hanley, 2014	Addgene #64942	
Recombinant DNA reagent	pEGFP-C1 (plasmid)	N/A	Clontech	
Recombinant DNA reagent	pEGFP-MyosinVa-Ctail (plasmid)	Correia et al., 2008	Addgene #110169	
Recombinant DNA reagent	pEGFP-MyosinVb-Ctail (plasmid)	Correia et al., 2008	Addgene #110170	
Recombinant DNA reagent	pEGFP-MyosinVI-Ctail (plasmid)	This paper	N/A	Plasmid for expressing the dominant-negative myosin VI C-tail to inhibit myosin VI function
Recombinant DNA reagent	PX551 (plasmid)	Swiech et al., 2015	Addgene #60957	
Recombinant DNA reagent	PX552 (plasmid)	Swiech et al., 2015	Addgene #60958	
Recombinant DNA reagent	px552-sg-gria1-HT (plasmid)	This paper	N/A	The donor plasmid for knocking HaloTag into *Gria1* via HITI
Recombinant DNA reagent	px552-sg-gria1-HT-SEP (plasmid)	This paper	N/A	The donor plasmid for knocking HaloTag-SEP into *Gria1* via HITI
Recombinant DNA reagent	Cas9 target sequence for sgRNA insert	Integrated DNA Technologies	N/A	5'-ACCCTCCCGAG ACCATACCAGGG-3' 5'-AACCCCTGGTATG GTCTCGGGAG-3'
Recombinant DNA reagent	Sequencing primers for 5’ insertion site of HaloTag into *Gria1*	Integrated DNA Technologies	N/A	5’-ATGCCTGCAGGTC GAAGAGACCTACCCA GACGATG-3’ 5’-GTGAATTCGAGCTCG GGCGGATGAACTCCATAAATGC-3’
Recombinant DNA reagent	Sequencing primers for 3’ insertion site of HaloTag into *Gria1*	Integrated DNA Technologies	N/A	5’-ATGCCTGCAGGTCGA TGCATTTATGGAGTTCATCCGC-3’ 5’-GTGAATTCGAGCTCGT GTGTTCTGCTGTTCAGGAG-3’
Software, algorithm	Directional Bias	This paper	N/A	MATLAB script used to determine the directional bias of particles
Software, algorithm	Fiji	Schindelin et al., 2012	N/A	
Software, algorithm	FISH-Quant	Mueller et al., 2013	https://fish-quant.github.io/	
Software, algorithm	HMM-Bayes, v1	Monnier et al., 2015; Bathe BioNanoLab, 2021	https://github.com/lcbb/HMM-Bayes	MATLAB script used to predict motion states of particles
Software, algorithm	Imaris 9.3	Bitplane (Belfast, United Kingdom)	N/A	
Software, algorithm	Impsector	abberior GmbH (Göttingen, Germany)	N/A	
Software, algorithm	MATLAB 2016a	Mathworks (Natick, MA)	https://www.mathworks.com/	
Software, algorithm	MSDanalyzer, v1.3	Tarantino et al., 2014; Tinevez, 2020	https://github.com/tinevez/msdanalyzer	MATLAB script used to determine diffusion coefficients of particles
Software, algorithm	Prism 9, v8	GraphPad (San Diego, CA)	https://www.graphpad.com/	
Software, algorithm	SLIMfast	This paper	N/A	MATLAB program for 2D single-particle tracking
Software, algorithm	Zen Black	Zeiss (Jena, Germany)	N/A	
Strain, strain background (*AAV*)	AAV2/9-CAG-TdTomato	Janelia Research Campus Viral Services	N/A	
Strain, strain background (*AAV*)	AAV2/9-hSyn-GCaMP6s	Janelia Research Campus Viral Services	N/A	
Strain, strain background (*AAV*)	AAV2/9-hSyn-GEM	Janelia Research Campus Viral Services	N/A	
Strain, strain background (*AAV*)	AAV2/9-hSyn-GFP	Janelia Research Campus Viral Services	N/A	
Strain, strain background (*AAV*)	AAV2/9-hSyn-tractin	Janelia Research Campus Viral Services	N/A	
Strain, strain background (*AAV*)	rAAV2-PX551	Janelia Research Campus Viral Services	N/A	
Strain, strain background (*AAV*)	rAAV2-px552-sg-gria1-HT	Janelia Research Campus Viral Services	N/A	
Strain, strain background (*AAV*)	rAAV2-px552-sg-gria1-HT-SEP	Janelia Research Campus Viral Services	N/A	
Strain, strain background (*AAV*)	rh10-PX551	Janelia Research Campus Viral Services	N/A	
Strain, strain background (*AAV*)	rh10-px552-sg-gria1-HT	Janelia Research Campus Viral Services	N/A	
Strain, strain background (*AAV*)	rh10-px552-sg-gria1-HT-SEP	Janelia Research Campus Viral Services	N/A	
Strain, strain background (*Rattus norvegicus*)	Sprague-Dawley rats, CD1 (Sprague-Dawley postnatal pups P0, M and F)	Charles River Laboratories	Strain code: 400	
Strain, strain background (*Escherichia coli*)	Stbl3	Invitrogen	C737303	
